# Novel Oleanane-Type Triterpene Glycosides from the *Saponaria officinalis* L. Seeds and Apoptosis-Inducing Activity via Mitochondria

**DOI:** 10.3390/ijms23042047

**Published:** 2022-02-12

**Authors:** Naoki Takahashi, Tomoki Iguchi, Minpei Kuroda, Masaki Mishima, Yoshihiro Mimaki

**Affiliations:** School of Pharmacy, Tokyo University of Pharmacy and Life Sciences, 1432-1, Horinouchi, Hachioji, Tokyo 192-0392, Japan; y20612@toyaku.ac.jp (N.T.); kurodam@toyaku.ac.jp (M.K.); mmisima@toyaku.ac.jp (M.M.); mimakiy@toyaku.ac.jp (Y.M.)

**Keywords:** *Saponaria officinalis*, Caryophyllaceae, seed, triterpene glycoside, cytotoxicity, apoptosis, mitophagy, HL-60 cell, A549 cell, SBC-3 cell

## Abstract

*Saponaria officinalis* L., commonly known as “Soapwort”, is a rich source of triterpene glycosides; however, the chemical constituents of *S. officinalis* seeds have not been fully identified. In this study, we conducted a systematic phytochemical investigation of the seeds of *S. officinalis* and obtained 17 oleanane-type triterpene glycosides (**1**–**17**), including seven new glycosides (**1**–**7**). The structures of **1**–**7** were determined based on a detailed analysis of NMR spectroscopic data and chromatographic and spectroscopic analyses following specific chemical transformation. The cytotoxicities of the isolated compounds were evaluated against HL-60 human promyelocytic leukemia cells, A549 human adenocarcinoma lung cancer cells, and SBC-3 human small-cell lung cancer cells. The cytotoxicities of **1**, **4**, and **10** toward HL-60 cells and SBC-3 cells were nearly as potent as that of cisplatin. Compound **1**, a bisdesmosidic triterpene glycoside obtained in good yield, arrested the cell cycle of SBC-3 cells at the G_2_/M phase, and induced apoptosis through an intrinsic pathway, accompanied by ROS generation. As a result of the mitochondrial dysfunction induced by **1**, mitochondria selective autophagy, termed mitophagy, occurred in SBC-3 cells.

## 1. Introduction

During our ongoing phytochemical investigations of higher plants with a focus on new cytotoxic constituents, we have isolated cytotoxic triterpene glycosides from *Dolichos lablab* [1], *Stryphnodendron fissuratum* [2], *Eranthis cilicica* [3], *Larrea tridentata* [4], *Anemone hupehensis* var. *japonica* [5], *Caulophyllum thalictroides* [6], and *Clematis chinensis* [7]. The genus *Saponaria* (Caryophyllaceae) includes approximately 30 species with distribution in Europe and western Asia. *Saponaria officinalis* L., commonly known as “Soapwort”, is a perennial plant and is now cultivated mainly for ornamental purpose. Radix Saponariae, the dried roots of *S. officinalis*, has been used as a folk medicine for expectorant, syphilis, skin complaints, and rheumatic disorders [8,9,10]. Previous phytochemical investigations of *S. officinalis* resulted in the isolation of highly polar triterpene glycosides, the sugar moieties of which comprise 3–10 monosaccharides [11,12,13,14,15,16], indicating that *S. officinalis* is a rich source of triterpene glycosides. However, a literature survey indicated that a systematic phytochemical examination of the seeds of *S. officinalis* has not yet been conducted.

In the statistical data for the ten-year relative survival of Japanese cancer patients between 2002 and 2006, the survival rates of lung cancer and leukemia patients were 18.1% and 20.5% for males (aged 15 to 99), and 31.2% and 20.5% for females (aged 15 to 99), respectively [17]. Although lung cancer and leukemia patients successfully go into remission, they are not satisfied with current long-term medical treatment owing to relatively high-frequency recurrence. Small-cell lung cancer is generally treated with cisplatin, etoposide, irinotecan, paclitaxel, and vincristine, while few molecularly targeted drugs or immune checkpoint inhibitors have been approved. The above anticancer agents frequently cause severe undesirable side effects such as myelosuppression, vomit, acute kidney injury, peripheral neuropathy, hepatic dysfunction, and diarrhea, which deterrent the quality of life of patients. Thus, the development of new anticancer agents is expected to improve the survival rates of cancer patients and reduce undesirable side effects.

In our phytochemical investigation of *S. officinalis* seeds, we focused on triterpene glycosides and isolated 17 glycosides (**1**–**17**), including seven new ones (**1**–**7**). The structures of **1**–**7** were elucidated based on extensive analyses of NMR spectroscopic data, and chromatographic and spectroscopic analyses following chemical transformations. The cytotoxic activities of the isolated compounds were examined against HL-60 human promyelocytic leukemia cells, A549 human adenocarcinoma lung cancer cells, and SBC-3 human small-cell lung cancer cells. Furthermore, the apoptosis-inducing activity of **1** in SBC-3 cells was evaluated.

## 2. Results and Discussion

### 2.1. Structure Characterization of ***1**–**17***

**Figure 1:**
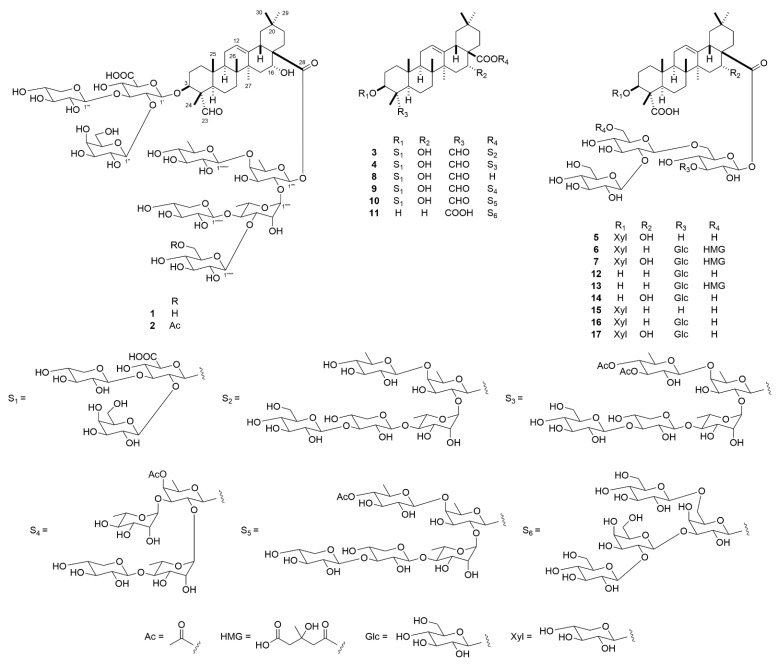
Structures of **1**–**17**. Compounds **1**–**7**, **9**, **10**, **15**–**17** are bisdesmosidic triterpene glycosides, among which **1**–**7** are previously undescribed.

The seeds of *S. officinalis* (1.0 kg) were extracted using MeOH (60 °C), and then the solvent was removed under reduced pressure. The MeOH extract (50 g) was subjected to silica gel and octadecylsilanized (ODS) silica gel column chromatography (CC), and preparative ODS high-performance liquid chromatography (HPLC) to obtain 17 compounds (**1**–**17**) (Figure 1). Compounds **8**–**17** were identified as 3β-[(*O*-β-d-galactopyranosyl-(1→2)-*O*-[β-d-xylopyranosyl-(1→3)]-β-d-glucuronopyranosyl)oxy]-16α-hydroxy-23-oxo-olean-12-en-28-oic acid (**8**) [18], 3β-[(*O*-β-d-galactopyranosyl-(1→2)-*O*-[β-d-xylopyranosyl-(1→3)]-β-d-glucuronopyranosyl)oxy]-16α-hydroxy-23-oxo-olean-12-en-28-oic acid *O*-β-d-xylopyranosyl-(1→4)-*O*-α-l-rhamnopyranosyl-(1→2)-*O*-[α-l-rhamnopyranosyl-(1→3)]-4-*O*-acetyl-β-d-fucopyranosyl ester (**9**) [19], 3β-[(*O*-β-d-galactopyranosyl-(1→2)-*O*-[β-d-xylopyranosyl-(1→3)]-β-d-glucuronopyranosyl)oxy]-16α-hydroxy-23-oxo-olean-12-en-28-oic acid *O*-β-d-xylopyranosyl-(1→3)-*O*-β-d-xylopyranosyl-(1→4)-*O*-α-l-rhamnopyranosyl-(1→2)-*O*-[4-*O*-acetyl-β-d-quinovopyranosyl-(1→4)]-β-d-fucopyranosyl ester (saponarioside B) (**10**) [16], 3β-hydroxyolean-12-en-23,28-dioic acid 28-*O*-β-d-glucopyranosyl-(1→2)-*O*-β-d-galactopyranosyl-(1→3)-*O*-[β-d-glucopyranosyl-(1→6)]-β-d-galactopyranosyl ester (**11**) [20], 3β-hydroxyolean-12-en-23,28-dioic acid 28-*O*-β-d-glucopyranosyl-(1→2)-*O*-β-d-glucopyranosyl-(1→6)-*O*-[β-d-glucopyranosyl-(1→3)]-β-d-glucopyranosyl ester (vaccaroid A) (**12**) [21], 3β-hydroxyolean-12-en-23,28-dioic acid 28-*O*-β-d-glucopyranosyl-(1→2)-*O*-(6-*O*-(3-hydroxy-3-methylglutaryl)-β-d-glucopyranosyl)-(1→6)-*O*-[β-d-glucopyranosyl-(1→3)]-β-d-glucopyranosyl ester (vaccaroside B) (**13**) [22], 3β,16α-dihydroxyolean-12-en-23,28-dioic acid 28-*O*-β-d-glucopyranosyl-(1→2)-*O*-β-d-glucopyranosyl-(1→6)-*O*-[β-d-glucopyranosyl-(1→3)]-β-d-glucopyranosyl ester (segetoside K) (**14**) [23], 3β-[(β-d-xylopyranosyl)oxy]-olean-12-en-23,28-dioic acid 28-*O*-β-d-glucopyranosyl-(1→2)-*O*-β-d-glucopyranosyl-(1→6)-β-d-glucopyranosyl ester (saponarioside M) (**15**) [14], 3β-[(β-d-xylopyranosyl)oxy]-olean-12-en-23,28-dioic acid 28-*O*-β-d-glucopyranosyl-(1→2)-*O*-β-d-glucopyranosyl-(1→6)-*O*-[β-d-glucopyranosyl-(1→3)]-β-d-glucopyranosyl ester (saponarioside D) (**16**) [15], and 16α-hydroxy-3β-[(β-d-xylopyranosyl)oxy]olean-12-en-23,28-dioic acid 28-*O*-β-d-glucopyranosyl-(1→2)-*O*-β-d-glucopyranosyl-(1→6)-*O*-[β-d-glucopyranosyl-(1→3)]-β-d-glucopyranosyl ester (saponarioside F) (**17**) [15], respectively.

Compound **1** was obtained as an amorphous solid, and its molecular formula was assigned as C_76_H_120_O_41_ based on the accurate sodium adduct ion at *m/z* 1711.7161 [M + Na]^+^ (calculated for C_76_H_120_NaO_41_: 1711.7203) using high-resolution electrospray ionization time-of-flight mass spectroscopy (HR-ESI-TOF-MS) and ^13^C-NMR spectral data. In the ^1^H- and ^13^C-NMR spectra of **1**, the following signals were observed: six tertiary methyl groups [δ_H_ 1.73 (s, Me-27), 1.46 (s, Me-24), 1.10 (s, Me-26), 0.93 (s, Me-30), 0.92 (s, Me-29), and 0.84 (s, Me-25); δ_C_ 33.1 (C-29), 27.1 (C-27), 24.4 (C-30), 17.4 (C-26), 15.9 (C-25), and 11.1 (C-24)], two oxygenated methine groups [δ_H_ 5.11 (br s, H-16) and 4.06 (m, H-3); δ_C_ 84.4 (C-3) and 74.2 (C-16)], an olefinic group [δ_H_ 5.50 (m, H-12); δ_C_ 144.5 (C-13) and 121.9 (C-12)], an aldehyde group [δ_H_ 9.91 (s, H-23); δ_C_ 210.1 (C-23)], an ester carbonyl carbon [δ_C_ 175.9 (C-28)], six quaternary carbons [δ_C_ 55.0 (C-4), 49.2 (C-17), 42.1 (C-14), 40.3 (C-8), 36.2 (C-10), and 30.6 (C-20)], and eight anomeric protons/carbons [δ_H_ 6.00 (br s), 5.88 (d, *J* = 8.2 Hz), 5.54 (d, *J* = 7.7 Hz), 5.50 (d, *J* = 7.9 Hz), 5.39 (d, *J* = 7.7 Hz), 5.31 (d, *J* = 7.7 Hz), 4.98 (d, *J* = 7.9 Hz), and 4.89 (d, *J* = 7.8 Hz); δ_C_ 106.3, 105.2, 105.0, 104.9, 104.2, 103.9, 101.7, and 94.8]. Treatment of **1** with 1 M KOH in MeOH yielded **8** and the sugar fraction, and subsequent acid hydrolysis of the sugar fraction using 1 M HCl in 1,4-dioxane/H_2_O (1:1) afforded d-fucose, d-glucose, d-quinovose, l-rhamnose, and d-xylose. The monosaccharides were identified based on HPLC analysis using a combination of optical rotation and refractive index detectors. As the sugar moieties of **1** were composed of eight monosaccharides, their numerous proton signals overlapped severely, precluding their assignment using conventional NMR analysis. To address this issue, one-dimensional total correlation spectroscopy (1D-TOCSY) and heteronuclear single quantum coherence (HSQC)-TOCSY spectroscopy in addition to ^1^H-^1^H correlation spectroscopy (COSY) and HSQC spectroscopy were applied. The ^1^H-NMR subspectra of individual glycosyl units were obtained by selective irradiation of each anomeric proton signal and other non-overlapping proton signals. Subsequent analysis of the ^1^H-^1^H COSY spectrum allowed for the sequential assignment of the proton resonances for the pentaglycosyl moiety attached to C-28 of the aglycone, enabling us to identify their multiplet patterns and coupling constants. The HSQC and HSQC-TOCSY spectra correlated the proton signals with the corresponding one-bond-coupled carbon shifts. The assigned ^1^H- and ^13^C-NMR signals indicated that the C-28 pentaglycosyl moiety consisted of a 2,4-disubstituted β-d-fucopyranosyl unit [Fuc: δ_H_ 5.88 (d, *J* = 8.2 Hz, H-1’’’’ of Fuc); δ_C_ 94.8, 75.2, 75.9, 83.4, 71.5, and 17.1 (C-1’’’’–C-6’’’’ of Fuc)], a 3,4-disubstituted α-l-rhamnopyranosyl unit [Rha: δ_H_ 6.00 (br s, H-1’’’’’ of Rha); δ_C_ 101.7, 70.8, 82.3, 78.2, 68.9, and 18.9 (C-1’’’’’–C-6’’’’’ of Rha)], a terminal β-d-glucopyranosyl unit [Glc: δ_H_ 5.39 (d, *J* = 7.7 Hz, H-1’’’’’’ of Glc); δ_C_ 105.2, 75.4, 78.3, 71.7, 78.4, and 62.6 (C-1’’’’’’–C-6’’’’’’ of Glc)], a terminal β-d-xylopyranosyl unit [Xyl (II): δ_H_ 5.50 (d, *J* = 7.9 Hz, H-1’’’’’’’ of Xyl (II)); δ_C_ 104.9, 75.6, 79.1, 71.2, and 67.1 (C-1’’’’’’’–C-5’’’’’’’ of Xyl (II))], and a terminal β-d-quinovopyranosyl unit [Qui: δ_H_ 4.98 (d, *J* = 7.9 Hz, H-1’’’’’’’’ of Qui); δ_C_ 106.3, 75.7, 78.1, 76.7, 73.1, and 18.5 (C-1’’’’’’’’–C-6’’’’’’’’ of Qui)]. The β-orientations of the anomeric centers of Fuc, Glc, Xyl, and Qui were determined based on the relatively large ^3^*J*_H-1,H-2_ values. The large ^1^*J*_H-1,C-1_ value (171 Hz) of Rha suggested that the anomeric configuration of Rha was α-oriented [16]. In the heteronuclear multiple bond correlation (HMBC) spectrum of **1**, ^3^*J*_C,H_ correlations were observed between H-1’’’’’’ of Glc and C-3’’’’’ of Rha, H-1’’’’’’’ of Xyl (II) and C-4’’’’’ of Rha, H-1’’’’’ of Rha and C-2’’’’ of Fuc, H-1’’’’’’’’ of Qui and C-4’’’’ of Fuc, and between H-1’’’’ of Fuc and C-28 of the aglycone. Thus, **1** was determined to be 3β-[(*O*-β-d-galactopyranosyl-(1→2)-*O*-[β-d-xylopyranosyl-(1→3)]-β-d-glucuronopyranosyl)oxy]-16α-hydroxy-23-oxo-olean-12-en-28-oic acid *O*-β-d-glucopyranosyl-(1→3)-*O*-[β-d-xylopyranosyl-(1→4)]-*O*-α-l-rhamnopyranosyl-(1→2)-*O*-[β-d-quinovopyranosyl-(1→4)]-β-d-fucopyranosyl ester. Although **1** has been described as a possible putative model compound of cell-targeting binding molecules in a patent [24], this is the first time it has been isolated from a natural source and its structure determined based on extensive NMR spectroscopic data.

The ^1^H- and ^13^C-NMR spectra of **2** (C_78_H_122_O_42_) were similar to those of **1**. However, the molecular formula of **2** contained an additional C_2_H_2_O, and signals arising from an acetyl group [δ_H_ 2.14 (s); δ_C_ 170.9 and 21.0] were observed in the ^1^H- and ^13^C-NMR spectra of **2**. These data suggest that **2** is a monoacetylated derivative of **1**. The HMBC spectrum of **2** exhibited long-range correlations from H_2_-6’’’’’’ of Glc (δ_H_ 4.91 and 4.65) to the carbonyl carbon of the acetyl moiety (δ_C_ 170.9). Comparing the ^13^C-NMR spectrum of **2** with that of **1**, the signal attributable to C-6’’’’’’ of Glc was shifted downfield by 1.8 ppm. The above data indicate that the acetyl group is attached to C-6’’’’’’ of Glc. Therefore, **2** was identified as 3β-[(*O*-β-d-galactopyranosyl-(1→2)-*O*-[β-d-xylopyranosyl-(1→3)]-β-d-glucuronopyranosyl)oxy]-16α-hydroxy-23-oxo-olean-12-en-28-oic acid *O*-(6-*O*-acetyl-β-d-glucopyranosyl)-(1→3)-*O*-[β-d-xylopyranosyl-(1→4)]-*O*-α-l-rhamnopyranosyl-(1→2)-*O*-[β-d-quinovopyranosyl-(1→4)]-β-d-fucopyranosyl ester.

Compound **3** was obtained as an amorphous solid, and its molecular formula was determined to be C_76_H_120_O_41_ based on the accurate sodium adduct ion at *m/z* 1711.7191 [M + Na]^+^ (calculated for C_76_H_120_NaO_41_: 1711.7203) in the HR-ESI-TOF-MS as well as the ^13^C-NMR spectral data. The ^1^H- and ^13^C-NMR spectral features of **3** resembled those of **10**, except for those of the sugar moiety attached to C-28 of the aglycone. The ^1^H-^1^H COSY, 1D-TOCSY, HSQC, and HSQC-TOCSY spectra indicated that the sugar moiety attached to C-28 of the aglycone consisted of a 2,4-disubstituted β-d-fucopyranosyl unit [Fuc: δ_H_ 5.94 (d, *J* = 8.4 Hz, H-1’’’’ of Fuc); δ_C_ 94.6, 74.4, 76.8, 84.0, 71.6, and 17.1 (C-1’’’’–C-6’’’’ of Fuc)], a 4-substituted α-l-rhamnopyranosyl unit [Rha: δ_H_ 6.34 (br s, H-1’’’’’ of Rha; δ_C_ 101.2, 71.8, 72.4, 83.5, 68.3, and 18.6 (C-1’’’’’–C-6’’’’’ of Rha); ^1^*J*_H-1,C-1_ = 169 Hz)], a 3-substituted β-d-xylopyranosyl unit [Xyl (II): δ_H_ 5.16 (d, *J* = 7.2 Hz, H-1’’’’’’ of Xyl (II); δ_C_ 106.2, 74.8, 88.2, 69.3, and 66.7 (C-1’’’’’’–C-5’’’’’’ of Xyl (II))], a terminal β-d-glucopyranosyl unit [Glc: δ_H_ 5.18 (d, *J* = 7.8 Hz, H-1’’’’’’’ of Glc); δ_C_ 105.5, 75.4, 78.3, 71.5, 78.6, and 62.5 (C-1’’’’’’’–C-6’’’’’’’ of Glc)], and a terminal β-d-quinovopyranosyl unit [Qui: δ_H_ 4.97 (d, *J* = 7.8 Hz, H-1’’’’’’’’ of Qui); δ_C_ 106.7, 75.8, 78.4, 76.6, 73.2, and 18.5 (C-1’’’’’’’’–C-6’’’’’’’’ of Qui)]. HMBC correlations were observed between H-1’’’’’’’ of Glc and C-3’’’’’’ of Xyl (II), H-1’’’’’’ of Xyl (II) and C-4’’’’’ of Rha, H-1’’’’’ of Rha and C-2’’’’ of Fuc, H-1’’’’’’’’ of Qui and C-4’’’’ of Fuc, and between H-1’’’’ of Fuc and C-28 (δ_C_ 176.0) of the aglycone. Therefore, **3** was identified as 3β-[(*O*-β-d-galactopyranosyl-(1→2)-*O*-[β-d-xylopyranosyl-(1→3)]-β-d-glucuronopyranosyl)oxy]-16α-hydroxy-23-oxo-olean-12-en-28-oic acid *O*-β-d-glucopyranosyl-(1→3)-*O*-β-d-xylopyranosyl-(1→4)-*O*-α-l-rhamnopyranosyl-(1→2)-*O*-[β-d-quinovopyranosyl-(1→4)]-β-d-fucopyranosyl ester.

Comparison of the ^1^H- and ^13^C-NMR spectra of **4** (C_80_H_124_O_43_) with those of **3** exhibited considerable structural similarity. However, the molecular formula of **4** contained an additional C_4_H_4_O_2_, and signals arising from two acetyl groups were observed in the ^1^H- and ^13^C-NMR spectra of **4** [δ_H_ 2.04 (s); δ_C_ 170.1 and 20.7; and δ_H_ 1.96 (s); δ_C_ 170.4 and 20.6]. These data suggest that **4** is a diacetylated analog of **3**. In the HMBC spectrum of **4**, long-range correlations were observed from H-3’’’’’’’’ of Qui (δ_H_ 5.60) to an acetyl carbonyl carbon (δ_C_ 170.4), and from H-4’’’’’’’’ of Qui (δ_H_ 5.05) to another acetyl carbonyl carbon (δ_C_ 170.1). Accordingly, **4** was determined to be 3β-[(*O*-β-d-galactopyranosyl-(1→2)-*O*-[β-d-xylopyranosyl-(1→3)]-β-d-glucuronopyranosyl)oxy]-16α-hydroxy-23-oxo-olean-12-en-28-oic acid *O*-β-d-glucopyranosyl-(1→3)-*O*-β-d-xylopyranosyl-(1→4)-*O*-α-l-rhamnopyranosyl-(1→2)-*O*-[3,4-di-*O*-acetyl-β-d-quinovopyranosyl-(1→4)]-β-d-fucopyranosyl ester.

**Figure 2:**
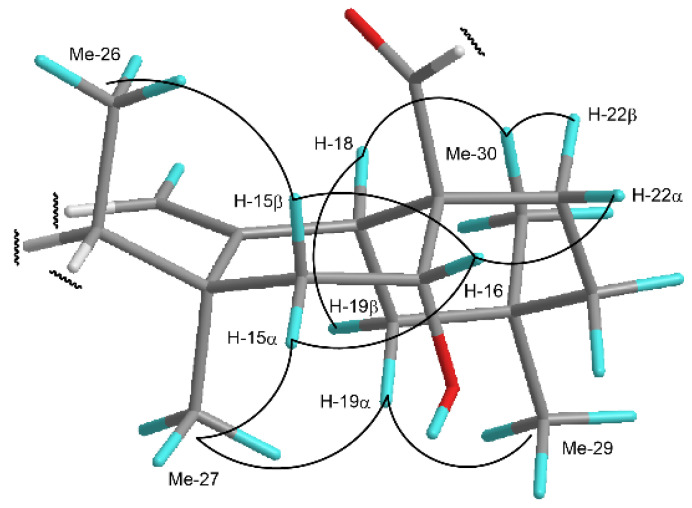
Important NOE correlations observed in the NOESY spectrum of **5**. The C-16α hydroxy configuration was confirmed based on the NOE correlations between H-16 and H-15α/H-15β/H-22α.

The ^1^H- and ^13^C-NMR spectral data of **5** (C_53_H_84_O_25_) suggested that **5** was analogous to **15**, including the sugar moieties attached to C-3 and C-28 of the aglycone. The molecular formula of **5** contained an additional oxygen atom in comparison to that of **15** (C_53_H_84_O_24_). Comparing the ^13^C-NMR spectrum of **5** with that of **15**, the methylene carbon signal (δ_C_ 21.1) attributed to C-16 in **15** was displaced by the oxygenated methine carbon signal (δ_C_ 74.1) in **5**. Furthermore, H-16 (δ_H_ 5.23) showed a spin-coupling correlation with H_2_-15 (δ_H_ 2.48 and 1.69) in the ^1^H-^1^H COSY spectrum of **5**, and long-range correlations were observed between H-18 (δ_H_ 3.47)/H_2_-22 (δ_H_ 2.26 and 2.01) and C-16 in the HMBC spectrum. The above spectral data indicate the presence of a hydroxy group at C-16 in **5**. The configuration of the C-16 hydroxy group was determined to be α based on the nuclear Overhauser effect (NOE) correlations between H-16 and H-15α/H-15β/H-22α in the nuclear Overhauser and exchange spectroscopy (NOESY) spectrum of **5** (Figure 2). Thus, **5** was deduced to be 16α-hydroxy-3β-[(β-d-xylopyranosyl)oxy]olean-12-en-23,28-dioic acid 28-*O*-β-d-glucopyranosyl-(1→2)-*O*-β-d-glucopyranosyl-(1→6)-β-d-glucopyranosyl ester.

Compound **6** (C_65_H_102_O_33_) was shown to be essentially analogous to **16**, including the sugar moieties attached to C-3 and C-28 of the aglycone, based on the ^1^H- and ^13^C-NMR spectral data. However, the molecular formula of **6** contained an additional C_6_H_8_O_4_ compared to that of **16** (C_59_H_94_O_29_), and the ^13^C-NMR spectra of **6** indicated the presence of a six-atom substituent, which was composed of an ester carbonyl carbon (δ_C_ 171.7, C-1’’’’’’), two methylene carbons (δ_C_ 46.6, C-2’’’’’’; δ_C_ 46.3, C-4’’’’’’), a quaternary carbon nearing a hydroxy group (δ_C_ 70.0, C-3’’’’’’), a carbonyl carbon of a carboxy group (δ_C_ 174.7, C-5’’’’’’), and a methyl carbon (δ_C_ 28.2, C-6’’’’’’). The signals in the ^1^H-NMR spectrum that could be assigned to this substituent involved two isolated spin systems consisting of two methyl groups [δ_H_ 3.18 (1H, d, *J* = 15.0 Hz, H-4’’’’’’a) and 3.15 (1H, d, *J* = 15.0 Hz, H-4’’’’’’b); δ_H_: 3.16 (1H, d, *J* = 14.3 Hz, H-2’’’’’’a) and 3.11 (1H, d, *J* = 14.3 Hz, H-2’’’’’’b)] and a deshielded methyl group [δ_H_ 1.75 (3H, s, Me-6’’’’’’)]. These spectroscopic data indicated that the substituent was 3-hydroxy-3-methylglutaryl (HMG). In the HMBC spectrum of **6**, a long-range correlation was observed between H_2_-6’’’’ (δ_H_ 4.92 and 4.68) of Glc (III) and C-1’’’’’’ of the HMG moiety. Accordingly, **6** was identified as 3β-[(β-d-xylopyranosyl)oxy]-olean-12-en-23,28-dioic acid 28-*O*-β-d-glucopyranosyl-(1→2)-*O*-(6-*O*-3-hydroxy-3-methylglutaryl-β-d-glucopyranosyl)-(1→6)-*O*-[β-d-glucopyranosyl-(1→3)]-β-d-glucopyranosyl ester.

Comparison of the ^1^H- and ^13^C-NMR spectra of **7** (C_65_H_102_O_34_) with those of **6** suggested that **7** was closely related to **6**, and that they both shared the same sugar moieties at C-3 and C-28 of the aglycone. The molecular formula of **7** contained an additional oxygen atom compared to that of **6**, and the methylene carbon signal (δ_C_ 23.1) assigned to C-16 in **6** was displaced by the oxygenated methine carbon signal (δ_C_ 74.0) in **7**. Furthermore, H-16 (δ_H_ 5.20) exhibited a spin-coupling correlation with H_2_-15 (δ_H_ 2.38 and 1.65) in the ^1^H-^1^H COSY spectrum of **7**, and long-range correlations were observed between H-18 (δ_H_ 3.46)/H_2_-22 (δ_H_ 2.33 and 2.08) and C-16 in the HMBC spectrum of **7**. Thus, the presence of a hydroxy group at C-16 of the aglycone, as in **5**, was evident. In the NOESY spectrum of **7**, NOE correlations were observed between H-16 and H-15α/H-15β/H-22α. Therefore, the configuration at the C-16 hydroxy group was ascertained to be α. Compound **7** was elucidated as 16α-hydroxy-3β-[(β-d-xylopyranosyl)oxy]olean-12-en-23,28-dioic acid 28-*O*-β- d-glucopyranosyl-(1→2)-*O*-(6-*O*-3-hydroxy-3-methylglutaryl-β-d-glucopyranosyl)-(1→6)-*O*-[β-d-glucopyranosyl-(1→3)]-β-d-glucopyranosyl ester. The configurations of the asymmetric centers of the HMG moiety of **6** and **7** could not be determined presently, owing to insufficient compound quantities.

### 2.2. Cytotoxic Activities of ***1**–**17***

**Figure 3:**
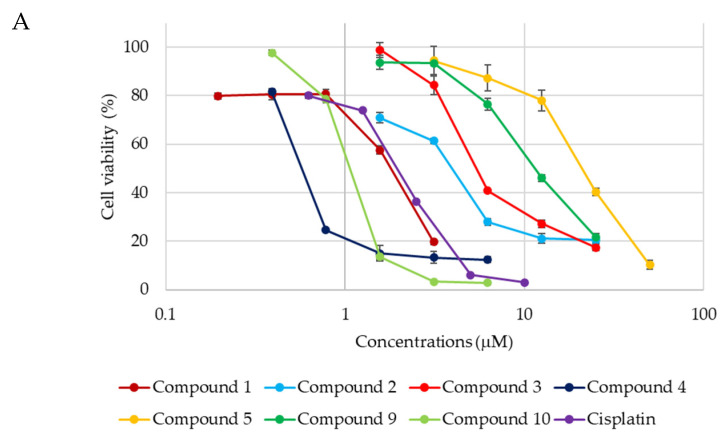

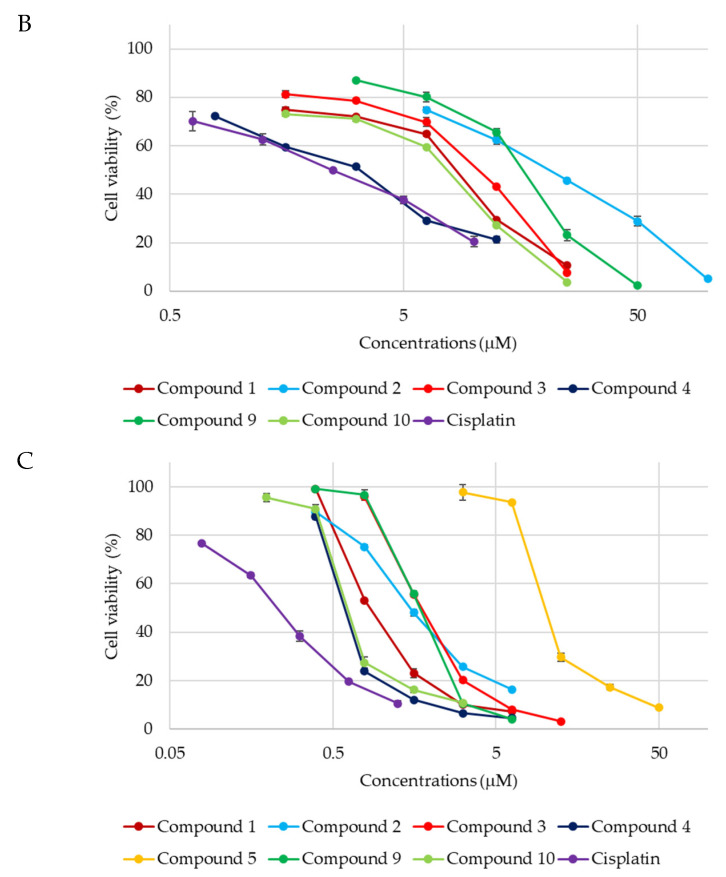
Dose–response curves of **1**–**5**, **9**, **10**, and cisplatin. HL-60 and SBC-3 cells were treated with either **1**–**5**, **9**, **10**, or cisplatin for 72 h (**A**,**C**), and A549 cells were treated with either **1**–**4**, **9**, **10**, or cisplatin for 72 h (**B**). The cell viability was evaluated by the modified MTT assay.

The cytotoxic activities of **1**–**17** against HL-60 cells, A549 cells, and SBC-3 cells were evaluated using the modified 3-(4,5-dimethylthiazol-2-yl)-2,5-diphenyl-2-tetrazolium bromide (MTT) assay (Table 1). As shown in Figure 3, **1**–**5**, **9**, and **10** exerted cytotoxic effects in a dose-dependent manner. The cytotoxicities of **1**, **4**, and **10** toward HL-60 cells and SBC-3 cells were almost as potent as that of cisplatin, which was used as the positive control.

### 2.3. Apotosis-Inducing Activity of ***1***

**Figure 4:**
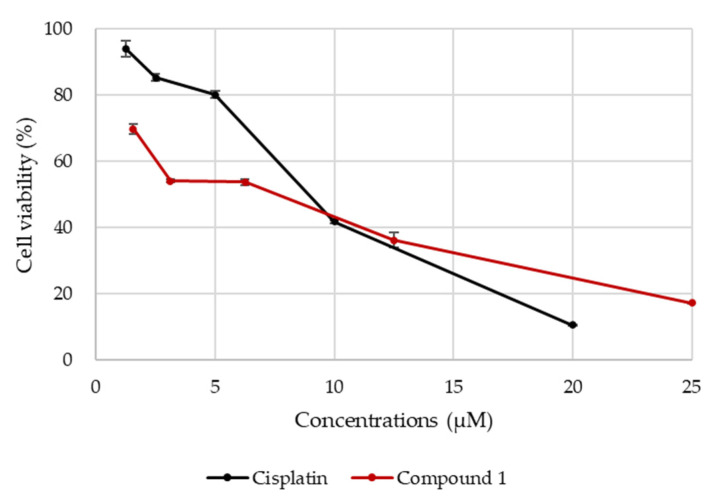
Dose–response curves of **1** and cisplatin. SBC-3 cells were treated with either **1** or cisplatin for 24 h, and the cell viability was evaluated by the modified MTT assay.

Compound **1** exhibited potent cytotoxic activity against SBC-3 cells and was obtained in a good yield. Thus, the apoptosis-inducing activity of **1** in SBC-3 cells was evaluated. Prior to assessing the apoptosis-inducing activity, SBC-3 cells were exposed to either **1** or cisplatin for 24 h to obtain IC_50_ values. The IC_50_ values of **1** and cisplatin were calculated to be 7.3 and 8.6 μM, respectively, based on the dose–response curves (Figure 4). Thus, the apoptosis-inducing activity of **1** was evaluated at 10 μM.

#### 2.3.1. Apoptosis Induced by **1**

**Figure 5:**
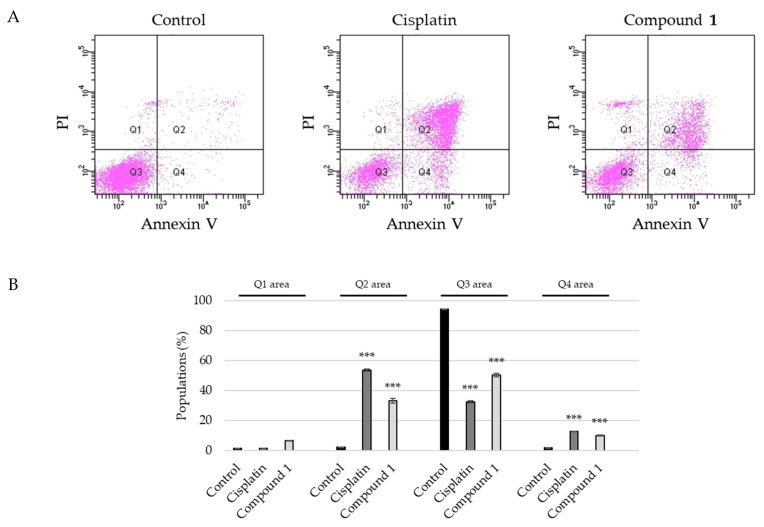
Detection of apoptosis in SBC-3 cells treated with cisplatin or **1**. (**A**) SBC-3 cells treated with either 10 μM of cisplatin or 10 μM of **1**. After 24 h treatment, SBC-3 cells were stained with Annexin V and propidium iodide (PI), and then analyzed using a flow cytometer. (**B**) The bar graph for the percentage of populations of dead cells (Q1 area), late apoptotic cells (Q2 area), live cells (Q3 area), and early apoptotic cells (Q4 area) (*** *p* < 0.001 vs. vehicle control).

After SBC-3 cells were treated with **1** for 24 h, the cells were stained with Annexin V and propidium iodide (PI), and the apoptotic cell ratio was analyzed using flow cytometry. The percentage of early (Q4 area) and late (Q2 area) apoptotic cell populations increased significantly to 10 ± 0.32% and 33 ± 1.5% for **1** compared to 2.0 ± 0.058% and 2.2 ± 0.12%, respectively, for the vehicle control (Figure 5).

#### 2.3.2. Cell Cycle Arrest at the G_2_/M Phase by **1**

**Figure 6:**
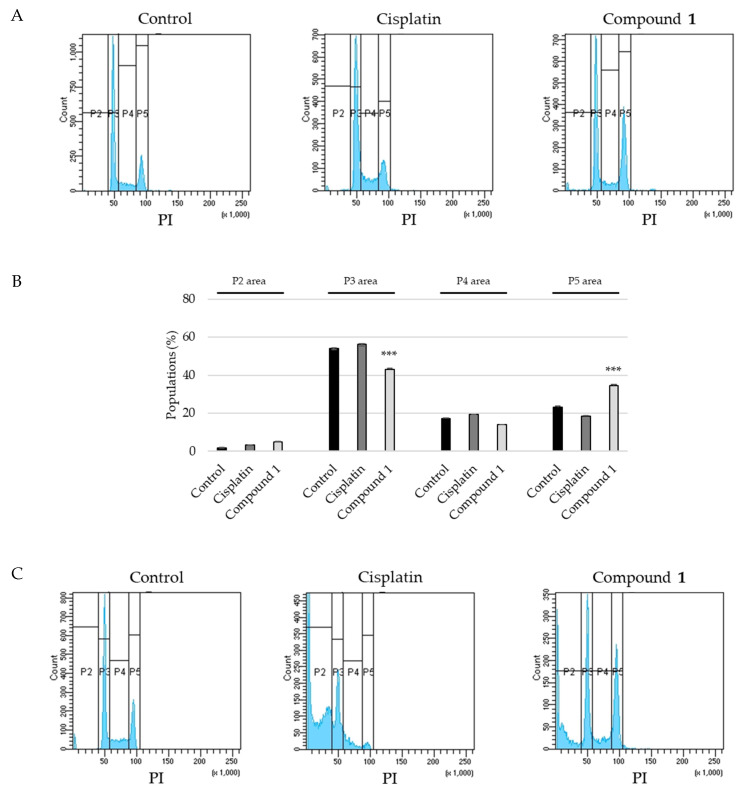

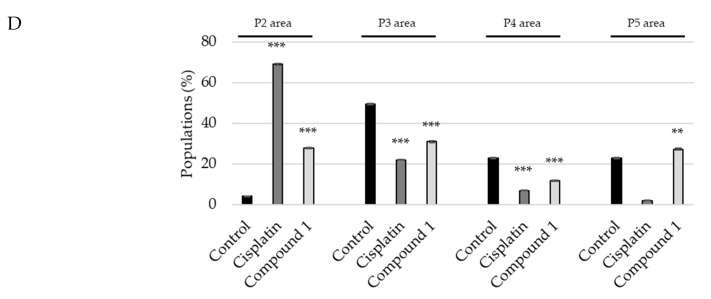
Cell cycle progression of SBC-3 cells treated with cisplatin or **1**. (**A**,**C**) SBC-3 cells were incubated with either 10 μM of cisplatin or 10 μM of **1** for 12 h and 24 h, and the cell cycle distribution was analyzed using a flow cytometer. (**B**,**D**) The cell percentages in the sub-G_1_ (P2 area), G_0_/G_1_ (P3 area), S (P4 area), and G_2_/M (P5 area) phase are shown as the mean ± S.E.M. of three experiments for 12 h and 24 h treatments (*** *p* < 0.001, ** *p* < 0.01 vs. vehicle control).

To determine the cell cycle distribution of SBC-3 cells treated with **1**, flow cytometry analysis was performed using PI staining. After 12 h of treatment with **1**, the cell population in the G_2_/M phase (P5 area) increased to 35 ± 0.45% from 23 ± 0.48%, which was the value obtained after treatment with the vehicle control (Figure 6A,B). Furthermore, the sub-G_1_ phase (P2 area) population of SBC-3 cells treated with **1** for 24 h was 28 ± 0.23%, while that after treatment with the vehicle control was 4.1 ± 0.15% (Figure 6C,D). These data indicated that **1** arrested SBC-3 cell proliferation in the G_2_/M phase and induced apoptotic cell death.

#### 2.3.3. Caspase Activation and PARP Cleavage by **1**

**Figure 7:**
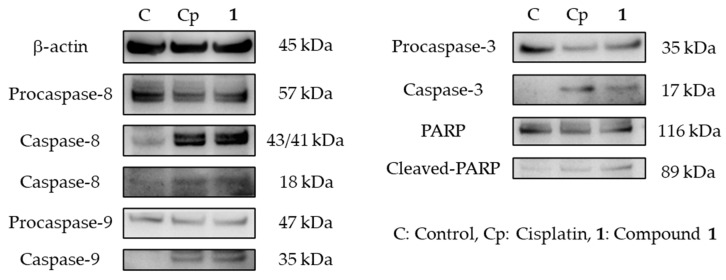
Expression of β-actin, caspase-8, -9, -3, and PARP in SBC-3 cells treated with cisplatin or **1**. SBC-3 cells were incubated with either 10 μM of cisplatin or 10 μM of **1** for 24 h, and the expression of β-actin, caspase-8, -9, -3, and cleaved-PARP was detected using Western blotting analysis.

Caspases, which are cysteine proteases that cleave after aspartic acid residues in a substrate, play an important role in apoptosis and are subclassified into initiator caspases (caspase-8 and -9) and executioner caspases (caspase-3, -6, -7) [25]. During apoptosis, PARP cleavage is a useful hallmark of this type of cell death [26]. To confirm the contribution of caspases to the induction of apoptosis by **1**, Western blotting analysis was performed. After SBC-3 cells were treated with **1** for 24 h, proteins were extracted and subjected to Western blotting analysis. As a result, the activation of caspase-8, -9, and -3, and PARP cleavage were observed (Figure 7).

#### 2.3.4. Mitochondrial Dysfunction Induced by **1**

**Figure 8:**
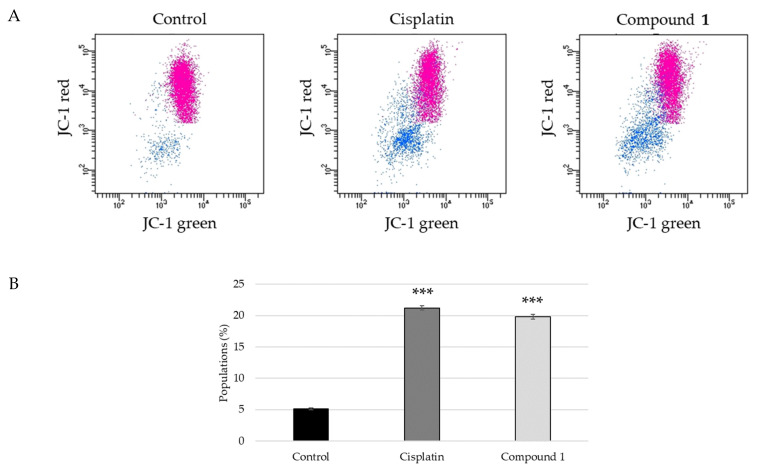
Mitochondrial membrane potential detection in SBC-3 cells treated with cisplatin or **1**. (**A**) SBC-3 cells were treated with either 10 μM of cisplatin or 10 μM of **1** for 24 h, and then stained with JC-1 followed by flow cytometry analysis. (**B**) The percentage of mitochondria membrane potential depolarized cells (blue dots) is exhibited as the mean ± S.E.M. of three experiments (*** *p* < 0.001 vs. vehicle control).

**Figure 9:**
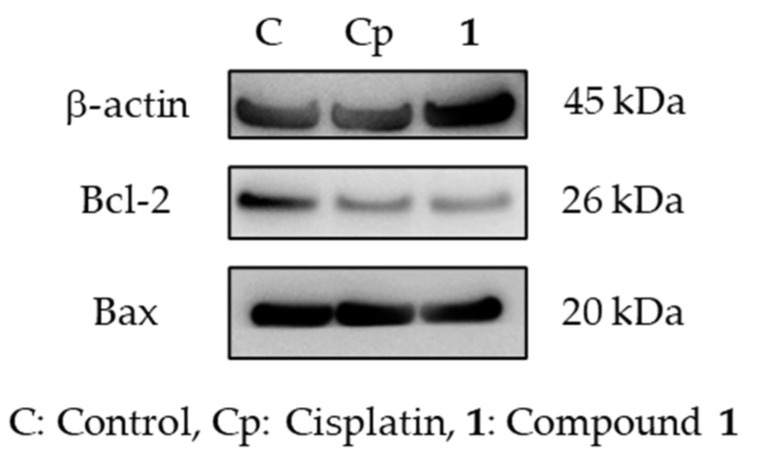
Expression of β-actin, Bcl-2, and Bax in SBC-3 cells treated with cisplatin or **1**. SBC-3 cells were treated with either 10 μM of cisplatin or 10 μM of **1** for 24 h, and the expression of β-actin, Bcl-2, and Bax was evaluated using Western blotting analysis.

There are two major apoptosis-inducing pathways: intrinsic and extrinsic. The intrinsic pathway is also known as the mitochondrial pathway and participates in the activation of caspase-9 [27]. Because the activation of caspase-9 in SBC-3 cells treated with **1** was confirmed, the mitochondrial membrane potential was evaluated employing the JC-1 assay. When cells are stained with JC-1 dye, at low mitochondrial membrane potentials, the concentration of JC-1 is low and it exists predominantly as a monomer, exhibiting green fluorescence with emission; at high mitochondrial membrane potentials, the dye accumulates in the mitochondria and the dye aggregates yield a red to red-colored emission. SBC-3 cells were treated with **1** for 24 h, and then analyzed using a flow cytometer. As shown in Figure 8, the population of the mitochondrial membrane potential depolarized cells significantly increased compared to that observed with the vehicle control. Additionally, the expression of Bcl-2 and Bax was evaluated employing Western blot analysis. Bcl-2 and Bax belong to the Bcl-2 family, which regulates the intrinsic apoptotic pathway. Bcl-2 is an anti-apoptotic protein, whereas Bax acts as a pro-apoptotic effector [28]. In SBC-3 cells treated with **1**, the expression level of Blc-2 was remarkably diminished, and the ratio of Bcl-2/Bax was lower than that of the vehicle control (Figure 9). These data suggest that **1** causes mitochondrial dysfunction in SBC-3 cells.

#### 2.3.5. ROS Generation by **1**

**Figure 10:**
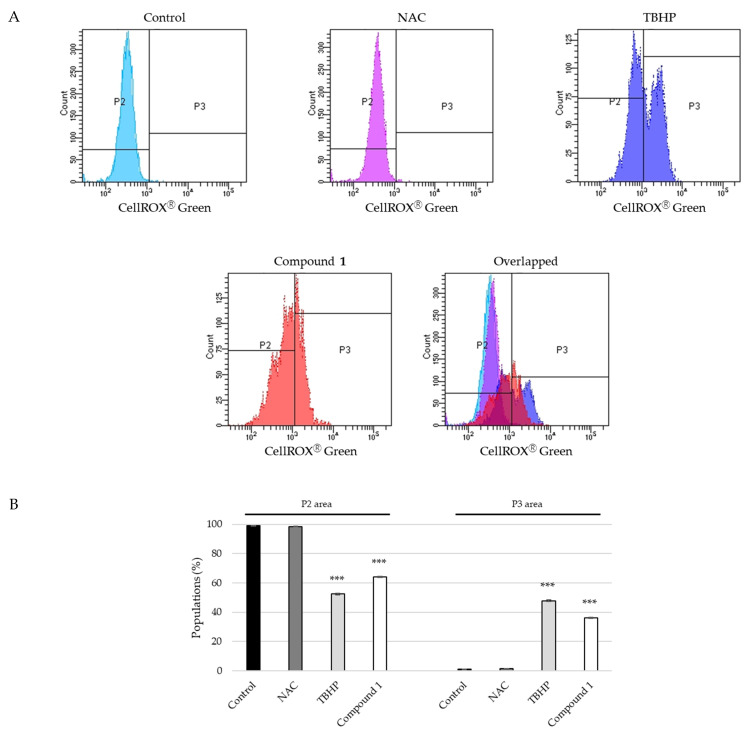
ROS production in SBC-3 cells treated with either *N*-acetylcysteine (NAC), *tert*-butyl hydroperoxide (TBHP) or **1**. (**A**) SBC-3 cells were incubated with either 2.5 mM of NAC, 100 μM of TBHP, or 10 μM of **1** for 24 h. ROS levels were analyzed using a flow cytometer. (**B**) The percentage of cells in the P2 area and P3 area are shown as the mean ± S.E.M. of three experiments (*** *p* < 0.001 vs. vehicle control).

Reactive oxygen species (ROS) exhibit beneficial or harmful effects on cells and tissues. ROS have been reported to be associated with intrinsic and extrinsic apoptotic pathways [29,30]. SBC-3 cells were incubated with either 2.5 mM of *N*-acetylcysteine (NAC; negative control), 100 μM of *tert*-butyl hydroperoxide (TBHP; positive control), or **1** for 24 h, and then analyzed using a flow cytometer. As depicted in Figure 10, the cell fluorescence intensities of the control- and NAC-treated groups were weak, while the peaks of the cell populations of the TBHP- or **1**-treated groups moved to the right side. Based on these results it can be concluded that ROS generation occurred in SBC-3 cells treated with **1**.

### 2.4. Mitophagy Occurrence

**Figure 11:**
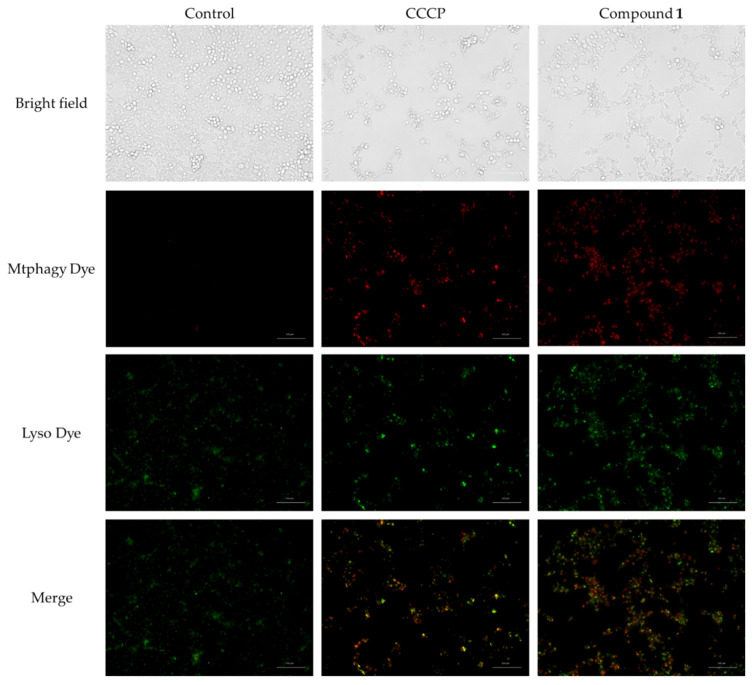
Mitophagy occurrence in SBC-3 cells treated with carbonyl cyanide *m*-chlorophenylhydrazone (CCCP) or **1**. SBC-3 cells were treated with either 7.5 μM of CCCP or 10 μM of **1** for 24 h and observed by a fluorescence microscopy. The scale bars indicate 100 μm.

Mitophagy is defined as mitochondria-selective autophagy and plays a role in the elimination of defective mitochondria [31]. Several reports have suggested that depolarization of the mitochondrial membrane potential and ROS production are involved in mitophagy [32,33,34]. As **1** induced depolarization of the mitochondrial membrane potential and ROS generation, we investigated whether mitophagy occurred in SBC-3 cells. SBC-3 cells were treated with **1** or 7.5 μM of carbonyl cyanide *m*-chlorophenylhydrazone (CCCP; positive control) for 24 h, and then stained with Mtphagy Dye and Lyso Dye. Mtphagy Dye is a fluorescent dye that binds mitochondria and emits red fluorescence, the intensity of which increases under acidic conditions when the mitochondria are fused with lysosomes. Lyso Dye stains lysosomes and emits green fluorescence. SBC-3 cells incubated with **1** emitted intense red fluorescence and exhibited co-localization with lysosomes, while those treated with the control did not (Figure 11). These findings suggest that mitophagy had occurred in SBC-3 cells.

## 3. Materials and Methods

### 3.1. General Experimental Procedures

Optical rotations were measured using a P-1030 automatic digital polarimeter (JASCO, Tokyo, Japan). IR spectral data were obtained on a Fourier-transform infrared (FT-IR) 620 spectrometer (JASCO). NMR spectral data were collected using a Bruker AVIIIHD-600 (600 MHz for ^1^H-NMR; 150 MHz for ^13^C-NMR) spectrometer (Karlsruhe, Germany) and JNM-ECZ600R/M1 (600 MHz for ^1^H-NMR; 150 MHz for ^13^C-NMR) spectrometer (JEOL, Tokyo, Japan) at 300 K. Chemical shifts are recorded as δ values and referenced to tetramethylsilane (TMS) as the internal standard. HR-ESI-TOF-MS data were collected by a Waters Micromass LCT mass spectrometer (Milford, MA, USA). Diaion HP-20 porous polymer polystyrene resin (Mitsubishi-Chemical, Tokyo, Japan), silica gel Chromatorex BW-300 (Fuji-Silysia Chemical, Aichi, Japan), and ODS silica gel COSMOSIL 75C_18_-OPN (Nacalai-Tesque, Kyoto, Japan) were adopted for CC. Thin layer chromatography (TLC) analysis was conducted using precoated silica gel 60F_254_ or RP18 F_254_S plates (0.25 mm thick; Merck, Darmstadt, Germany). The sample spots were visualized by spraying the TLC plates with H_2_SO_4_/H_2_O (1:9), and then heating. The preparative HPLC system consisted of an LC-20AD pump (Shimadzu, Kyoto, Japan), a RID-10A detector (Shimadzu), a Rheodyne injection port (Thermo Fisher Scientific, Waltham, MA, USA), and a TSKgel ODS-100Z column (10 mm i.d. × 250 mm, 5 μm; Tosoh, Tokyo, Japan). The purities of the isolated compounds were confirmed by NMR spectra and TLC. The following materials and reagents were recruited for the cell culture and cytotoxic assays: SH-1300 Lab microplate reader (CORONA ELECTRIC, Ibaraki, Japan); 96-well flat-bottomed and 6-well flat-bottomed plates (Iwaki Glass, Chiba, Japan), Countess II FL automated cell counter (Thermo Fisher Scientific); MCO-170AIC-PJ CO_2_ incubator (PHC, Tokyo, Japan); 0.25% trypsin-ethylenediaminetetraacetic acid (EDTA) solution, fetal-bovine serum (FBS), RPMI-1640 medium, minimum-essential medium (MEM), cisplatin, and MTT (Sigma, St. Louis, MO, USA); penicillin G sodium salt and streptomycin sulfate, and TrypLE Select (1×) (Gibco, Gland Island, NY, USA); paraformaldehyde and phosphate-buffered saline (PBS) (FUJIFILM Wako Pure Chemical, Osaka, Japan); HL-60 cells (JCRB0085), A549 cells (JCRB0076), and SBC-3 cells (JCRB0818) (Human Science Research Resource Bank, Osaka, Japan).

### 3.2. Plant Material

*S. officinalis* seeds were purchased from Richters Herbs (Goodwood, Ontario, Canada) in 2010. A voucher specimen was maintained at the herbarium of the Tokyo University of Pharmacy and Life Sciences (KS-2010-003).

### 3.3. Extraction and Isolation

The seeds of *S. officinalis* (1.0 kg) were extracted with MeOH (2 L × 2 times, 60 °C), and then the solvent was removed under reduced pressure using an evaporator. The MeOH extract (50 g) was loaded on Diaion HP-20 column and successively eluted with MeOH/H_2_O (1:4; 2 L), EtOH (3 L), and EtOAc (3 L). The EtOH eluted fraction (10 g) was subjected to silica gel CC eluted with a stepwise gradient mixture of CHCl_3_/MeOH (9:1; 4:1; 2:1) to obtain four fractions [Fractions (Frs.) A–D]. Fr. A was divided by ODS silica gel CC eluted with MeCN/H_2_O (2:5) and MeOH/H_2_O (3:2), and preparative ODS HPLC using MeCN/H_2_O (7:13) to afford **13** (3.0 mg) and **15** (26 mg). Fr. B was subjected to silica gel CC eluted with CHCl_3_/MeOH/H_2_O (7:4:1), ODS silica gel CC eluted with MeOH/H_2_O (7:3) and MeCN/H_2_O (7:13; 1:2; 2:5), and preparative ODS HPLC using MeOH/H_2_O (7:3; 3:7) and MeCN/H_2_O (3:7) to yield **5** (7.1 mg), **6** (2.5 mg), and **12** (4.1 mg). Fr. C was separated by silica gel CC eluted with CHCl_3_/MeOH/H_2_O (7:4:1), ODS silica gel CC eluted with MeOH/H_2_O (13:7) and MeCN/H_2_O (2:5; 3:7), and preparative ODS HPLC using MeOH/H_2_O (13:7) and MeCN/H_2_O (3:7) to furnish **7** (3.2 mg), **10** (84 mg), **11** (4.9 mg), and **14** (2.0 mg). Fr. D was chromatographed on silica gel eluted with CHCl_3_/MeOH/H_2_O (7:4:1), and ODS silica gel eluted with MeOH/H_2_O (3:2; 13:7) and MeCN/H_2_O (7:13; 3:7; 1:3) to obtain **1** (184 mg), **2** (38 mg), **3** (4.6 mg), **4** (15 mg), **8** (19 mg), **9** (49 mg), **16** (23 mg), and **17** (55 mg).

### 3.4. Structural Determination

Compound **1**: Amorphous solid; [α]_D_^25^ –4.9 (*c* = 0.10, MeOH); IR (film) ν_max_: 3398 (OH), 2929 (CH), 1730 (C = O) cm^−1^; HR-ESI-TOF-MS *m/z*: 1711.7161 [M + Na]^+^ (calculated for C_76_H_120_NaO_41_: 1711.7203). ^1^H-NMR spectral data for the aglycone moiety (600 MHz, C_5_D_5_N): δ_H_ 9.91 (1H, s, H-23), 5.50 (1H, m, H-12), 5.11 (1H, br s, H-16), 4.06 (1H, m, H-3), 1.73 (3H, s, Me-27), 1.46 (3H, s, Me-24), 1.10 (3H, s, Me-26), 0.93 (3H, s, Me-30), 0.92 (3H, s, Me-29), 0.84 (3H, s, Me-25). ^1^H-NMR spectral data for the sugar moiety, see Table 2. ^13^C-NMR spectral data, see Table 3. NMR spectral data, see Supplementary Materials.

Compound **2**: Amorphous solid; [α]_D_^25^ –16.7 (*c* = 0.05, MeOH); IR (film) ν_max_: 3397 (OH), 2933 (CH), 1730 (C = O) cm^−1^; HR-ESI-TOF-MS *m/z*: 1753.7300 [M + Na]^+^ (calculated for C_78_H_122_NaO_42_: 1753.7308). ^1^H-NMR spectral data for the aglycone moiety (600 MHz, C_5_D_5_N): δ_H_ 9.92 (1H, s, H-23), 5.53 (1H, m, H-12), 5.12 (1H, br s, H-16), 4.09 (1H, m, H-3), 1.72 (3H, s, Me-27), 1.49 (3H, s, Me-24), 1.17 (3H, s, Me-26), 0.93 (3H, s, Me-30), 0.90 (3H, s, Me-29), 0.89 (3H, s, Me-25). ^1^H-NMR spectral data for the sugar and acyl moieties, see Table 2. ^13^C-NMR spectral data, see Table 3. NMR spectral data, see Supplementary Materials.

Compound **3**: Amorphous solid; [α]_D_^25^ –5.8 (*c* = 0.05, MeOH); IR (film) ν_max_: 3398 (OH), 2925 (CH), 1730 (C = O) cm^−1^; HR-ESI-TOF-MS *m/z*: 1711.7191 [M + Na]^+^ (calculated for C_76_H_120_NaO_41_: 1711.7203). ^1^H-NMR spectral data for the aglycone moiety (600 MHz, C_5_D_5_N): δ_H_ 9.91 (1H, s, H-23), 5.53 (1H, m, H-12), 5.15 (1H, br s, H-16), 4.05 (1H, m, H-3), 1.73 (3H, s, Me-27), 1.44 (3H, s, Me-24), 1.03 (3H, s, Me-26), 0.96 (3H, s, Me-30), 0.93 (3H, s, Me-29), 0.81 (3H, s, Me-25). ^1^H-NMR spectral data for the sugar moiety, see Table 2. ^13^C-NMR spectral data, see Table 3. NMR spectral data, see Supplementary Materials.

Compound **4**: Amorphous solid; [α]_D_^25^ + 3.6 (*c* = 0.05, MeOH); IR (film) ν_max_: 3398 (OH), 2929 (CH), 1726 (C = O) cm^−1^; HR-ESI-TOF-MS *m/z*: 1771.7439 [M–H]^−^ (calculated for C_80_H_123_O_43_: 1771.7438). ^1^H-NMR spectral data for the aglycone moiety (600 MHz, C_5_D_5_N): δ_H_ 9.91 (1H, s, H-23), 5.53 (1H, m, H-12), 5.12 (1H, br s, H-16), 4.05 (1H, m, H-3), 1.74 (3H, s, Me-26), 1.73 (3H, s, Me-27), 1.44 (3H, s, Me-24), 0.97 (3H, s, Me-30), 0.93 (3H, s, Me-29), 0.81 (3H, s, Me-25). ^1^H-NMR spectral data for the sugar and acyl moieties, see Table 2. ^13^C-NMR spectral data, see Table 3. NMR spectral data, see Supplementary Materials.

Compound **5**: Amorphous solid; [α]_D_^25^ –76.5 (*c* = 0.05, MeOH); IR (film) ν_max_: 3358 (OH), 2925 (CH), 1712 (C = O) cm^−1^; HR-ESI-TOF-MS *m/z*: 1143.5189 [M + Na]^+^ (calculated for C_53_H_84_NaO_25_: 1143.5199). ^1^H-NMR spectral data for the aglycone moiety (600 MHz, C_5_D_5_N): δ_H_ 5.59 (1H, dd, *J* = 3.4, 3.4 Hz, H-12), 5.23 (1H, br s, H-16), 4.64 (1H, dd, *J* = 12.0, 4.4 Hz, H-3), 1.76 (3H, s, Me-27), 1.56 (3H, s, Me-24), 1.11 (3H, s, Me-26), 0.99 (3H, s, Me-30), 0.98 (3H, s, Me-25), 0.92 (3H, s, Me-29). ^1^H-NMR spectral data for the sugar moiety, see Table 2. ^13^C-NMR spectral data, see Table 3. NMR spectral data, see Supplementary Materials.

Compound **6**: Amorphous solid; [α]_D_^25^ + 8.5 (*c* = 0.05, MeOH); IR (film) ν_max_: 3377 (OH), 2925 (CH), 1717 (C = O) cm^−1^; HR-ESI-TOF-MS *m/z*: 1433.6193 [M + Na]^+^ (calculated for C_65_H_102_NaO_33_: 1433.6201). ^1^H-NMR spectral data for the aglycone moiety (600 MHz, C_5_D_5_N): δ_H_ 5.39 (1H, dd, *J* = 3.5, 3.5 Hz, H-12), 4.60 (1H, dd, *J* = 11.8, 4.3 Hz, H-3), 1.55 (3H, s, Me-24), 1.17 (3H, s, Me-27), 1.04 (3H, s, Me-26), 0.95 (3H, s, Me-25), 0.91 (3H, s, Me-30), 0.85 (3H, s, Me-29). ^1^H-NMR spectral data for the sugar and acyl moieties, see Table 2. ^13^C-NMR spectral data, see Table 3. NMR spectral data, see Supplementary Materials.

Compound **7**: Amorphous solid; [α]_D_^25^ + 4.4 (*c* = 0.05, MeOH); IR (film) ν_max_: 3377 (OH), 2925 (CH), 1730 (C = O) cm^−1^; HR-ESI-TOF-MS *m/z*: 1449.6165 [M + Na]^+^ (calculated for C_65_H_102_NaO_34_: 1449.6150). ^1^H-NMR spectral data for the aglycone moiety (600 MHz, C_5_D_5_N): δ_H_ 5.57 (1H, m, H-12), 5.20 (1H, br s, H-16), 4.64 (1H, dd, *J* = 11.9, 4.4 Hz, H-3), 1.75 (3H, s, Me-27), 1.56 (3H, s, Me-24), 1.08 (3H, s, Me-26), 1.05 (3H, s, Me-30), 0.99 (3H, s, Me-25), 0.93 (3H, s, Me-29). ^1^H-NMR spectral data for the sugar and acyl moieties, see Table 2. ^13^C-NMR spectral data, see Table 3. NMR spectral data, see Supplementary Materials.

Alkaline treatment of **1** followed acid hydrolysis: Compound **1** (11 mg) was treated with 1 M KOH in MeOH at 80 °C for 90 min. The reaction solution was neutralized by passing through an Amberlite 120B (Organo, Tokyo, Japan) column eluted with EtOH, and was separated by ODS silica gel CC eluted with MeCN/H_2_O (2:3) to obtain **8** (5.8 mg) and a residue. The residue was dealt with 1 M HCl (dioxane/H_2_O, 1:1) at 95 °C for 2 h. The reaction mixture was neutralized by passing through an Amberlite IRA96SB (Organo) column eluted with EtOH to afford a sugar fraction. The sugar fraction was analyzed by HPLC under the following conditions: solvent, MeCN/H_2_O (17:3); flow rate, 0.5 mL/min; column temperature, 40 °C; pump, Tosoh DP-8020; column, Capcell Pak NH_2_ (4.6 mm i.d. × 250 mm, 5 μm; Shiseido, Tokyo, Japan); detector, Shodex OR2 (Showa-Denko, Tokyo, Japan). d-fucose, d-quinovose, d-xylose, d-glucose, and l-rhamnose were identified by comparing their retention times (*t*_R_) and optical rotations with those of authentic samples: d-fucose (12.69, positive optical rotation), d-quinovose (13.06, positive optical rotation), d-xylose (14.37, positive optical rotation), d-glucose (19.66, positive optical rotation), and l-rhamnose (12.17, negative optical rotation).

### 3.5. Cell Culture and Cytotoxic Activity Assay

HL-60 cells were maintained in RPMI-1640 medium, and A549 cells and SBC-3 cells in MEM containing 10% heat-inactivated FBS supplemented with l-glutamine, 100 unit/mL penicillin G sodium salt, and 100 μg/mL streptomycin sulfate. The cells were incubated at 37 °C in a 5% CO_2_/air atmosphere. HL-60 cells (4 × 10^4^ cells/mL), A549 cells (1 × 10^4^ cells/mL), and SBC-3 cells (2 × 10^4^ cells/mL) were seeded in a 96-well flat-bottomed plate. After pre-incubation for 24 h, 4 μL of EtOH/H_2_O (1:1) solution containing each test sample was added and incubated for 72 h; 4 μL of EtOH/H_2_O (1:1) solution was added to the control cells. Cell viability was evaluated using the modified MTT reduction assay method. After terminating the cell culture, 10 μL of MTT solution (5 mg/mL in PBS) was added to each well, and the plate was further incubated. After 4 h of incubation, MTT formazan was dissolved in dimethyl sulfoxide (DMSO) and the absorbance was measured at 405 nm. A dose–response curve was plotted for **1**–**5**, **9**, and **10**, which inhibited cell growth by more than 50% at sample concentrations of 50 μM, and the concentrations at which 50% inhibition (IC_50_) of cell growth occurred were calculated. The cell growth inhibition of SBC-3 cells (5 × 10^4^ cells/mL) treated with **1** for 24 h was evaluated using the same method as above.

### 3.6. Apoptosis Induction Assay

SBC-3 cells (5 × 10^5^ cells/mL) were harvested in a 6-well flat-bottomed plate and pre-incubated for 24 h. Then, SBC-3 cells were treated with either EtOH/H_2_O (1:1) (control), 10 μM of cisplatin, or 10 μM of **1**. After 24 h of incubation, SBC-3 cells were detached using TrypLE Select and stained with 1 × Annexin V binding buffer containing Annexin V-FITC and PI at 28 °C for 15 min according to the manufacturer’s protocol (Nacalai Tesque). Flow cytometry analysis was conducted using a BD FACSCelesta flow cytometer (BD Biosciences, Franklin Lakes, NJ, USA).

### 3.7. Cell Cycle Distrubution Analysis

SBC-3 cells (5 × 10^5^ cells/mL) were cultured in a 6-well flat-bottomed plate and treated with either EtOH/H_2_O (1:1) (control), 10 μM of cisplatin, or 10 μM of **1**. After 12 h or 24 h of incubation, SBC-3 cells were detached using TrypLE Select and fixed with EtOH/PBS (7:3) at −20 °C overnight. After removal of EtOH/PBS (7:3), the cells were stained with FxCycle PI/RNase Staining Solution (Thermo Fisher Scientific) at 28 °C for 15 min. Cell cycle distribution was analyzed using a BD FACSCelesta flow cytometer.

### 3.8. Western Blotting Analysis

SBC-3 cells (5 × 10^5^ cells/mL) were seeded into a 6-well flat-bottomed plate and treated with either EtOH/H_2_O (1:1) (control), 10 μM of cisplatin, or 10 μM of **1**. After 24 h of incubation, SBC-3 cells were detached using TrypLE Select. The SBC-3 cell proteins were extracted on ice for 30 min using RIPA buffer (FUJIFILM Wako Pure Chemical) containing Pierce Protease and Phosphatase inhibitor Mini Tablet (Thermo Fisher Scientific). Then, SBC-3 cells were centrifuged at 10,000× *g* for 30 min, and the supernatant protein solution was collected. Protein quantification was performed using the TaKaRa BCA Protein Assay Kit (Takara Bio, Shiga, Japan). Then, sample protein solutions were loaded on NuPAGE 4–12% Bis-Tris Gel (Thermo Fisher Scientific), and electrophoresis was performed using a WSE-1165 RapidasMinislab electrophoresis tank (ATTO, Tokyo, Japan). Proteins were transferred from the gel to a polyvinylidene difluoride (PVDF) membrane using a Protein Transfer Kit for Semidry (COSMO BIO, Tokyo, Japan) and a WSE-4025HorizeBLOT 2M (ATTO). The PVDF membrane was then blocked using a Blocking One solution (Nacalai Tesque) at 28 °C. After 30 min, the PVDF membrane was reacted with the following primary antibodies dissolved in Can Get Signal Immunoreaction Enhancer Solution 1 (TOYOBO, Osaka, Japan) at 4°C overnight: β-actin (8H10D10 Mouse mAb, #3700, 1:1000; Cell Signaling Technology, Danvers, MA, USA), Caspase-3 (3G2 Mouse mAb, #9668, 1:1000; Cell Signaling Technology), Caspase-8 (1C12 Mouse mAb, #9746, 1:1000; Cell Signaling Technology), Caspase-9 (C9 Mouse mAb, #9508, 1:2000; Cell Signaling Technology), PARP (46D11 Rabbit mAb, #9532, 1:1000; Cell Signaling Technology), Bax (2D2 Mouse mAb, #89477, 1:1000; Cell Signaling Technology), and Bcl-2 (124 Mouse mAb, #15071, 1:1000, Cell Signaling Technology). The PVDF membrane was rinsed with TBS-T buffer (1 ×), and treated with secondary antibodies (Anti-mouse IgG, HRP-linked Antibody, #7076, 1:10,000; Cell Signaling Technology or Anti-rabbit IgG, HRP-linked Antibody, #7074, 1:10,000; Cell Signaling Technology) dissolved in Can Get Signal Immunoreaction Enhancer Solution 2 (TOYOBO) at 28 °C for 1 h. Finally, the PVDF membrane was reacted with ECL Prime Western Blotting Detection Reagents (GE Healthcare, Boston, MA, USA), and detected using the LAS-3000 luminescent image analyzer (FUJIFILM, Tokyo, Japan).

### 3.9. Detection of Mitochondrial Membrane Potential

Mitochondrial membrane potential was assayed using the JC-1 MitoMP Detection Kit (DOJINDO, Kumamoto, Japan). SBC-3 cells (5 × 10^5^ cells/mL) were seeded into a 6-well flat-bottomed plate. SBC-3 cells were treated with either EtOH/H_2_O (1:1) (control), 10 μM of cisplatin, or 10 μM of **1**. After 24 h of incubation, SBC-3 cells were detached using TrypLE Select and stained with JC-1 dye at 37 °C for 45 min according to the manufacturer’s protocol. Flow cytometry analysis was performed using a BD FACSCelesta flow cytometer.

### 3.10. Measurment of ROS Level

The ROS generation assay was performed using the CellROX Green Flow Cytometer Assay Kit (Thermo Fisher Scientific). SBC-3 cells (5 × 10^5^ cells/mL) were cultured in a 6-well flat-bottomed plate and preincubated for 24 h. Then, SBC-3 cells were treated with either EtOH/H_2_O (1:1) (control), 2.5 mM of NAC, 100 μM of TBHP, or 10 μM of **1** for 24 h. SBC-3 cells were detached using TrypLE Select and stained with CellROX Green reagent at 37 °C for 1 h according to the manufacturer’s protocol. ROS levels were measured using a BD FACSCelesta flow cytometer.

### 3.11. Mitophagy Detection

Mitophagy detection was performed using the Mitophagy Detection Kit (DOJINDO). SBC-3 cells (5 × 10^5^ cells/mL) were seeded into a 6-well flat-bottomed plate. After pre-incubation, SBC-3 cells were washed with Hank’s balanced salt solution (HBSS; 1x, Gibco) and treated with 100 nM Mtphagy Dye at 37 °C for 30 min. Then, SBC-3 cells were rinsed with HBSS and treated with either EtOH/H_2_O (1:1) (control), 7.5 μM of CCCP, or 10 μM of **1** for 24 h. After the supernatant was removed and SBC-3 cells were washed with HBSS, the cells were incubated with 1 μM Lyso Dye at 37 °C for 30 min and observed using a BZ-X710 All-in-One Fluorescence Microscope (KEYENCE, Osaka, Japan).

### 3.12. Statistical Analysis

Statistical analysis was conducted using a one-way analysis of variance (ANOVA) followed by Dunnett’s test. A probability (*p*) value of less than 0.001 or 0.01 was distinguished to represent a statistically significant difference.

## 4. Conclusions

In this study, the chemical constituents of *S. officinalis* seeds were investigated, and 17 oleanane-type triterpene glycosides (**1**–**17**), including seven (**1**–**7**) previously unreported compounds, were isolated and identified. Compounds **1**–**5**, **9**, and **10** showed cytotoxicity against HL-60 cells, A549 cells, and SBC-3 cells with IC_50_ values in the range of 0.57–21 μM. The cytotoxicities of **1**, **4**, and **10** toward HL-60 cells and SBC-3 cells were almost as potent as that of cisplatin. Compound **1**, a bisdesmosidic triterpene glycoside obtained in good yield, arrested the cell cycle of SBC-3 cells at the G_2_/M phase, and induced apoptosis through an intrinsic pathway; activation of caspase-8, -9, and -3, cleavage of PARP, reduction in the Bcl-2/Bax ratio, depolarization of mitochondrial membrane potential, and ROS generation were observed after treatment of SBC-3 cells with **1**. As a result of mitochondrial dysfunction induced by **1**, mitochondrial selective autophagy, termed mitophagy, occurred in SBC-3 cells. Compound **1** is a potential hit compound for the development of drugs against small-cell lung cancer.

## Figures and Tables

**Table 1:** Cytotoxic activities of **1**–**17** against HL-60 cells, A549 cells, and SBC-3 cells ^(1)^.

Compounds	HL-60 Cells			A549 Cells			SBC-3 Cells		
	IC_50_ (μM)			IC_50_ (μM)			IC_50_ (μM)		
**1**	1.8	±	0.038	8.4	±	0.055	0.84	±	0.012
**2**	4.0	±	0.039	21	±	0.17	1.5	±	0.052
**3**	5.4	±	0.084	10	±	0.10	1.7	±	0.015
**4**	0.57	±	0.0030	3.3	±	0.21	0.59	±	0.0015
**5**	21	±	0.26		>50		10	±	0.15
**6**		>50			>50			>50	
**7**		>50			>50			>50	
**8**		>50			>50			>50	
**9**	11	±	0.27	16	±	0.020	1.7	±	0.023
**10**	1.1	±	0.010	7.7	±	0.062	0.61	±	0.0072
**11**		>50			>50			>50	
**12**		>50			>50			>50	
**13**		>50			>50			>50	
**14**		>50			>50			>50	
**15**		>50			>50			>50	
**16**		>50			>50			>50	
**17**		>50			>50			>50	
Cisplatin	2.0	±	0.019	2.5	±	0.079	0.23	±	0.0049

^(1)^ Data are represented as the mean value ± S.E.M. of the three experiments performed in triplicate.

**Table 2:** ^1^H-NMR spectral data for the sugar and acyl moieties of **1**–**7**.

**1**					**2**				
Positions		δ_H_		*J* (Hz)	Positions		δ_H_		*J* (Hz)
GlcUA					GlcUA				
1’		4.89	d	7.8	1’		4.89	d	7.8
2’		4.34	dd	8.3, 7.8	2’		4.33	dd	8.4, 7.8
3’		4.24	dd	9.1, 8.3	3’		4.22	dd	9.0, 8.4
4’		4.45	dd	9.4, 9.1	4’		4.44	dd	9.6, 9.0
5’		4.50	d	9.4	5’		4.49	d	9.6
Gal					Gal				
1’’		5.54	d	7.7	1’’		5.53	*	
2’’		4.46	dd	8.8, 7.7	2’’		4.44	dd	9.0, 7.8
3’’		4.16	dd	8.8, 3.2	3’’		4.15	dd	9.0, 3.6
4’’		4.58	br d	3.2	4’’		4.56	br d	3.6
5’’		4.02	m		5’’		4.00	m	
6’’	a	4.51	m		6’’	a	4.50	m	
	b	4.42	m			b	4.41	dd	10.8, 6.0
Xyl (I)					Xyl (I)				
1’’’		5.31	d	7.7	1’’’		5.30	d	7.8
2’’’		3.96	dd	9.1, 7.7	2’’’		3.96	dd	8.4, 7.8
3’’’		4.13	m		3’’’		4.11	dd	8.4, 7.8
4’’’		4.12	m		4’’’		4.12	m	
5’’’	a	4.23	dd	11.7, 5.3	5’’’	a	4.24	m	
	b	3.65	m			b	3.65	dd	11.2, 9.3
Fuc					Fuc				
1’’’’		5.88	d	8.2	1’’’’		5.89	d	8.4
2’’’’		4.45	dd	9.1, 8.2	2’’’’		4.61	dd	9.0, 8.4
3’’’’		4.14	dd	9.1, 3.1	3’’’’		4.21	m	
4’’’’		3.98	br d	3.1	4’’’’		4.01	br d	3.0
5’’’’		3.91	m		5’’’’		3.99	m	
6’’’’		1.53	d	5.8	6’’’’		1.59	d	6.0
Rha					Rha				
1’’’’’		6.00	br s		1’’’’’		6.32	br s	
2’’’’’		5.11	br s		2’’’’’		4.97	br s	
3’’’’’		4.80	dd	9.0, 2.7	3’’’’’		4.64	br d	9.0
4’’’’’		4.52	m		4’’’’’		4.53	dd	9.0, 8.7
5’’’’’		4.50	m		5’’’’’		4.55	m	
6’’’’’		1.66	d	5.8	6’’’’’		1.66	d	4.8
Glc					Glc				
1’’’’’’		5.39	d	7.7	1’’’’’’		5.24	d	7.8
2’’’’’’		4.02	dd	8.8, 7.7	2’’’’’’		3.97	dd	9.0, 7.8
3’’’’’’		4.13	dd	8.8, 8.8	3’’’’’’		4.04	dd	9.0, 9.0
4’’’’’’		3.91	dd	8.8, 8.8	4’’’’’’		3.91	dd	9.0, 9.0
5’’’’’’		4.19	m		5’’’’’’		4.09	m	
6’’’’’’	a	4.50	m		6’’’’’’	a	4.91	br d	11.4
	b	4.28	dd	11.8, 5.3		b	4.65	m	
Xyl (II)					Xyl (II)				
1’’’’’’’		5.50	d	7.9	1’’’’’’’		5.48	d	7.8
2’’’’’’’		3.95	dd	9.1, 7.9	2’’’’’’’		3.91	dd	8.7, 7.8
3’’’’’’’		4.14	m		3’’’’’’’		4.09	dd	8.7, 7.8
4’’’’’’’		4.12	m		4’’’’’’’		4.12	m	
5’’’’’’’	a	4.17	m		5’’’’’’’	a	4.17	m	
	b	3.42	dd	9.1, 9.1		b	3.40	dd	10.2, 10.2
Qui					Qui				
1’’’’’’’’		4.98	d	7.9	1’’’’’’’’		4.97	d	6.6
2’’’’’’’’		3.98	m		2’’’’’’’’		4.01	dd	8.7, 6.6
3’’’’’’’’		3.98	m		3’’’’’’’’		4.04	dd	8.7, 8.7
4’’’’’’’’		4.08	dd	8.8, 8.8	4’’’’’’’’		3.62	m	
5’’’’’’’’		3.63	m		5’’’’’’’’		3.65	m	
6’’’’’’’’		1.54	d	6.5	6’’’’’’’’		1.54	d	6.0
					Ac		2.14	s	
**3**					**4**				
Positions		δ_H_		*J* (Hz)	Positions		δ_H_		*J* (Hz)
GlcUA					GlcUA				
1’		4.89	d	7.2	1’		4.89	d	7.2
2’		4.33	dd	9.0, 7.2	2’		4.33	dd	8.4, 7.2
3’		4.27	dd	9.0, 9.0	3’		4.25	dd	9.0, 8.4
4’		4.45	dd	9.6, 9.0	4’		4.45	dd	9.6, 9.0
5’		4.51	d	9.6	5’		4.50	d	9.6
Gal					Gal				
1’’		5.55	d	7.8	1’’		5.55	*	
2’’		4.47	dd	9.0, 7.8	2’’		4.46	dd	9.0, 7.8
3’’		4.15	dd	9.0, 3.6	3’’		4.14	dd	9.0, 3.6
4’’		4.57	br d	3.6	4’’		4.57	br d	3.6
5’’		4.00	m		5’’		3.99	m	
6’’	a	4.53	dd	10.8, 6.6	6’’	a	4.52	m	
	b	4.41	dd	10.8, 5.4		b	4.41	dd	10.2, 6.0
Xyl (I)					Xyl (I)				
1’’’		5.32	d	7.8	1’’’		5.32	d	7.8
2’’’		3.97	dd	9.0, 7.8	2’’’		3.96	dd	8.4, 7.8
3’’’		4.11	m		3’’’		4.11	dd	8.4, 7.8
4’’’		4.12	m		4’’’		4.12	m	
5’’’	a	4.25	dd	10.2, 5.4	5’’’	a	4.23	m	
	b	3.65	dd	10.2, 9.0		b	3.65	dd	9.0, 8.1
Fuc					Fuc				
1’’’’		5.94	d	8.4	1’’’’		5.92	d	8.4
2’’’’		4.57	dd	9.0, 8.4	2’’’’		4.48	dd	9.0, 8.4
3’’’’		4.16	dd	9.0, 3.6	3’’’’		4.18	dd	9.0, 3.0
4’’’’		3.97	br d	3.6	4’’’’		3.99	br d	3.0
5’’’’		3.93	dd	6.6, 3.6	5’’’’		3.93	m	
6’’’’		1.56	d	6.6	6’’’’		1.51	d	6.0
Rha					Rha				
1’’’’’		6.34	br s		1’’’’’		6.20	br s	
2’’’’’		4.74	br s		2’’’’’		4.70	br s	
3’’’’’		4.65	dd	9.0, 3.0	3’’’’’		4.61	br d	9.0
4’’’’’		4.37	dd	9.6, 9.0	4’’’’’		4.34	dd	9.0, 8.7
5’’’’’		4.44	dd	9.6, 6.0	5’’’’’		4.38	m	
6’’’’’		1.62	d	6.0	6’’’’’		1.54	d	4.8
Xyl (II)					Xyl (II)				
1’’’’’’		5.16	d	7.2	1’’’’’’		5.15	d	7.2
2’’’’’’		4.00	dd	9.0, 7.2	2’’’’’’		3.98	dd	9.0, 7.2
3’’’’’’		4.01	m		3’’’’’’		4.01	m	
4’’’’’’		4.02	m		4’’’’’’		4.01	m	
5’’’’’’	a	4.14	dd	12.0, 4.8	5’’’’’’	a	4.12	dd	12.0, 5.4
	b	3.40	dd	12.0, 10.2		b	3.39	dd	12.0, 9.6
Glc					Glc				
1’’’’’’’		5.18	d	7.8	1’’’’’’’		5.18	d	7.8
2’’’’’’’		4.08	dd	9.0, 7.8	2’’’’’’’		4.08	dd	9.0, 7.8
3’’’’’’’		4.22	dd	9.0, 9.0	3’’’’’’’		4.21	dd	9.0, 9.0
4’’’’’’’		4.16	dd	9.0, 9.0	4’’’’’’’		4.16	dd	9.0, 9.0
5’’’’’’’		3.96	m		5’’’’’’’		3.96	m	
6’’’’’’’	a	4.48	dd	10.8, 4.2	6’’’’’’’	a	4.48	dd	11.4, 4.2
	b	4.26	dd	10.8, 6.0		b	4.26	dd	11.4, 5.4
Qui					Qui				
1’’’’’’’’		4.97	d	7.8	1’’’’’’’’		5.05	d	7.2
2’’’’’’’’		4.00	dd	9.0, 7.8	2’’’’’’’’		4.00	dd	9.6, 7.2
3’’’’’’’’		4.05	dd	9.0, 9.0	3’’’’’’’’		5.60	dd	9.6, 9.6
4’’’’’’’’		3.62	dd	9.0, 9.0	4’’’’’’’’		5.05	dd	9.6, 9.6
5’’’’’’’’		3.66	dd	9.0, 6.0	5’’’’’’’’		3.69	dd	9.6, 6.0
6’’’’’’’’		1.55	d	6.0	6’’’’’’’’		1.24	d	6.0
					3’’’’’’’’-OAc		1.96	s	
					4’’’’’’’’-OAc		2.04	s	
**5**					**6**				
Positions		δ_H_		*J* (Hz)	Positions		δ_H_		*J* (Hz)
Xyl					Xyl				
1’		4.99	d	7.4	1’		4.97	d	7.4
2’		3.96	dd	8.6, 7.4	2’		3.94	dd	8.6, 7.4
3’		4.06	dd	8.6, 8.6	3’		4.05	dd	8.6, 8.6
4’		4.20	m		4’		4.18	m	
5’	a	4.35	dd	11.5, 5.2	5’	a	4.34	dd	11.6, 4.9
	b	3.68	dd	11.5, 10.0		b	3.67	dd	11.6, 11.0
Glc (I)					Glc (I)				
1’’		6.22	d	7.7	1’’		6.17	d	7.1
2’’		4.18	dd	9.2, 7.7	2’’		4.24	m	
3’’		4.21	dd	9.2, 9.2	3’’		4.25	m	
4’’		4.55	dd	9.2, 9.2	4’’		4.33	m	
5’’		4.05	m		5’’		4.11	m	
6’’	a	4.56	br d	10.8	6’’	a	4.53	br d	11.5
	b	4.29	dd	10.8, 3.9		b	4.30	dd	11.5, 3.6
Glc (II)					Glc (II)				
1’’’		4.91	d	7.8	1’’’		5.28	d	7.8
2’’’		3.99	dd	9.1, 7.8	2’’’		4.05	m	
3’’’		4.25	dd	9.1, 9.1	3’’’		4.14	dd	9.2, 9.2
4’’’		4.20	dd	9.1, 9.1	4’’’		4.13	dd	9.2, 9.2
5’’’		3.76	m		5’’’		3.91	m	
6’’’	a	4.38	dd	12.3, 2.2	6’’’	a	4.44	br d	11.1
	b	4.31	br d	12.3		b	4.25	dd	11.1, 5.6
Glc (III)					Glc (III)				
1’’’’		5.26	d	7.8	1’’’’		4.99	d	7.7
2’’’’		4.03	dd	8.5, 7.8	2’’’’		4.06	dd	8.8, 7.7
3’’’’		4.17	m		3’’’’		4.22	dd	8.8, 8.8
4’’’’		4.18	m		4’’’’		3.99	dd	8.8, 8.8
5’’’’		3.90	m		5’’’’		3.89	m	
6’’’’	a	4.51	dd	12.1, 2.2	6’’’’	a	4.92	br d	11.2
	b	4.34	br d	12.1		b	4.68	dd	11.2, 5.8
					Glc (IV)				
					1’’’’’		5.28	d	7.8
					2’’’’’		4.05	m	
					3’’’’’		4.18	dd	8.5, 8.5
					4’’’’’		4.15	dd	8.5, 8.5
					5’’’’’		3.90	m	
					6’’’’’	a	4.54	br d	11.8
						b	4.34	dd	11.8, 5.2
					HMG				
					1’’’’’’		–		
					2’’’’’’	a	3.16	d	14.3
						b	3.11	d	14.3
					3’’’’’’		–		
					4’’’’’’	a	3.18	d	15.0
						b	3.15	d	15.0
					5’’’’’’		–		
					6’’’’’’		1.75	s	
**7**									
Positions		δ_H_		*J* (Hz)					
Xyl									
1’		4.97	d	7.7					
2’		3.95	dd	8.2, 7.7					
3’		4.05	dd	8.9, 8.2					
4’		4.19	m						
5’	a	4.35	dd	11.4, 5.0					
	b	3.68	dd	11.4, 10.6					
Glc (I)									
1’’		6.18	d	7.9					
2’’		4.20	m						
3’’		4.24	dd	9.1, 9.1					
4’’		4.27	dd	9.1, 9.1					
5’’		4.11	m						
6’’	a	4.52	br d	11.6					
	b	4.29	m						
Glc (II)									
1’’’		5.28	d	7.7					
2’’’		4.05	m						
3’’’		4.14	dd	9.0, 9.0					
4’’’		4.13	dd	9.0, 9.0					
5’’’		3.90	m						
6’’’	a	4.45	dd	11.8, 2.0					
	b	4.25	dd	11.8, 5.1					
Glc (III)									
1’’’’		4.98	d	7.3					
2’’’’		4.05	dd	9.2, 7.3					
3’’’’		4.23	dd	9.2, 9.2					
4’’’’		3.99	dd	9.2, 9.2					
5’’’’		3.90	m						
6’’’’	a	4.93	br d	10.5					
	b	4.69	dd	10.5, 5.8					
Glc (IV)									
1’’’’’		5.29	d	7.6					
2’’’’’		4.05	m						
3’’’’’		4.17	dd	8.6, 8.6					
4’’’’’		4.18	dd	8.6, 8.6					
5’’’’’		3.90	m						
6’’’’’	a	4.52	br d	11.7					
	b	4.33	dd	11.7, 4.8					
HMG									
1’’’’’’		–							
2’’’’’’	a	3.18	d	14.3					
	b	3.12	d	14.3					
3’’’’’’		–							
4’’’’’’	a	3.19	d	15.1					
	b	3.15	d	15.1					
5’’’’’’		–							
6’’’’’’		1.75	s						

The ^1^H-NMR spectra of **1**–**7** were recorded at 600 MHz in C_5_D_5_N. * Signals are overlapped with water signal.

**Table 3:** ^13^C-NMR spectral data for **1**–**7**.

Positions	1	2	3	4	5	6	7
1	38.1	38.2	38.1	38.2	38.9	38.8	38.9
2	25.3	25.3	25.3	25.3	26.3	26.3	26.3
3	84.4	84.4	84.5	84.4	85.0	85.0	85.0
4	55.0	55.1	55.1	55.1	53.3	53.3	53.3
5	48.9	49.0	48.8	49.0	52.1	52.1	52.2
6	20.5	20.7	20.6	20.7	21.3	21.3	21.3
7	33.0	33.1	32.8	33.1	33.1	32.8	33.1
8	40.3	40.3	40.3	40.3	40.4	40.2	40.3
9	46.9	47.0	46.9	47.0	47.4	48.3	47.4
10	36.2	36.3	36.3	36.3	36.7	36.7	36.7
11	23.7	23.7	23.7	23.7	23.8	23.8	23.8
12	121.9	121.8	122.1	121.8	122.3	122.7	122.5
13	144.5	144.5	144.5	144.5	144.5	144.0	144.4
14	42.1	42.2	42.1	42.2	42.0	42.1	42.0
15	36.2	36.4	36.2	36.4	36.0	28.2	36.0
16	74.2	74.0	74.0	74.0	74.1	23.1	74.0
17	49.2	49.3	49.2	49.3	49.0	47.0	49.0
18	41.7	41.9	41.6	41.9	41.2	41.6	41.2
19	47.5	47.7	47.3	47.7	47.1	46.2	47.1
20	30.6	30.6	30.7	30.6	30.7	30.7	30.7
21	36.0	36.1	36.0	36.1	35.8	33.9	35.8
22	31.6	31.4	31.9	31.4	32.0	32.4	32.0
23	210.1	210.2	210.2	210.2	180.3	180.4	180.4
24	11.1	11.2	11.1	11.2	12.6	12.5	12.6
25	15.9	15.9	15.8	15.9	16.1	16.0	16.1
26	17.4	17.4	17.4	17.4	17.4	17.3	17.4
27	27.1	27.2	27.0	27.2	27.1	26.0	27.1
28	175.9	176.0	176.0	176.0	175.9	176.3	175.8
29	33.1	33.0	33.1	33.0	33.1	33.0	33.1
30	24.4	24.4	24.5	24.4	24.5	23.7	24.6
	GlcUA	GlcUA	GlcUA	GlcUA	Xyl	Xyl	Xyl
1’	103.9	103.9	103.9	103.9	106.2	106.2	106.2
2’	78.6	78.7	78.4	78.4	75.2	75.2	75.2
3’	86.0	86.0	86.1	86.1	78.0	78.0	78.0
4’	71.3	71.3	71.3	71.3	71.0	71.1	71.0
5’	77.2	77.2	77.3	77.3	67.0	67.0	67.0
6’	171.7	171.7	171.7	171.7			
	Gal	Gal	Gal	Gal	Glc (I)	Glc (I)	Glc (I)
1’’	104.2	104.2	104.1	104.1	95.7	94.8	94.9
2’’	73.7	73.7	73.6	73.6	74.0	73.0	73.0
3’’	75.4	75.2	75.5	75.5	78.5	88.2	88.0
4’’	70.1	70.1	70.1	70.1	70.2	69.1	69.1
5’’	76.6	76.6	76.5	76.6	77.1	76.9	76.9
6’’	61.6	61.6	61.6	61.6	69.1	68.9	69.0
	Xyl (I)	Xyl (I)	Xyl (I)	Xyl (I)	Glc (II)	Glc (II)	Glc (II)
1’’’	105.0	105.0	105.0	105.0	102.7	105.7	105.7
2’’’	75.3	75.3	75.3	75.2	84.4	75.6	75.6
3’’’	78.5	78.6	78.6	78.6	78.1	77.9	78.1
4’’’	70.8	70.8	70.8	70.8	70.5	71.3	71.3
5’’’	67.3	67.3	67.3	67.3	78.3	78.5	78.5
6’’’					62.0	62.3	62.3
	Fuc	Fuc	Fuc	Fuc	Glc (III)	Glc (III)	Glc (III)
1’’’’	94.8	94.7	94.6	94.5	106.1	102.5	102.5
2’’’’	75.2	73.8	74.4	74.3	76.3	83.3	83.2
3’’’’	75.9	76.8	76.8	76.2	78.1	77.7	77.7
4’’’’	83.4	84.0	84.0	83.5	70.9	71.0	70.9
5’’’’	71.5	71.8	71.6	71.4	78.6	75.0	75.0
6’’’’	17.1	17.1	17.1	17.0	62.0	64.4	64.3
	Rha	Rha	Rha	Rha		Glc (IV)	Glc (IV)
1’’’’’	101.7	100.9	101.2	101.2		105.6	105.7
2’’’’’	70.8	71.2	71.8	71.7		76.2	76.2
3’’’’’	82.3	83.1	72.4	72.3		78.1	78.0
4’’’’’	78.2	78.2	83.5	83.4		71.2	71.1
5’’’’’	68.9	68.3	68.3	68.3		78.4	78.4
6’’’’’	18.9	18.8	18.6	18.5		62.5	62.4
	Glc	Glc	Xyl (II)	Xyl (II)		HMG	HMG
1’’’’’’	105.2	105.3	106.2	106.1		171.7	171.7
2’’’’’’	75.4	75.1	74.8	74.8		46.6	46.5
3’’’’’’	78.3	78.1	88.2	88.3		70.0	70.0
4’’’’’’	71.7	71.7	69.3	69.3		46.3	46.3
5’’’’’’	78.4	75.2	66.7	66.6		174.6	174.7
6’’’’’’	62.6	64.4				28.2	28.2
	Xyl (II)	Xyl (II)	Glc	Glc			
1’’’’’’’	104.9	104.8	105.5	105.5			
2’’’’’’’	75.6	75.7	75.4	75.3			
3’’’’’’’	79.1	79.1	78.3	78.3			
4’’’’’’’	71.2	71.2	71.5	71.5			
5’’’’’’’	67.1	67.1	78.6	78.6			
6’’’’’’’			62.5	62.4			
	Qui	Qui	Qui	Qui			
1’’’’’’’’	106.3	106.7	106.7	105.7			
2’’’’’’’’	75.7	75.8	75.8	73.1			
3’’’’’’’’	78.1	78.2	78.4	76.3			
4’’’’’’’’	76.7	76.7	76.6	74.4			
5’’’’’’’’	73.1	73.2	73.2	70.2			
6’’’’’’’’	18.5	18.5	18.5	17.7			
		6’’’’’’-OAc		3’’’’’’’’-OAc			
		21.0		20.6			
		170.9		170.4			
				4’’’’’’’’-OAc			
				20.7			
				170.1			

The ^13^C-NMR of **1**–**7** were recorded at 150 MHz in C_5_D_5_N.

## Supplementary Materials

The following are available online: https://www.mdpi.com/article/10.3390/ijms23042047/s1.
