# Supplementary material for: Novel Oleanane-Type Triterpene Glycosides from the Saponaria officinalis L. Seeds and Apoptosis-Inducing Activity via Mitochondria
Source: Int J Mol Sci. 2022 Feb 12;23(4):2047. doi: 10.3390/ijms23042047 (PMC8877789; doi:10.3390/ijms23042047)
Supplement: Supplementary file 1 [file ijms-23-02047-s001.zip › ijms-1591028-supplementary.pdf]

-Supplementary materials-

Novel Oleanane-Type Triterpene Glycosides from the *Saponaria officinalis* L. Seeds and Apoptosis-Inducing Activity via Mitochondria

Naoki Takahashi, Tomoki Iguchi *, Minpei Kuroda, Masaki Mishima, and Yoshihiro Mimaki

School of Pharmacy, Tokyo University of Pharmacy and Life Sciences, 1432-1, Horinouchi, Hachioji, Tokyo 192-0392, Japan; y20612@toyaku.ac.jp (N.T.), kurodam@toyaku.ac.jp (M.K.), mmisima@toyaku.ac.jp (M.M.), mimakiy@toyaku.ac.jp (Y.M.)

*Correspondence: iguchit@toyaku.ac.jp; Tel.: +81-42-676-4575

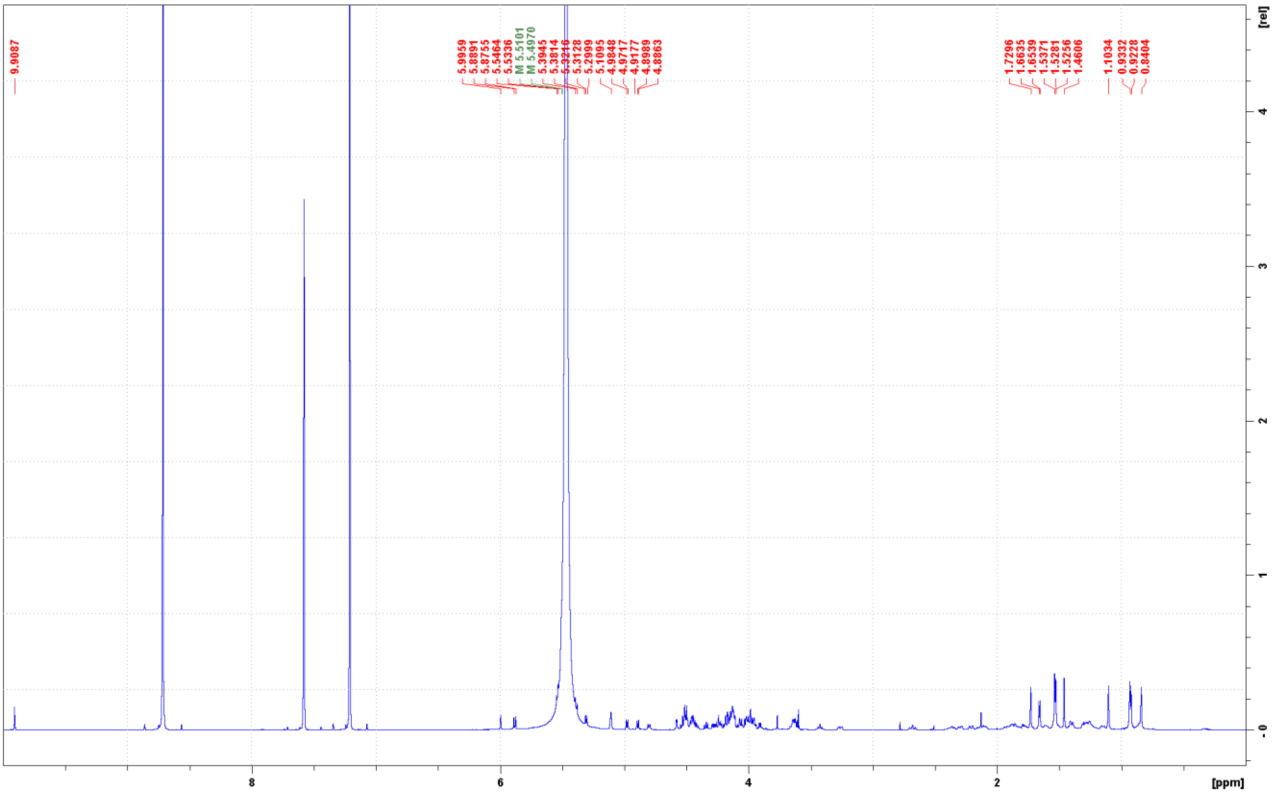

Figure S1. ¹H-NMR spectrum of **1**.

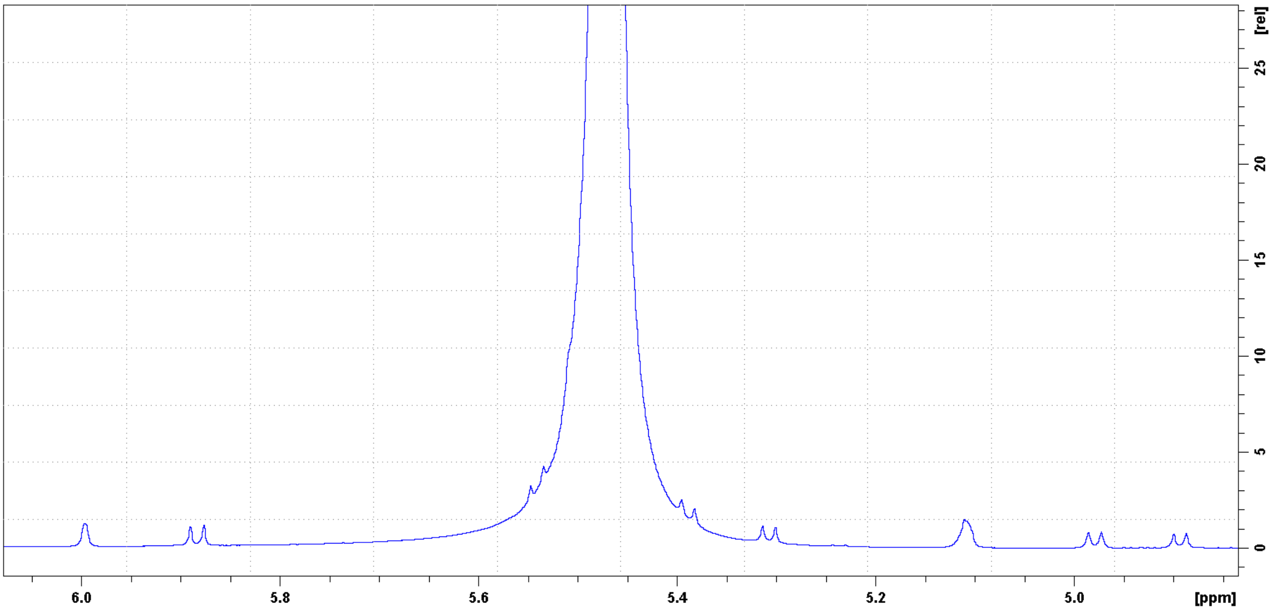

Figure S2. ¹H-NMR spectrum of **1** (expanded for anomeric region).

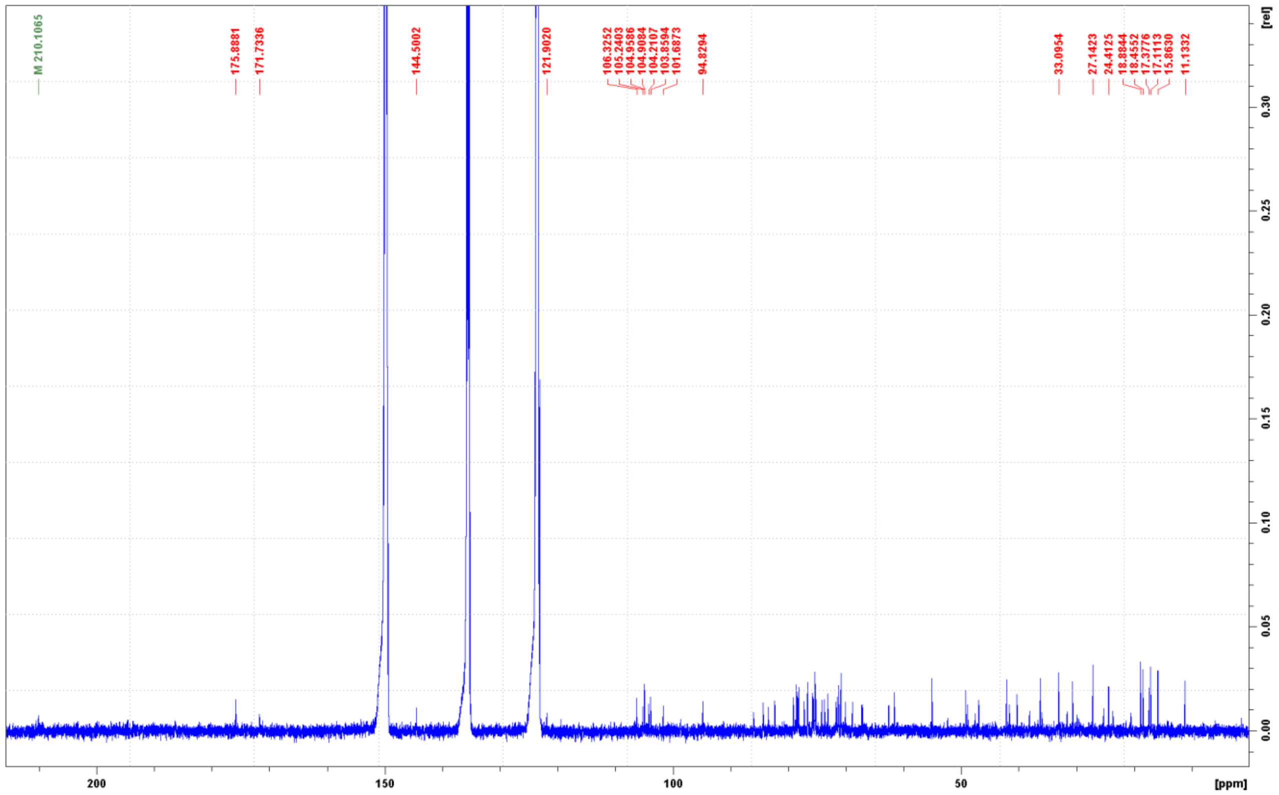

Figure S3. ^{13}C-NMR spectrum of **1**.

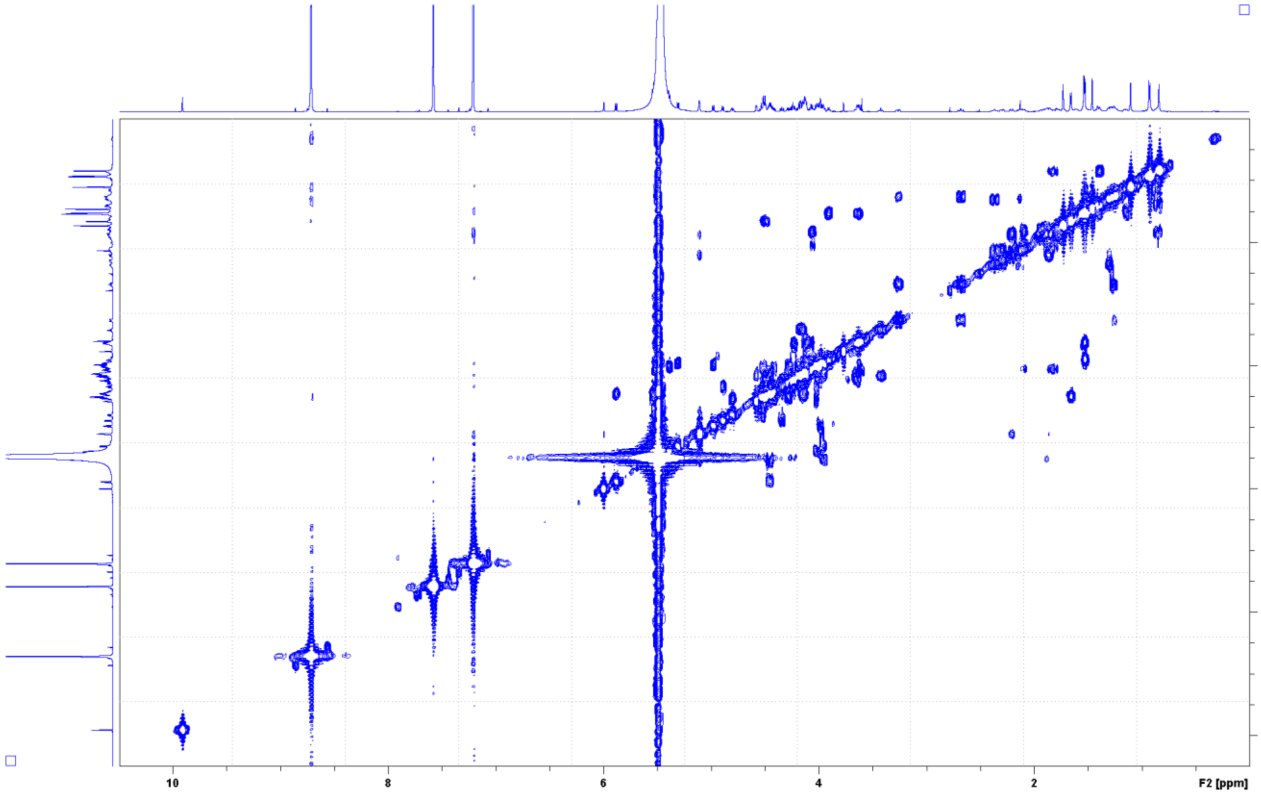

Figure S4. ^{1}H-^{1}H COSY spectrum of **1**.

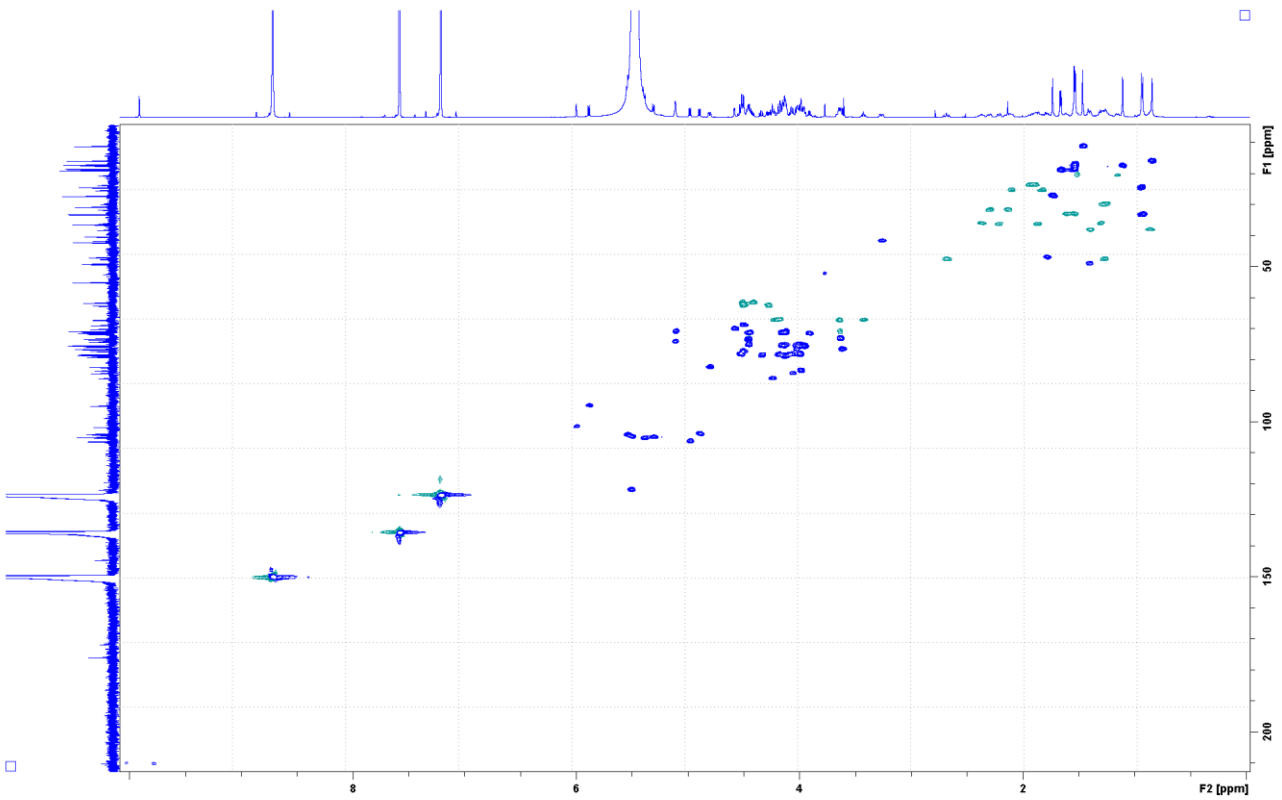

Figure S5. HSQC spectrum of **1**.

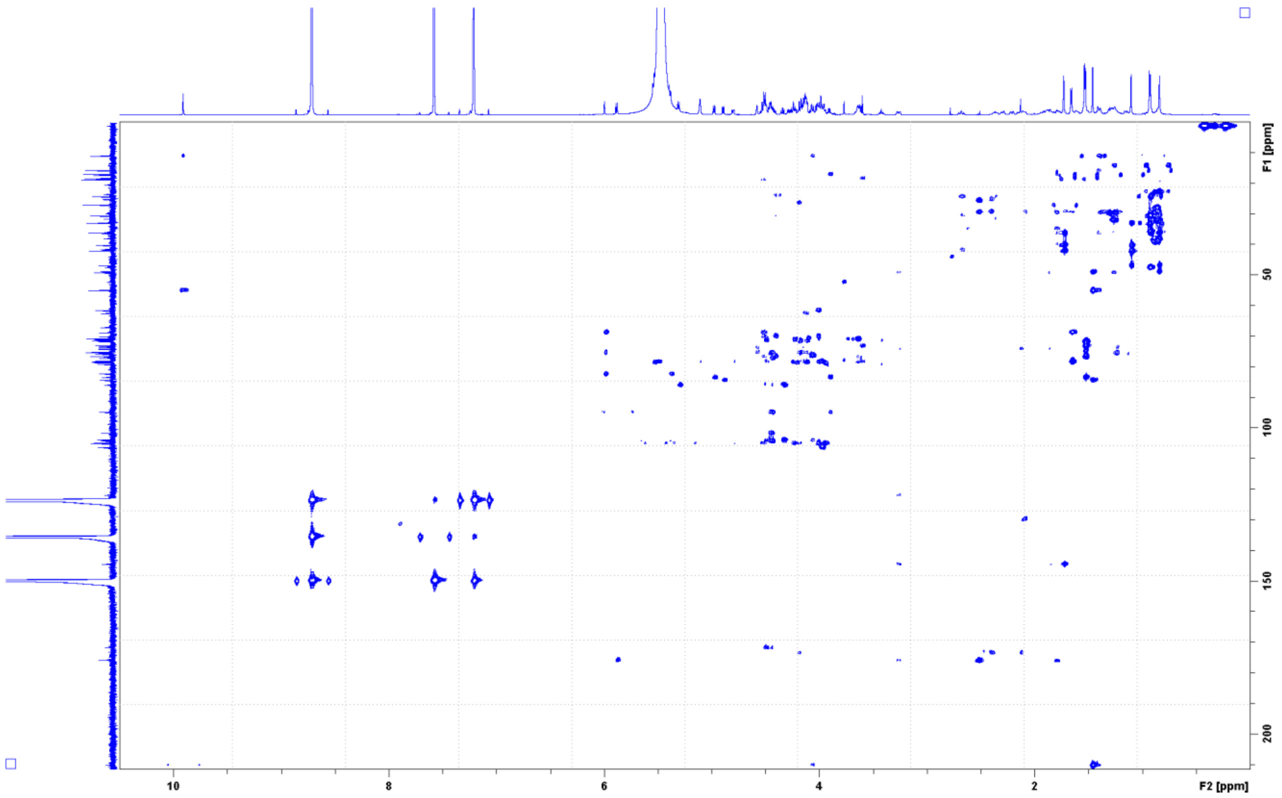

Figure S6. HMBC spectrum of **1**.

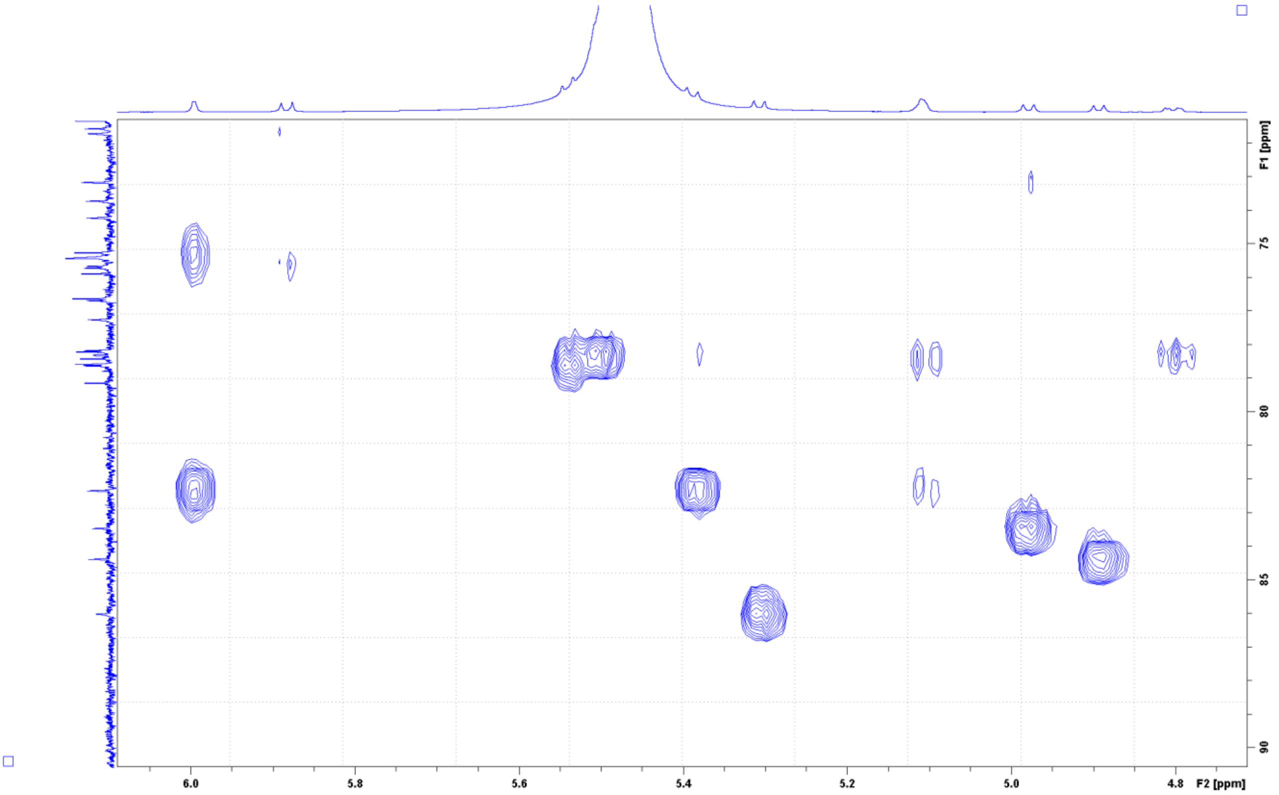

Figure S7. HMBC spectrum of **1** (expanded for anomeric region).

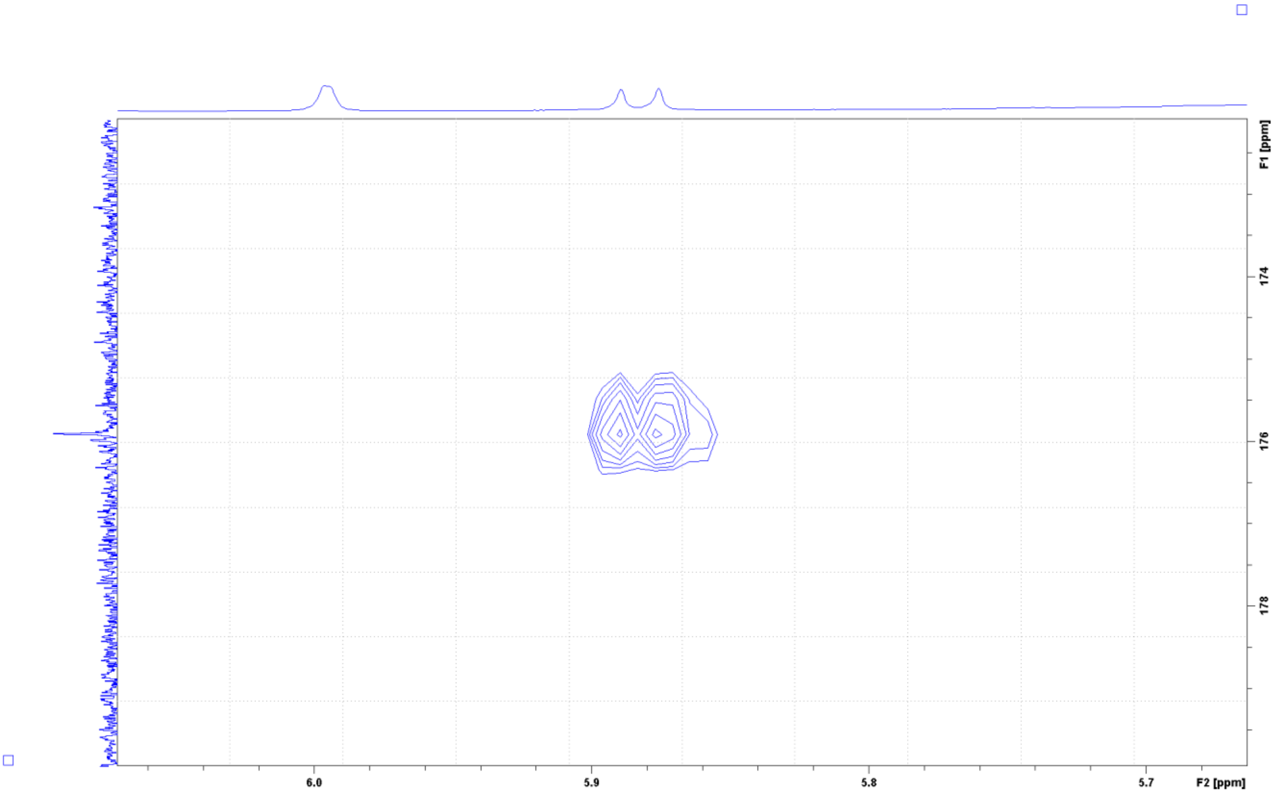

Figure S8. HMBC spectrum of **1** (expanded for between H-1′′′′ of Fuc and C-28 of the aglycone).

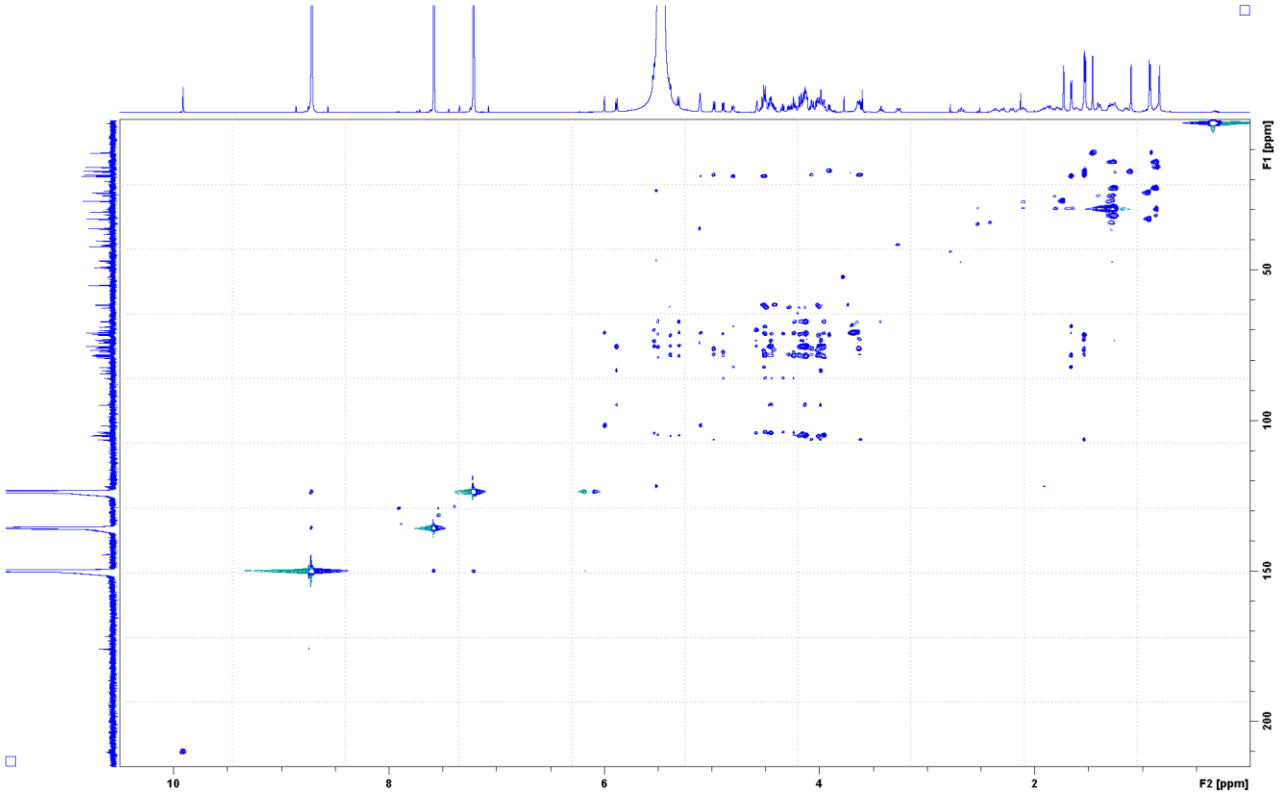

Figure S9. HSQC-TOCSY spectrum of **1**.

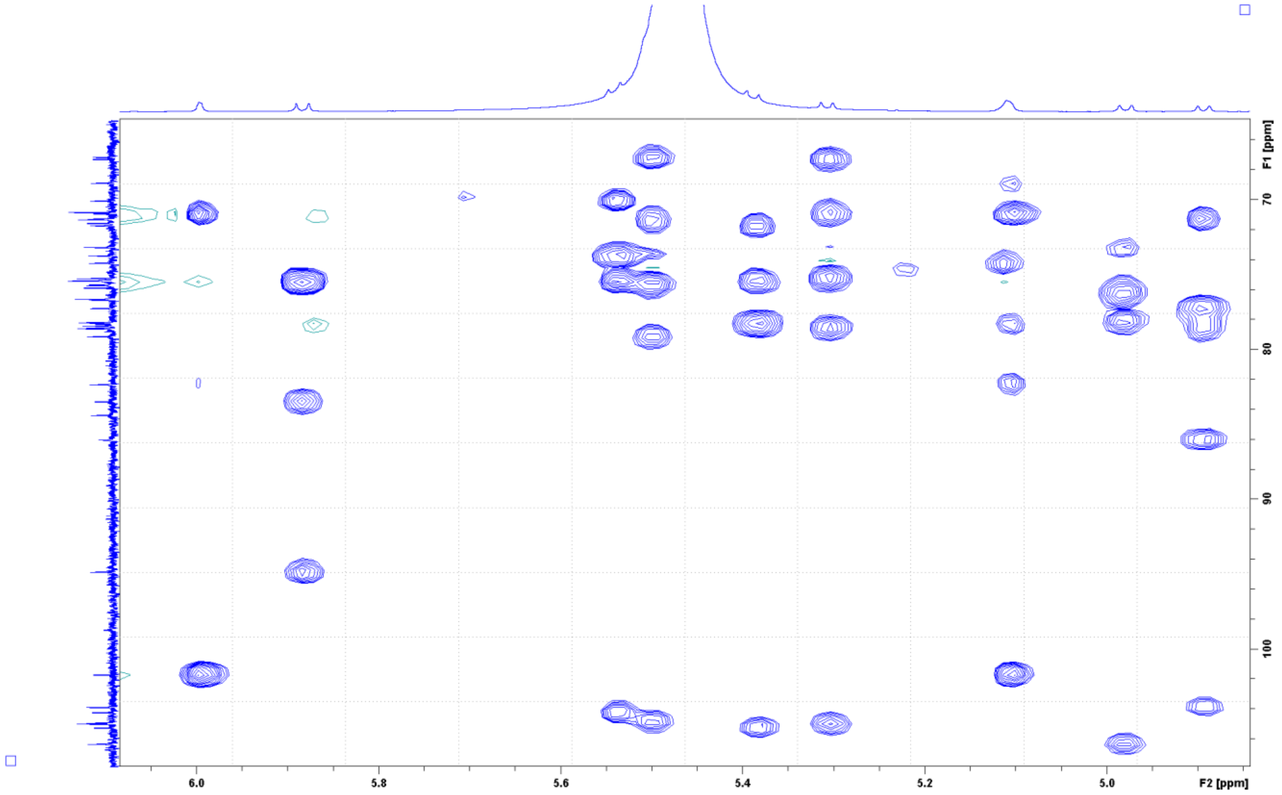

Figure S10. HSQC-TOCSY spectrum of **1** (expanded for anomeric region).

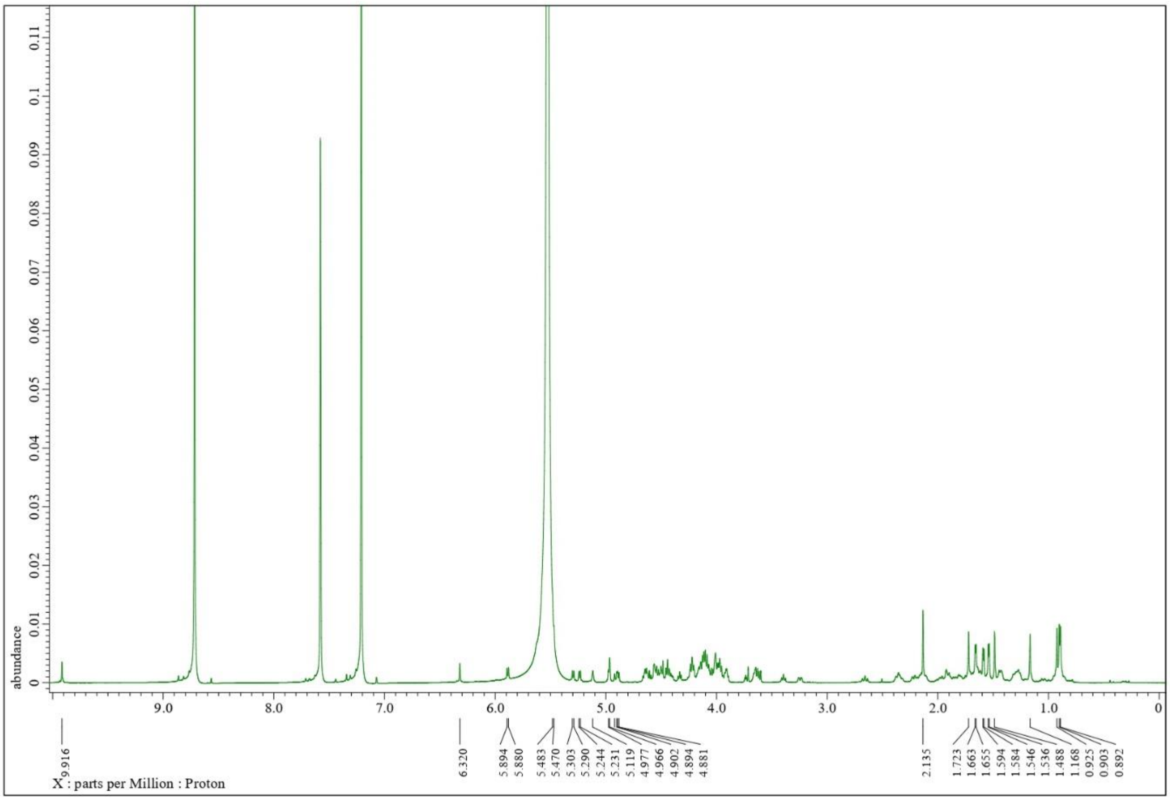

Figure S11. ^{1}H-NMR spectrum of **2**.

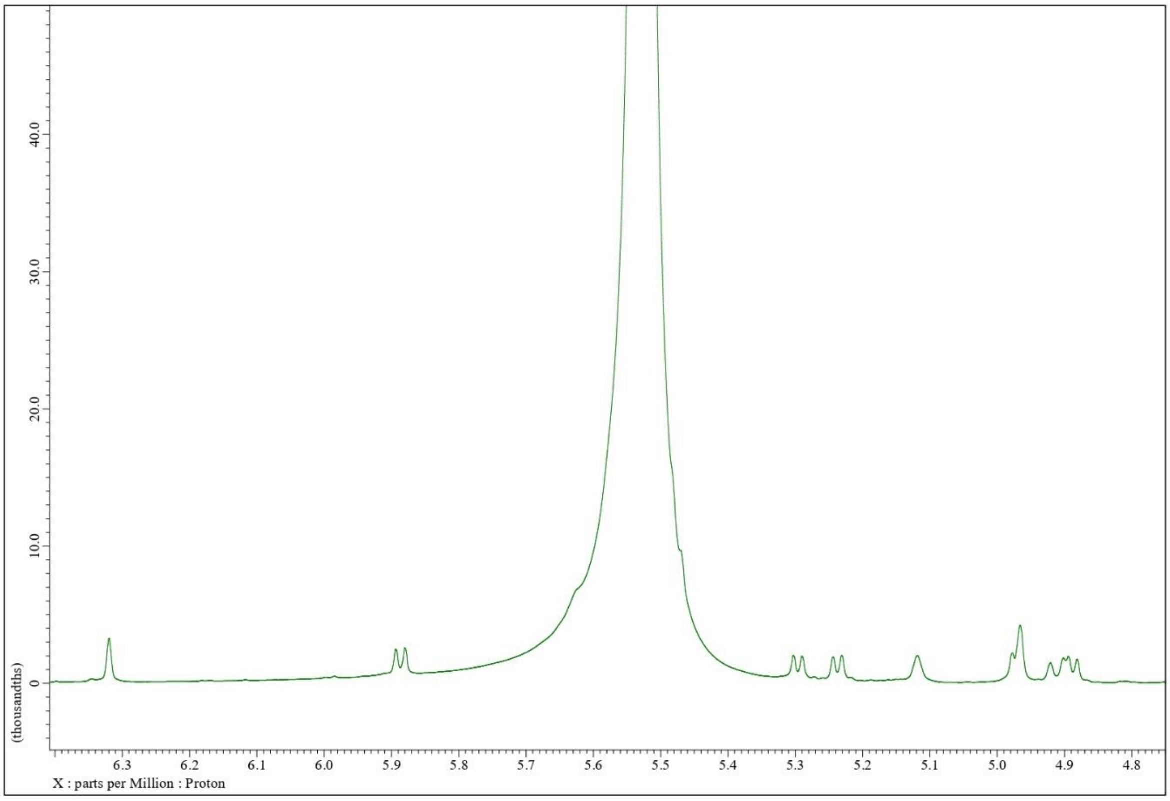

Figure S12. ^{1}H-NMR spectrum of **2** (expanded for anomeric region).

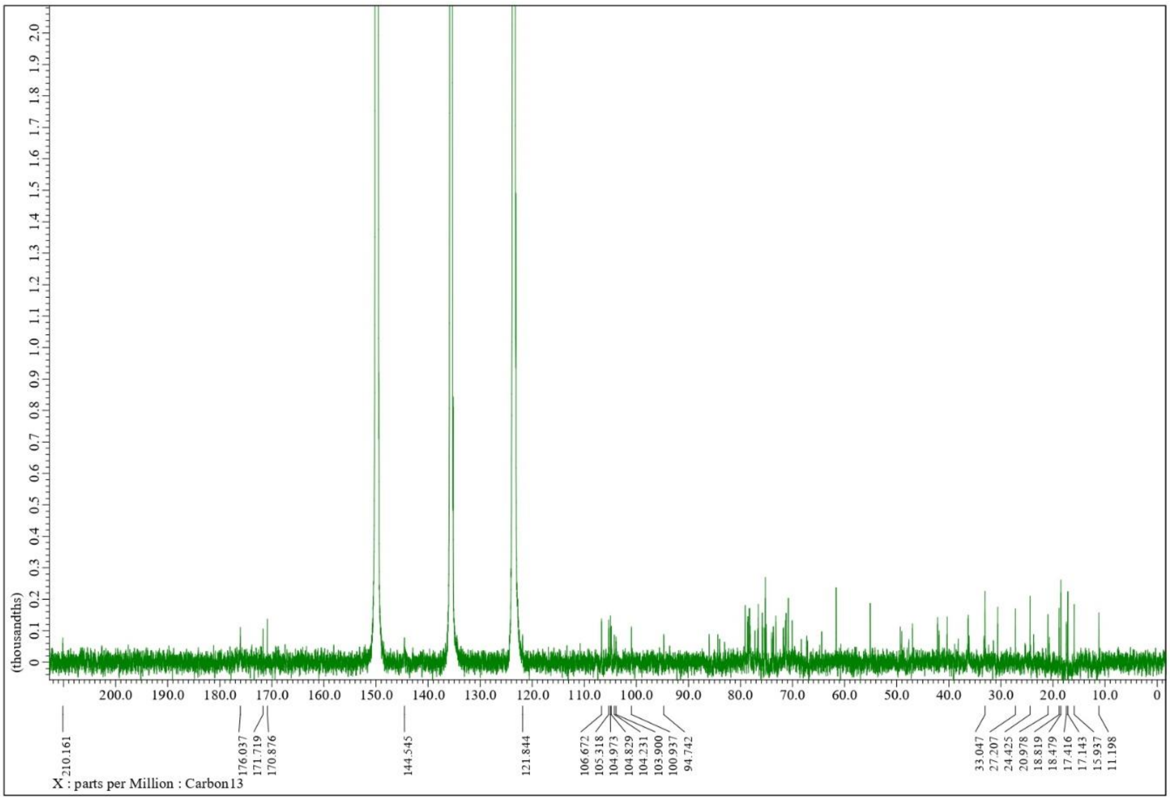

Figure S13. ^{13}C-NMR spectrum of **2**.

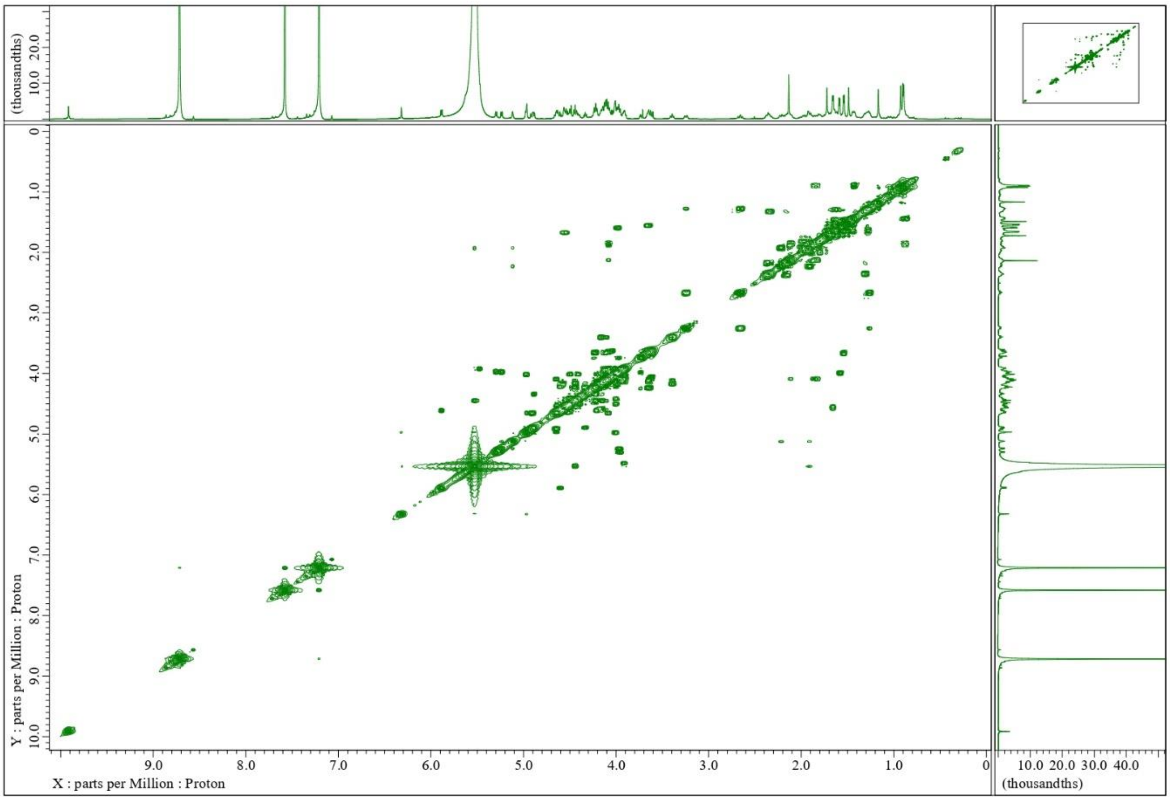

Figure S14. ^{1}H-^{1}H COSY spectrum of **2**.

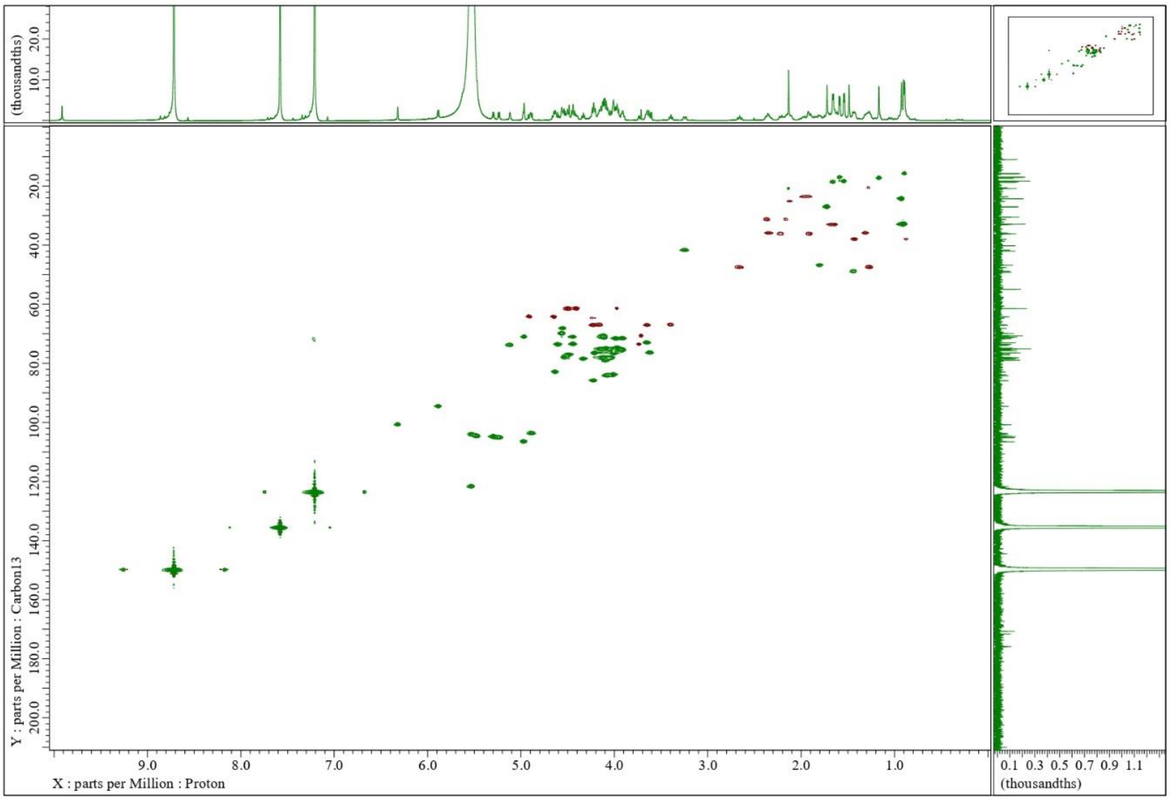

Figure S15. HSQC spectrum of **2**.

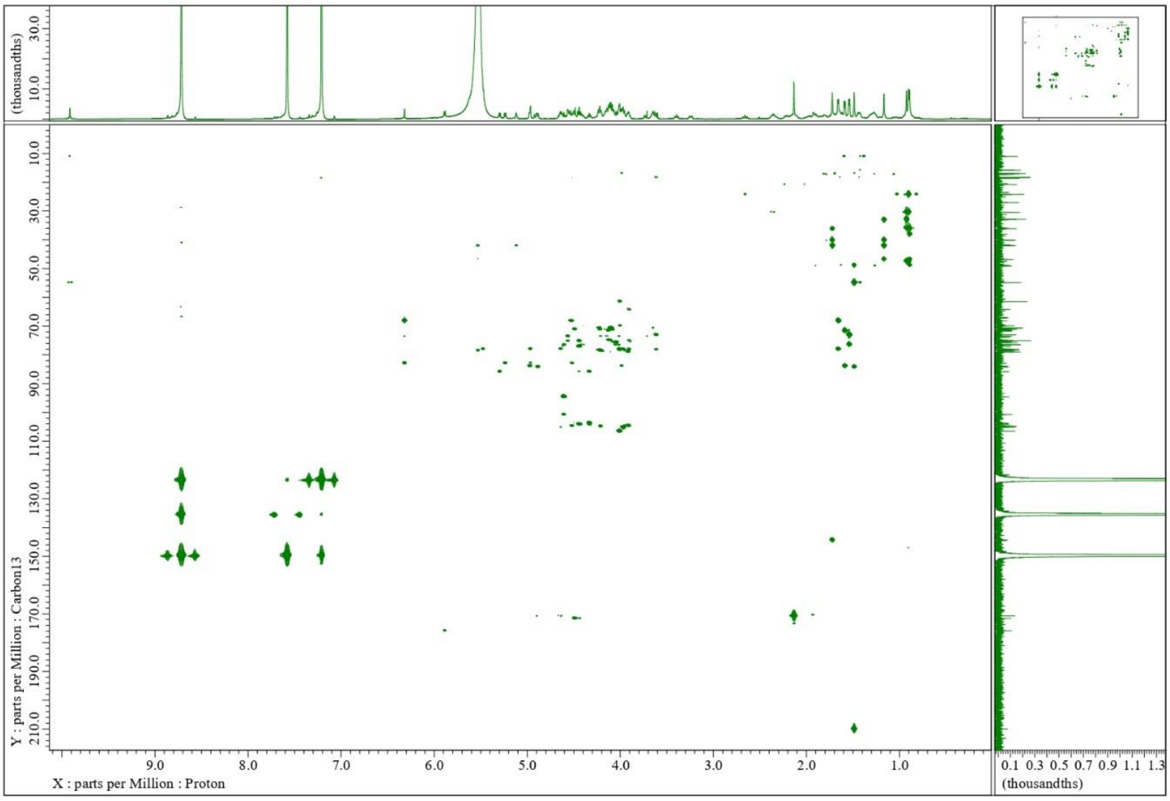

Figure S16. HMBC spectrum of **2**.

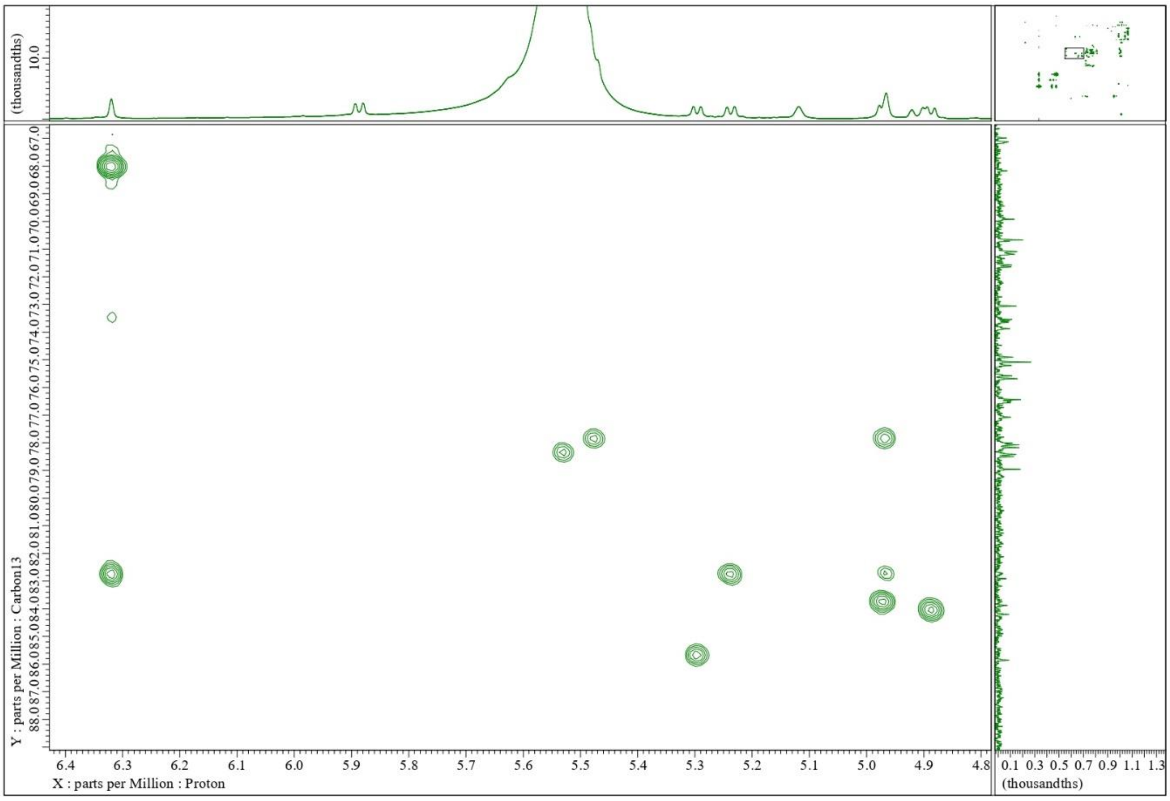

Figure S17. HMBC spectrum of **2** (expanded for anomeric region).

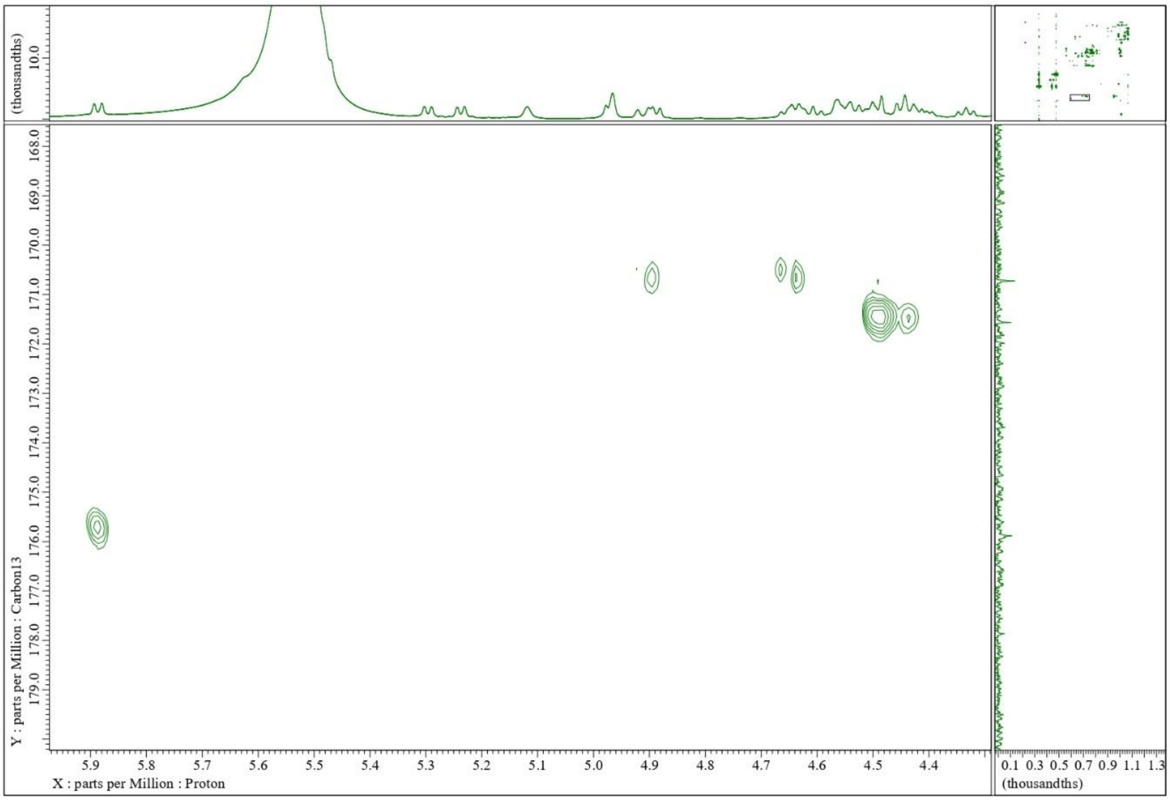

Figure S18. HMBC spectrum of **2** (expanded for between H-1′′′′ of Fuc and C-28 of the aglycone, and between H-6′′′′′′ of Glc and carbonyl carbon of acetyl moiety).

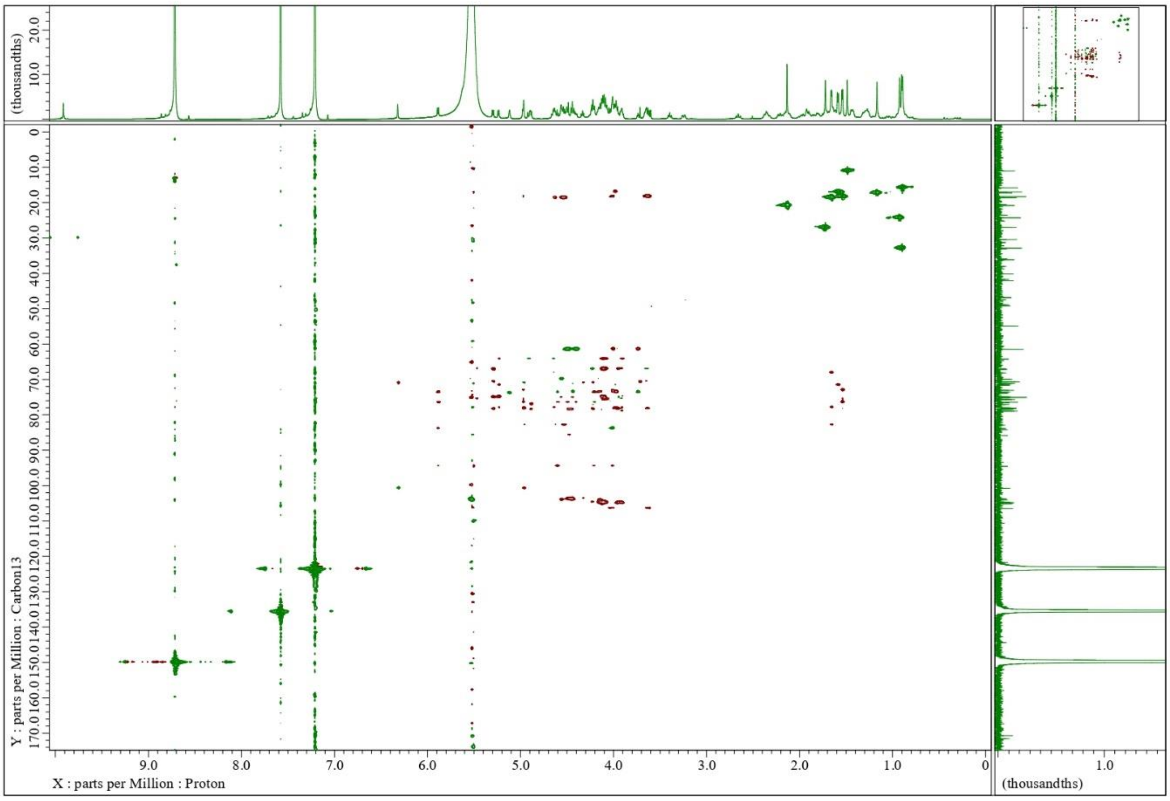

Figure S19. HSQC-TOCSY spectrum of **2**.

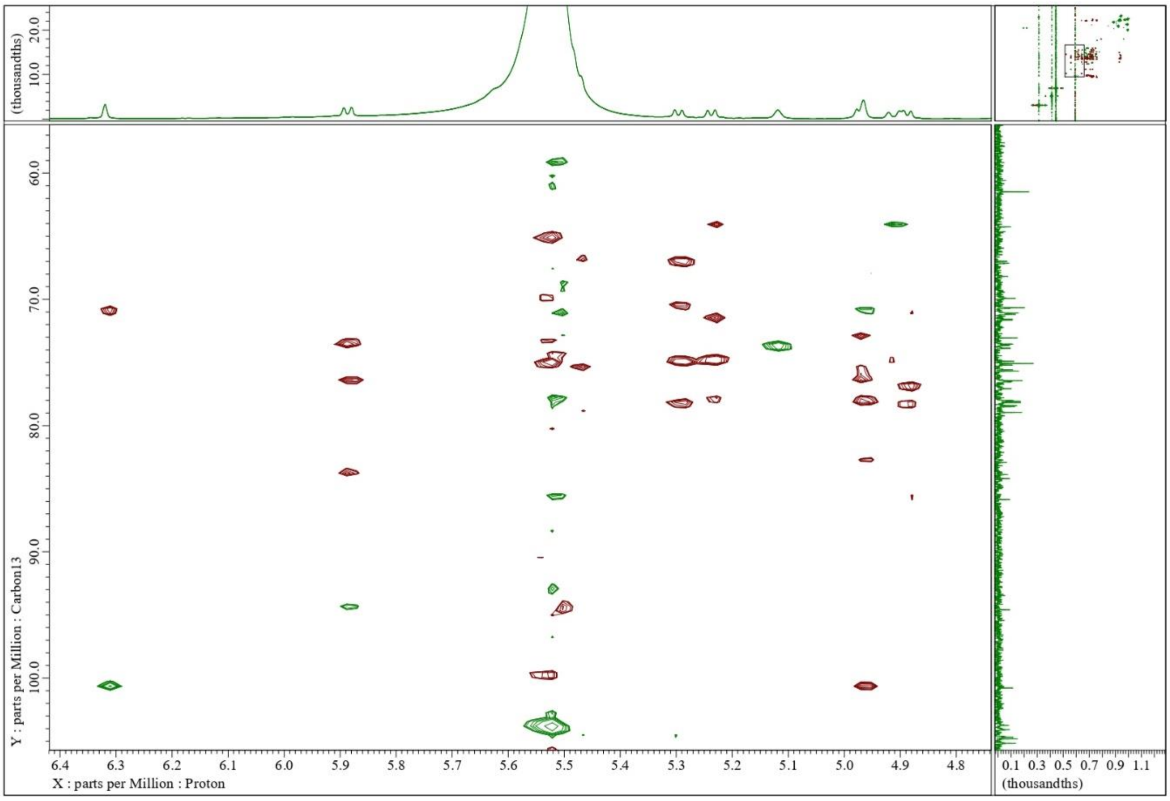

Figure S20. HSQC-TOCSY spectrum of **2** (expanded for anomeric region).

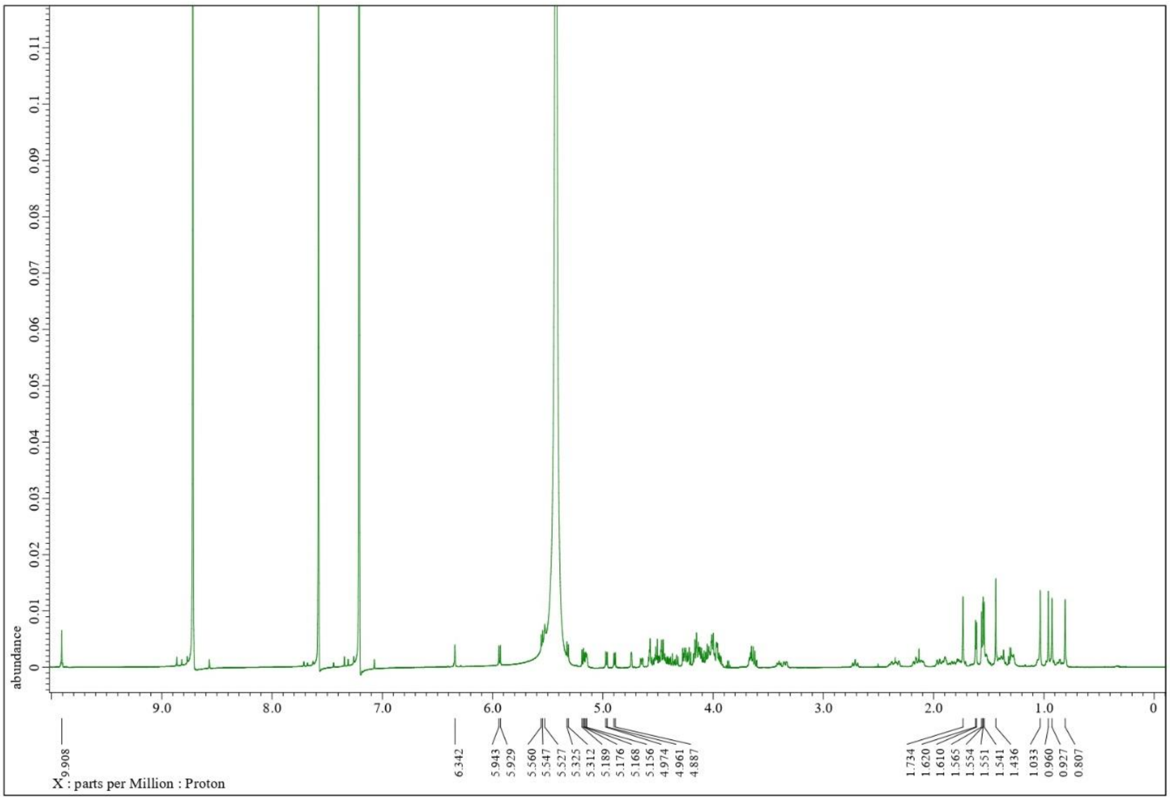

Figure S21. ^{1}H-NMR spectrum of **3**.

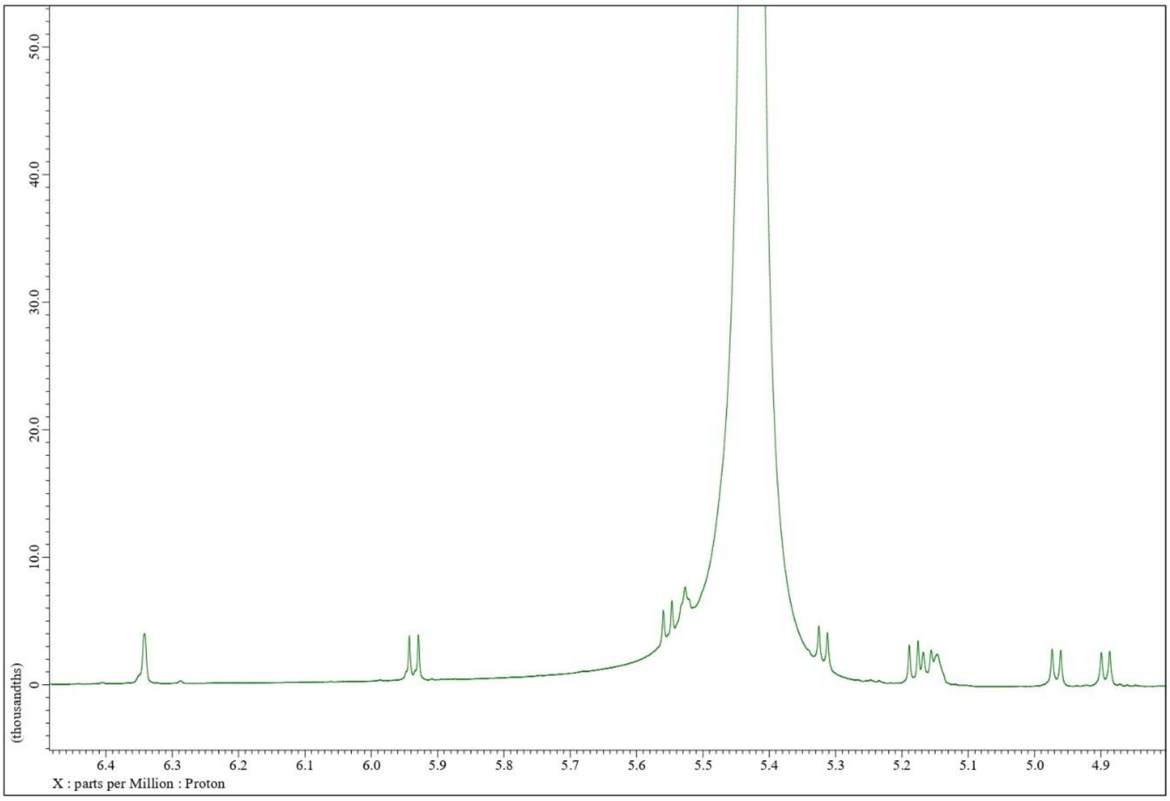

Figure S22. ^{1}H-NMR spectrum of **3** (expanded for anomeric region).

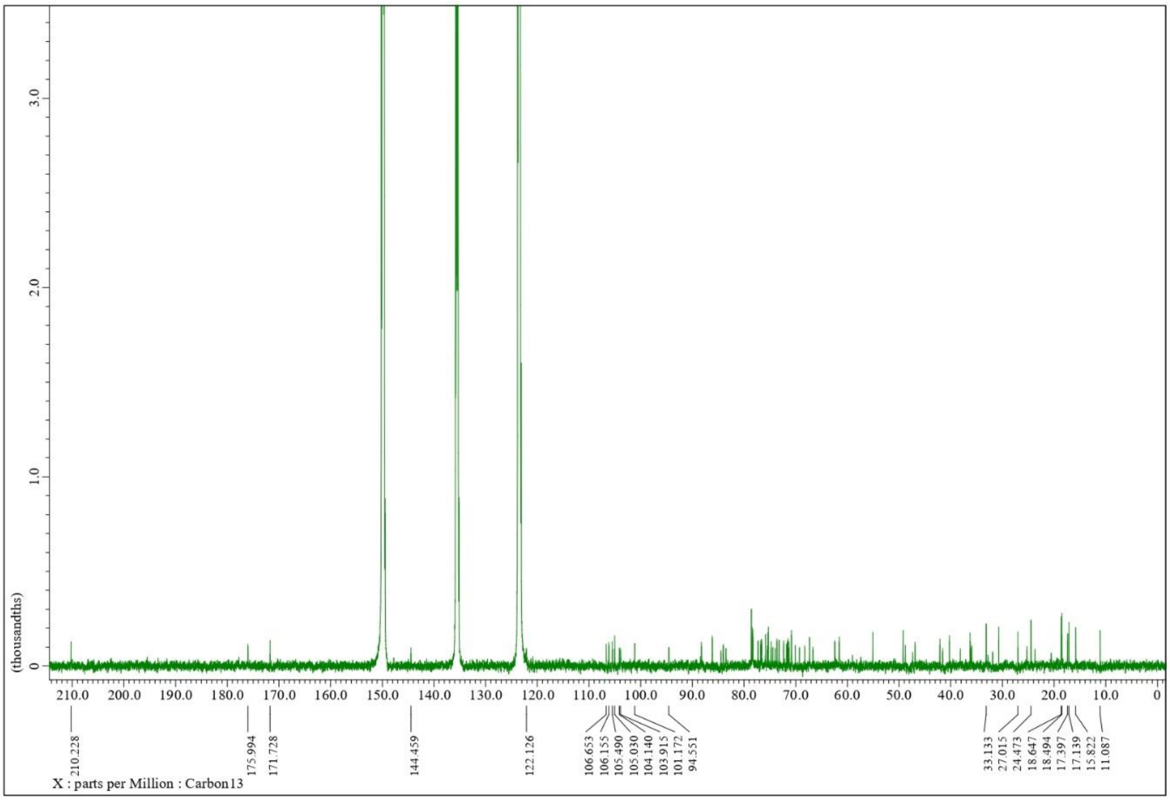

Figure S23. ^{13}C-NMR spectrum of **3**.

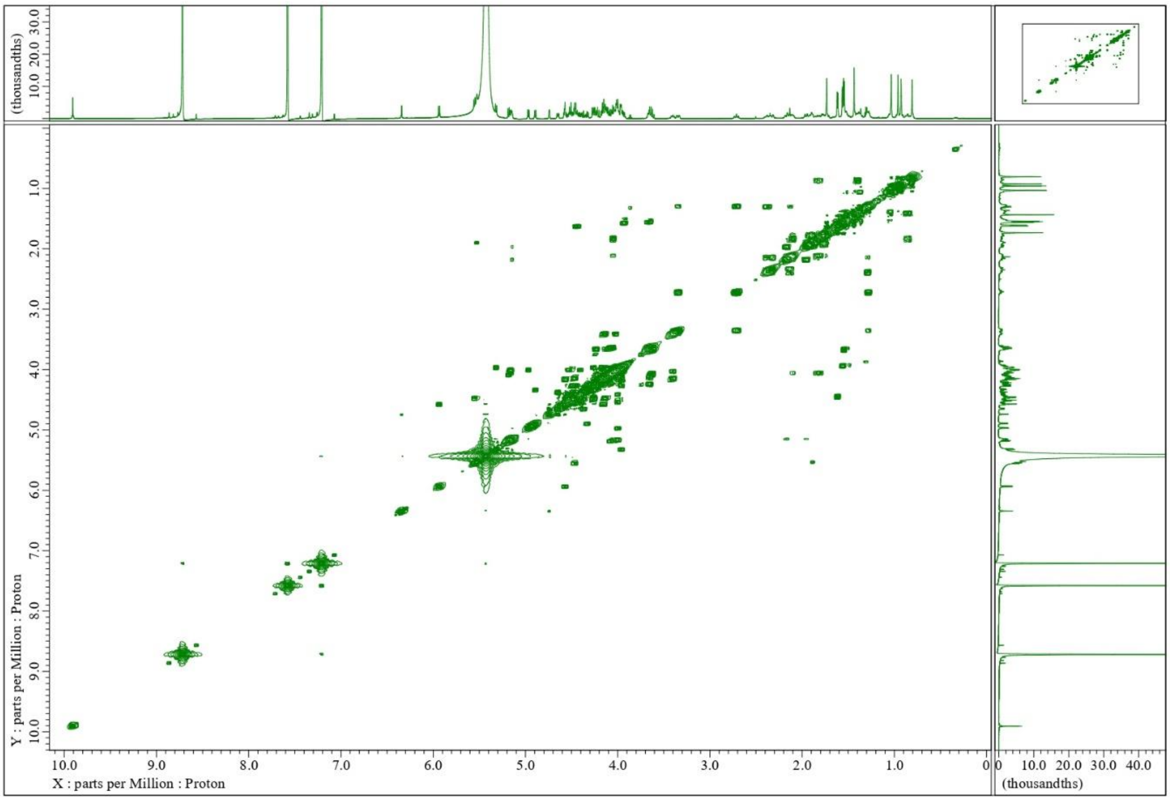

Figure S24. ^{1}H-^{1}H COSY spectrum of **3**.

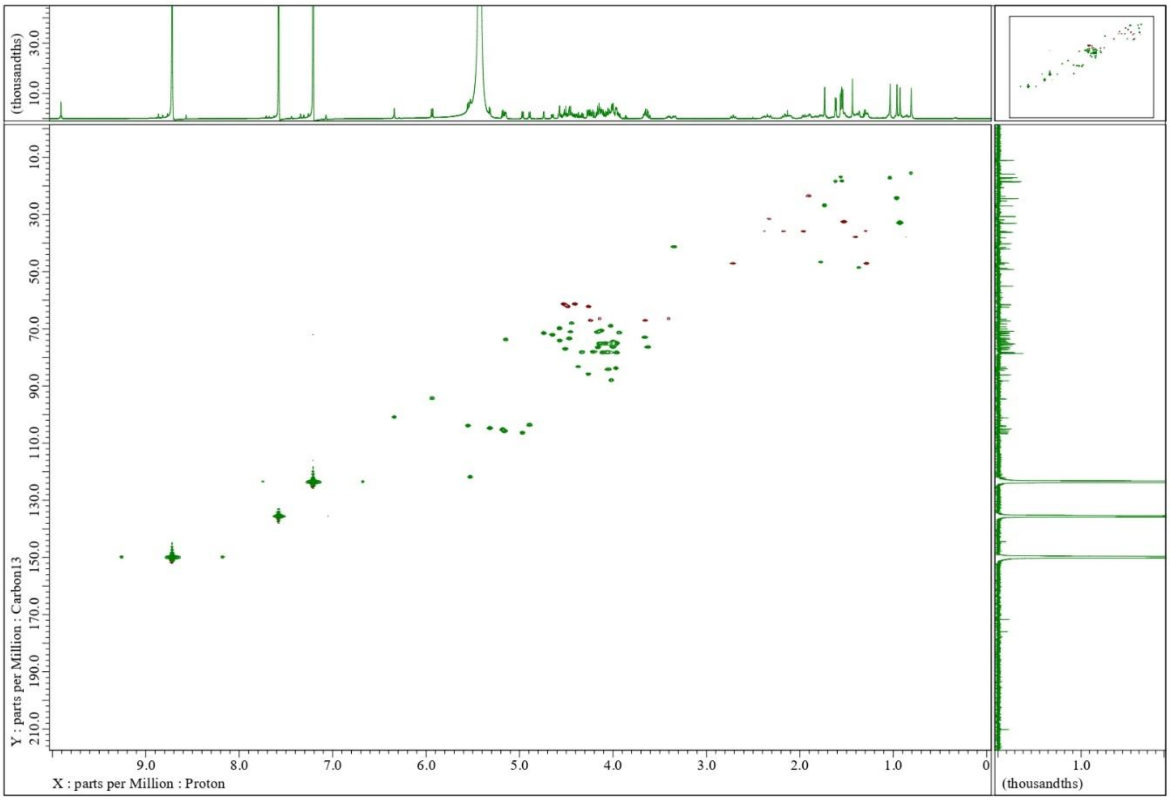

Figure S25. HSQC spectrum of **3**.

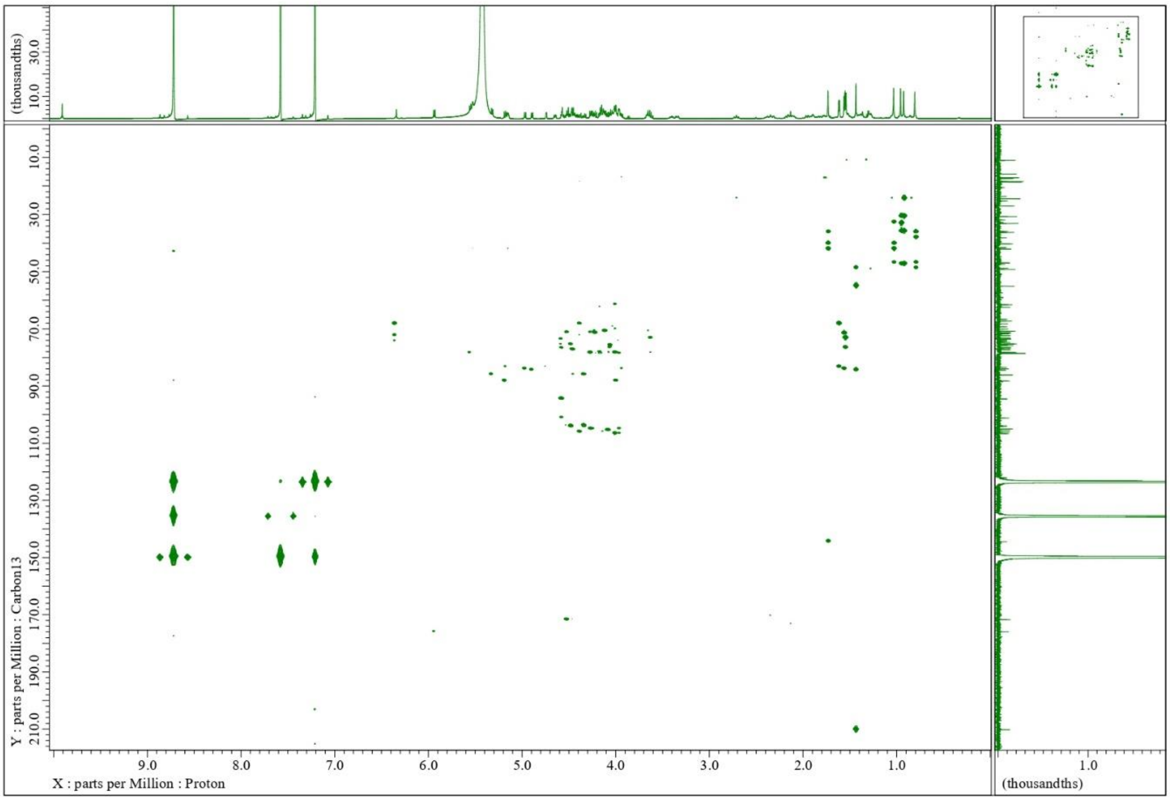

Figure S26. HMBC spectrum of **3**.

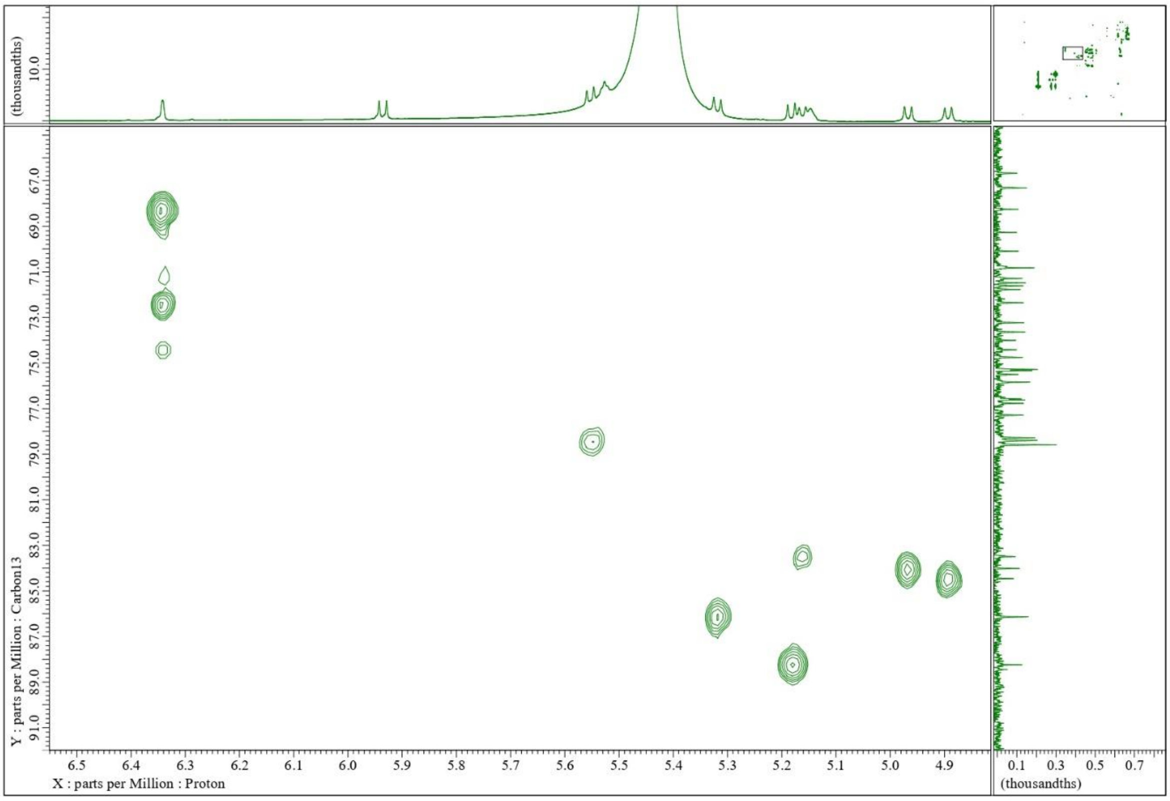

Figure S27. HMBC spectrum of **3** (expanded for anomeric region).

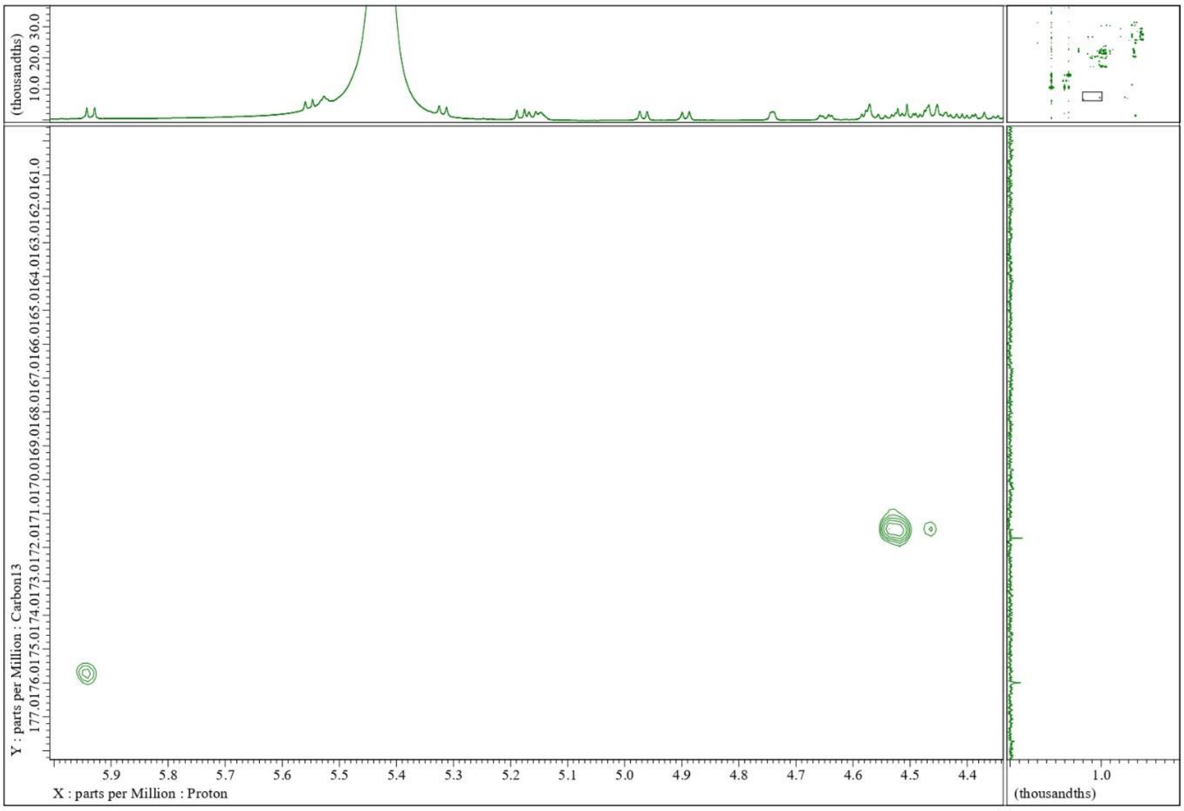

Figure S28. HMBC spectrum of **2** (expanded for between H-1′′′′ of Fuc and C-28 of the aglycone).

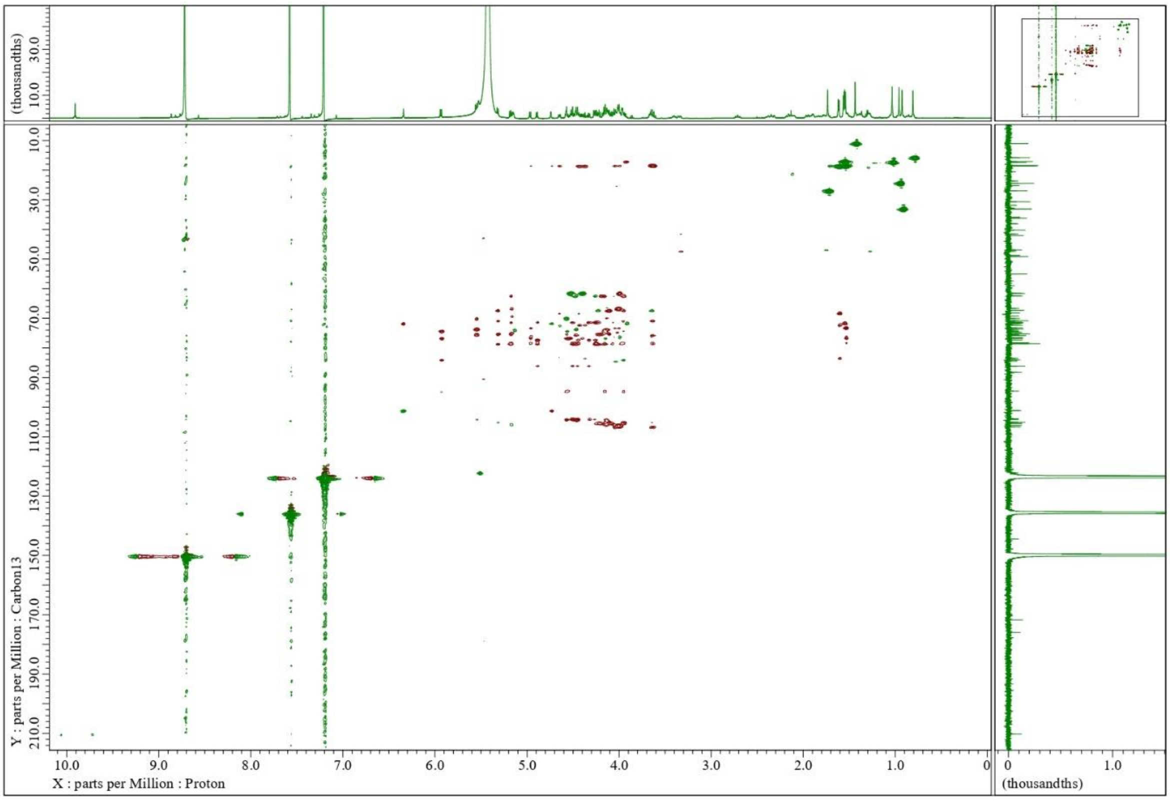

Figure S29. HSQC-TOCSY spectrum of **3**.

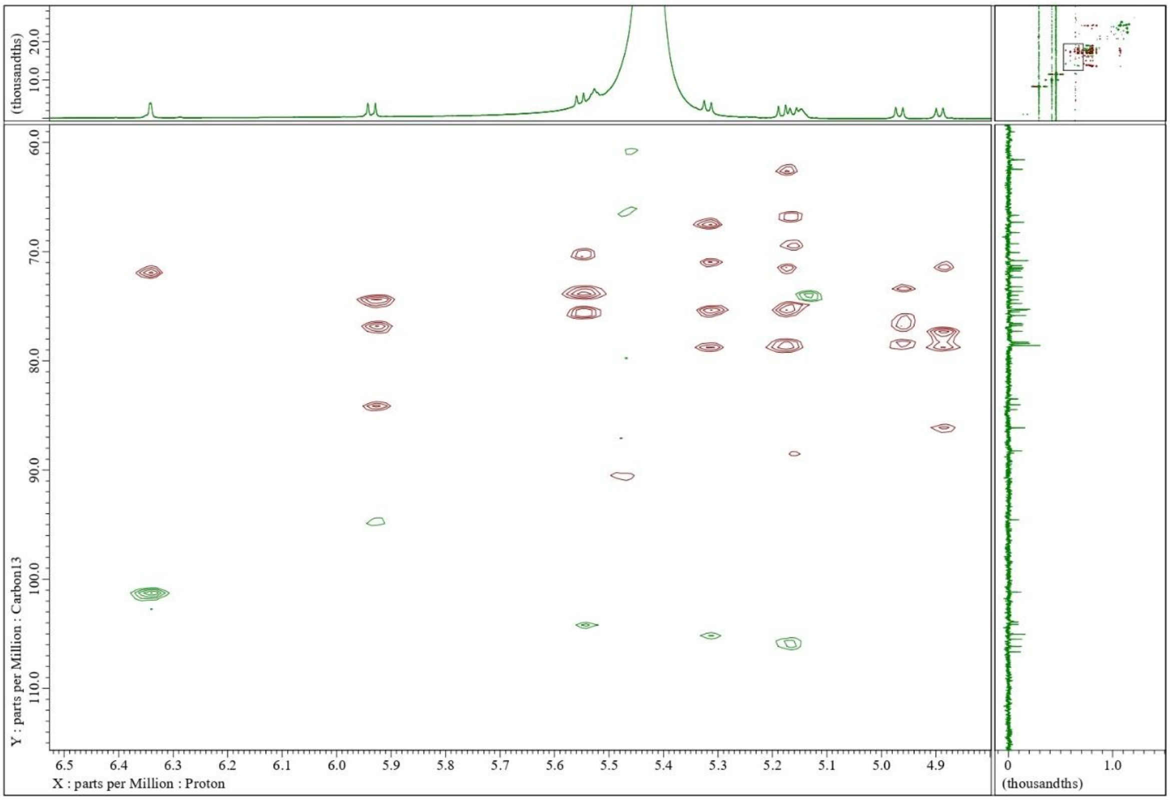

Figure S30. HSQC-TOCSY spectrum of **3** (expanded for anomeric region).

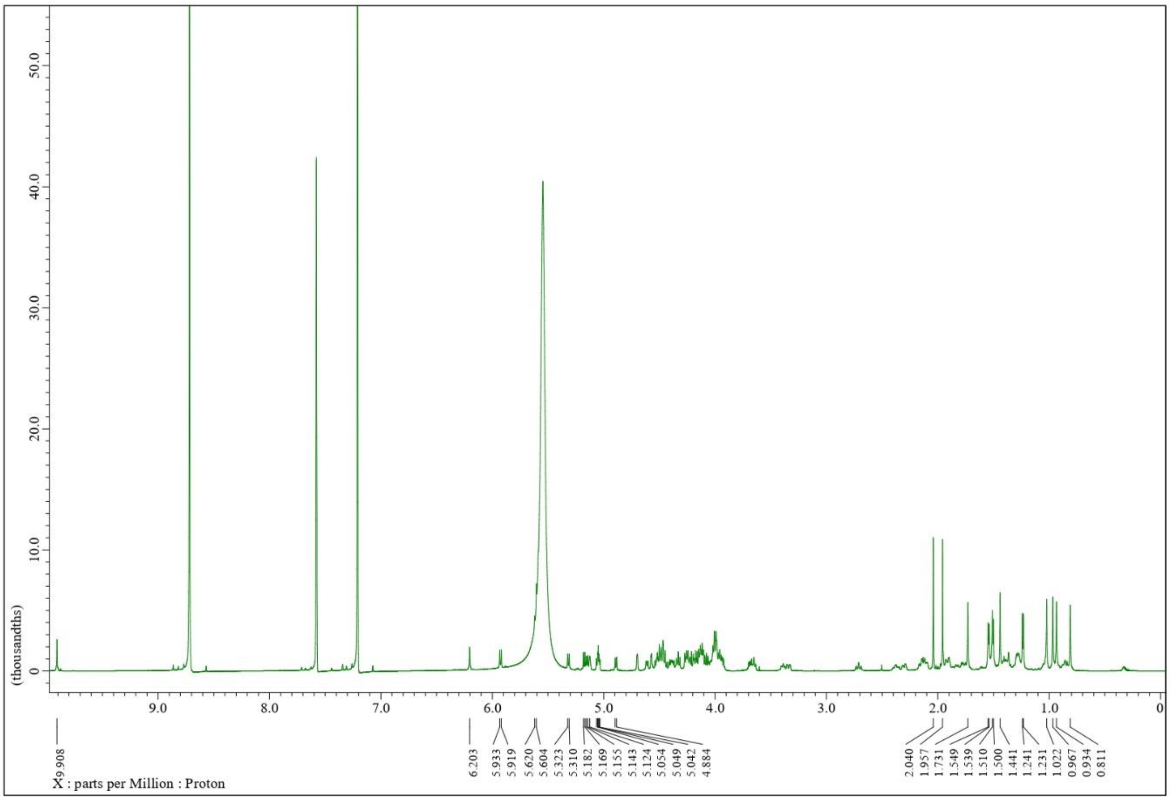

Figure S31. ^{1}H-NMR spectrum of **4**.

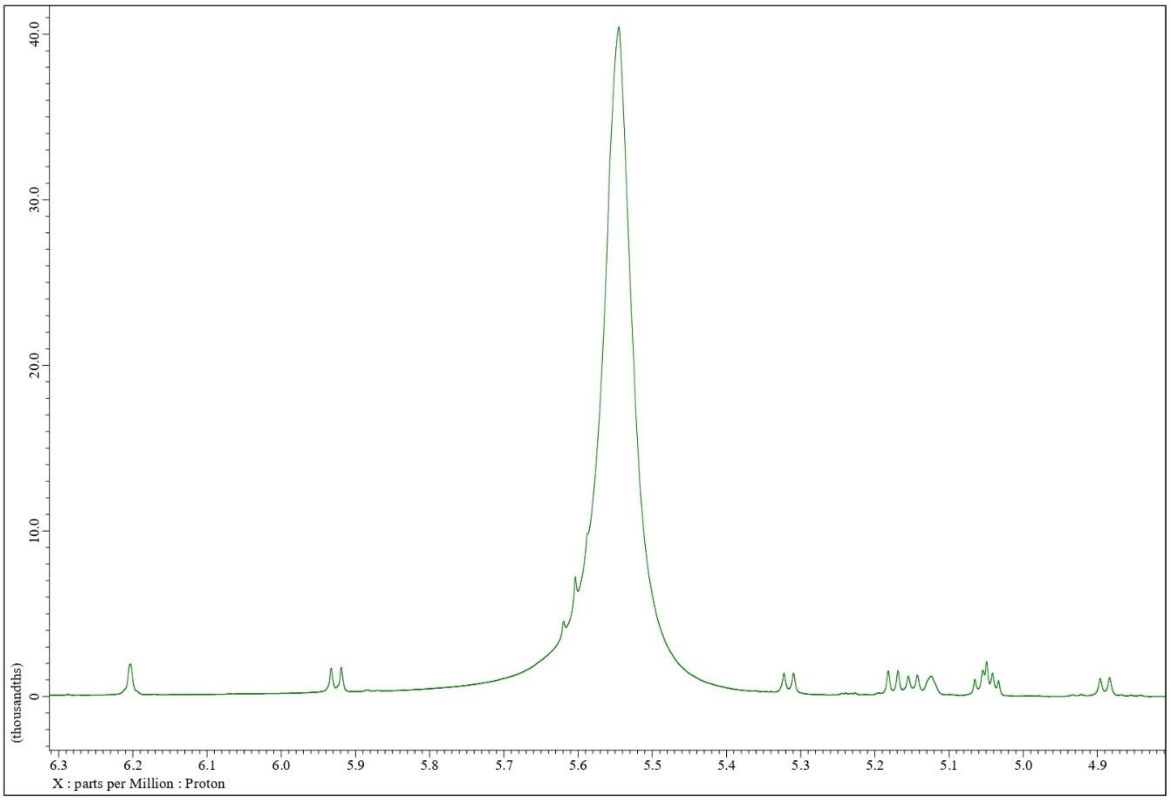

Figure S32. ^{1}H-NMR spectrum of **4** (expanded for anomeric region).

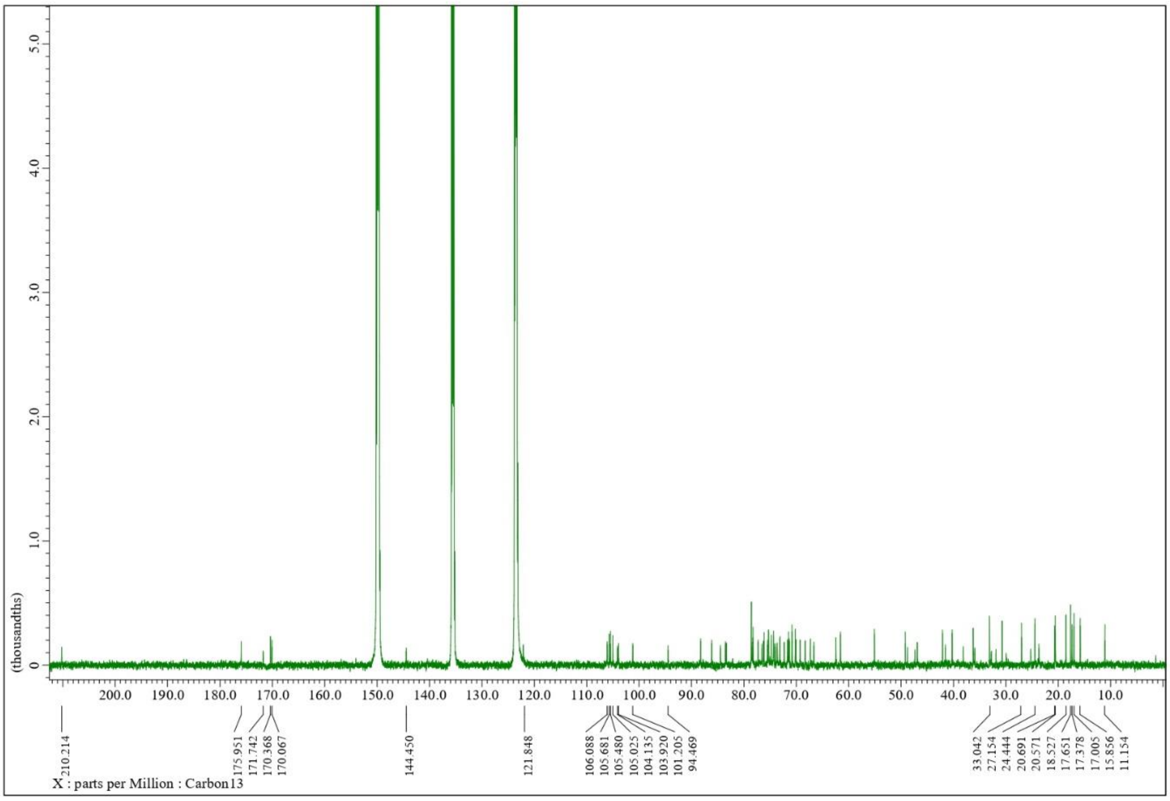

Figure S33. ^{13}C-NMR spectrum of **4**.

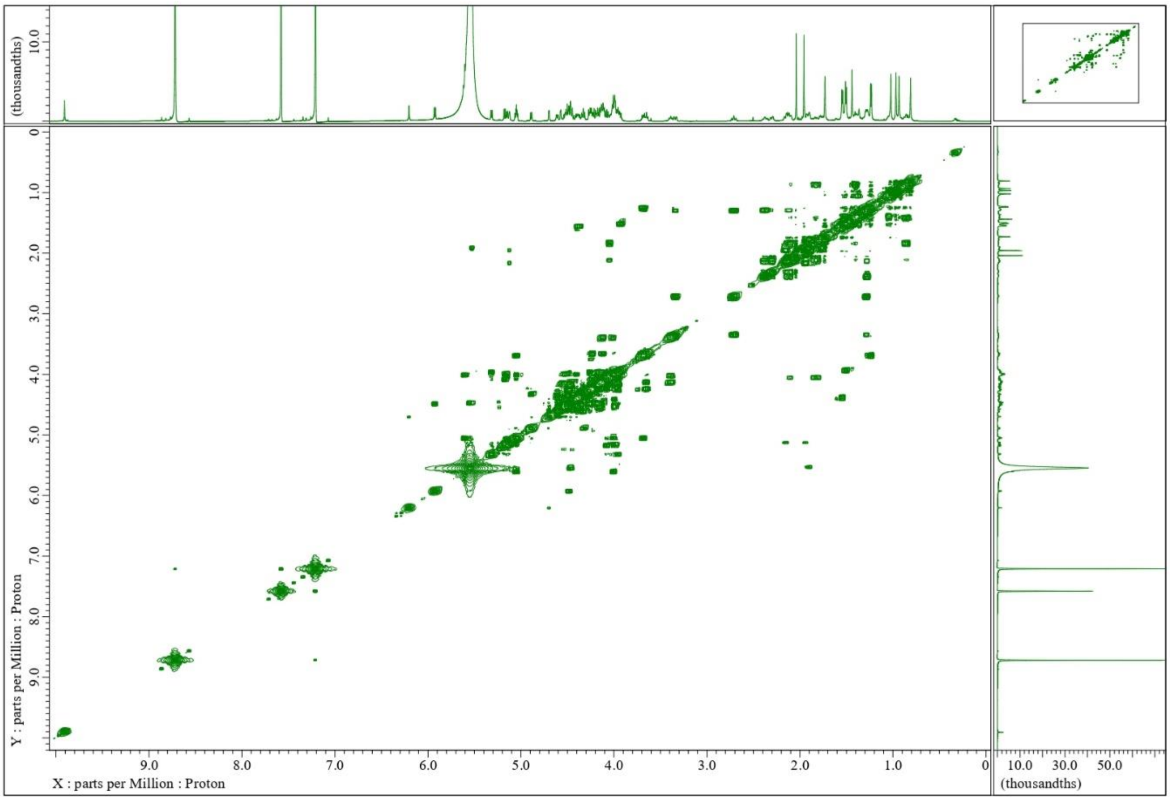

Figure S34. ^{1}H-^{1}H COSY spectrum of **4**.

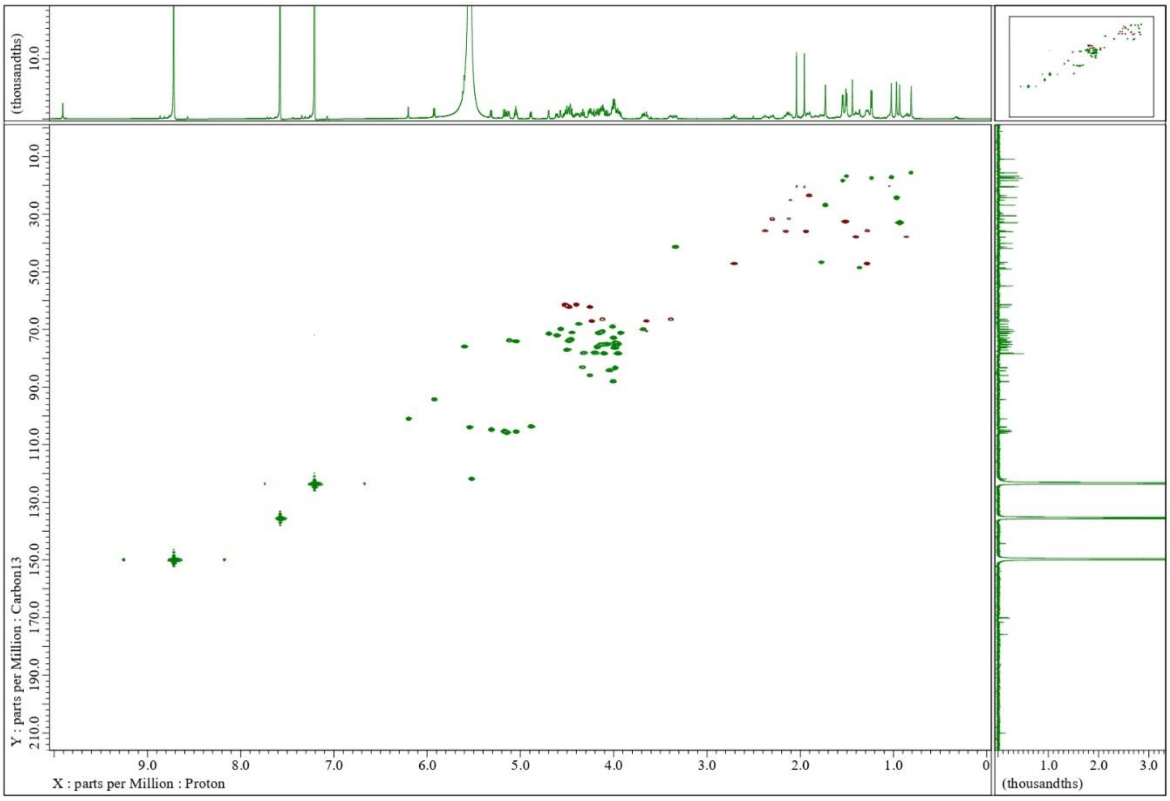

Figure S35. HSQC spectrum of **4**.

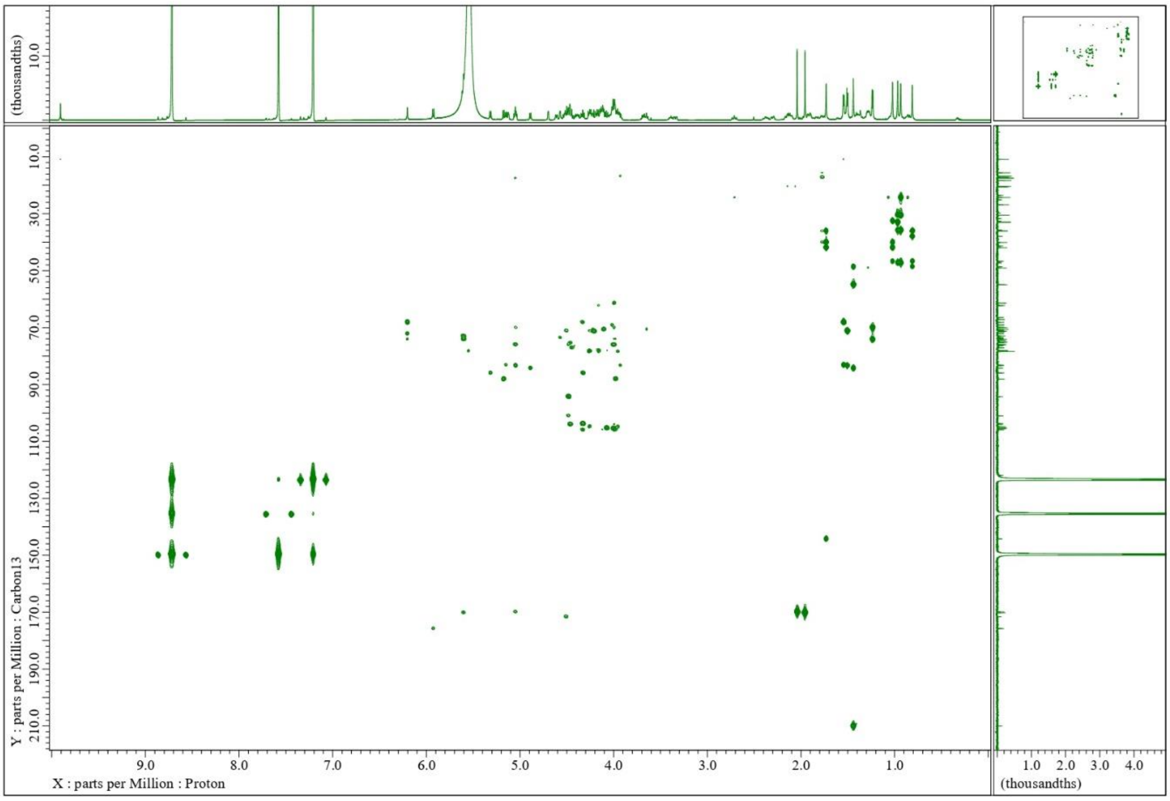

Figure S36. HMBC spectrum of **4**.

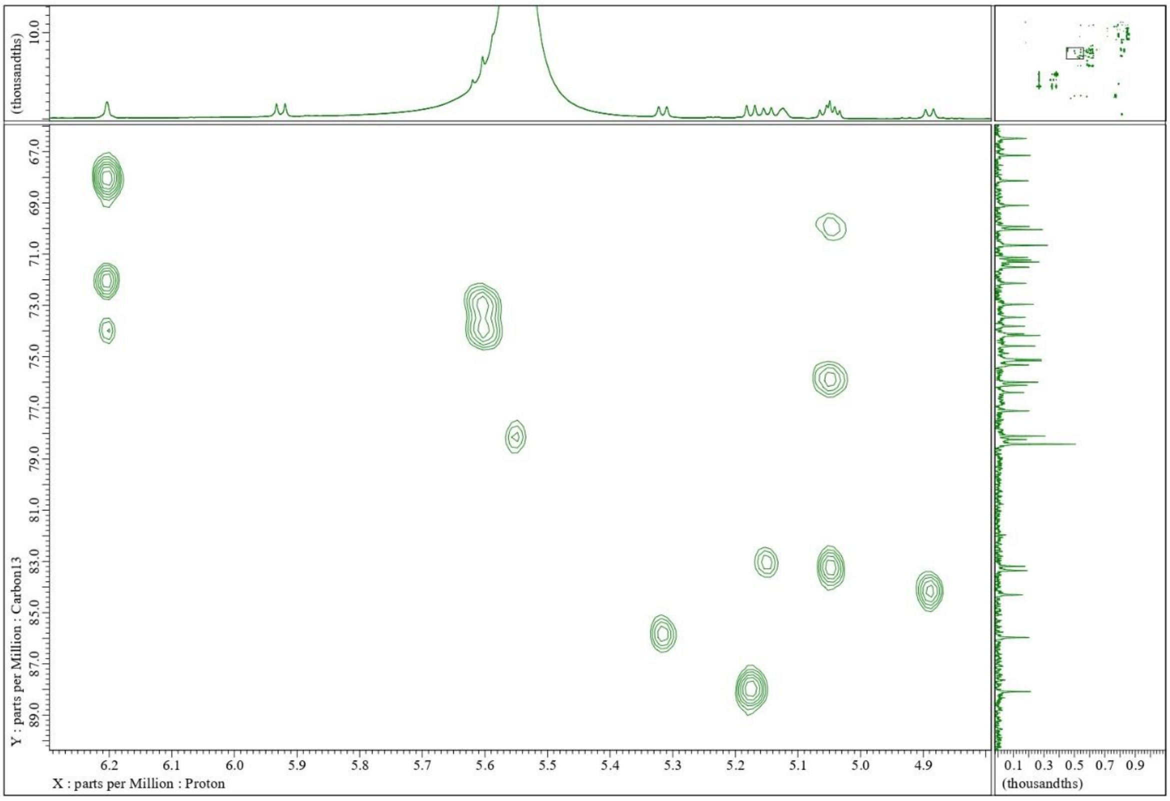

Figure S37. HMBC spectrum of **4** (expanded for anomeric region).

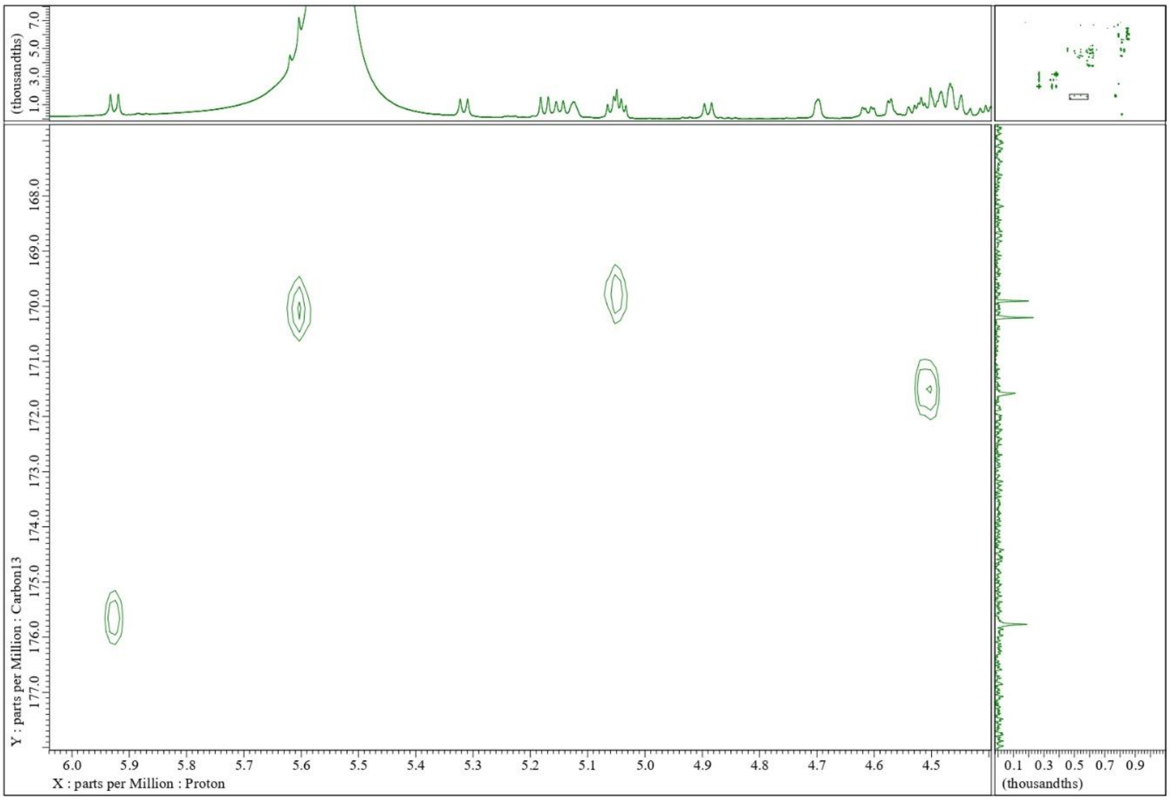

Figure S38. HMBC spectrum of **2** (expanded for between H-1′′′′ of Fuc and C-28 of the aglycone, H-3′′′′′′′′ of Qui and carbonyl carbon of acetyl moiety, and between H-4′′′′′′′′ of Qui and carbonyl carbon of acetyl moiety).

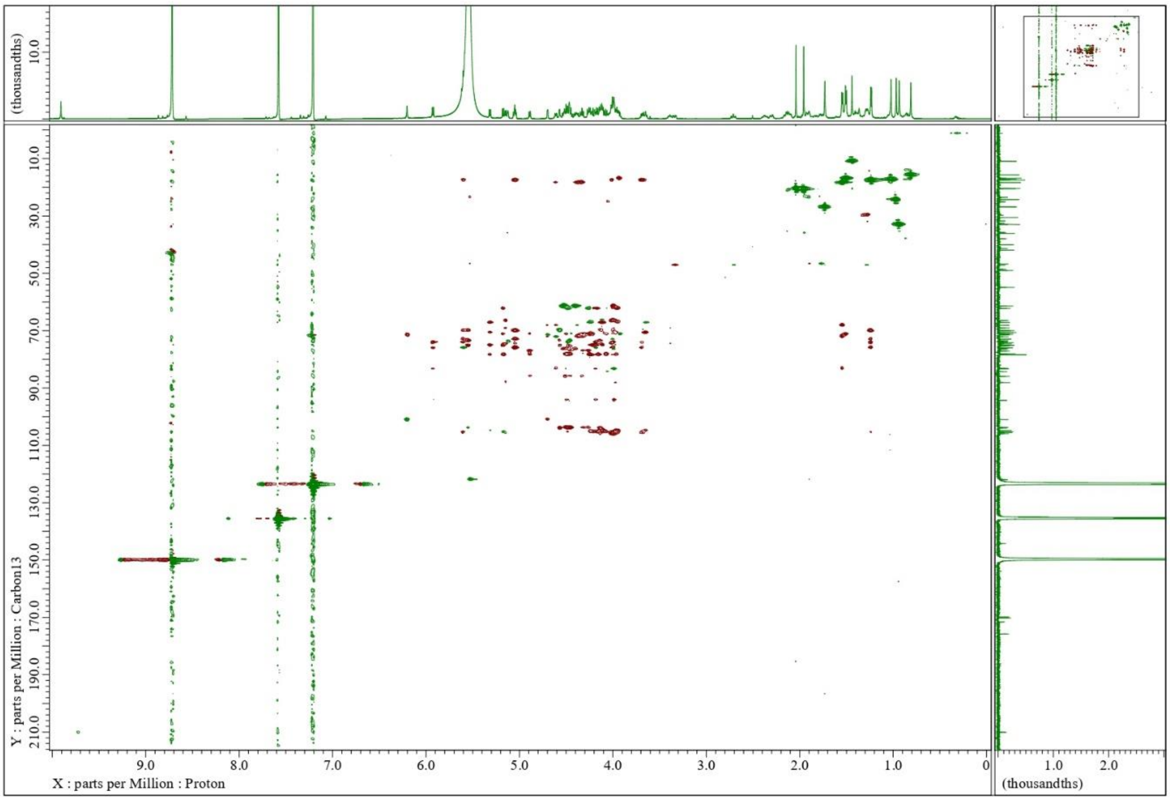

Figure S39. HSQC-TOCSY spectrum of **4**.

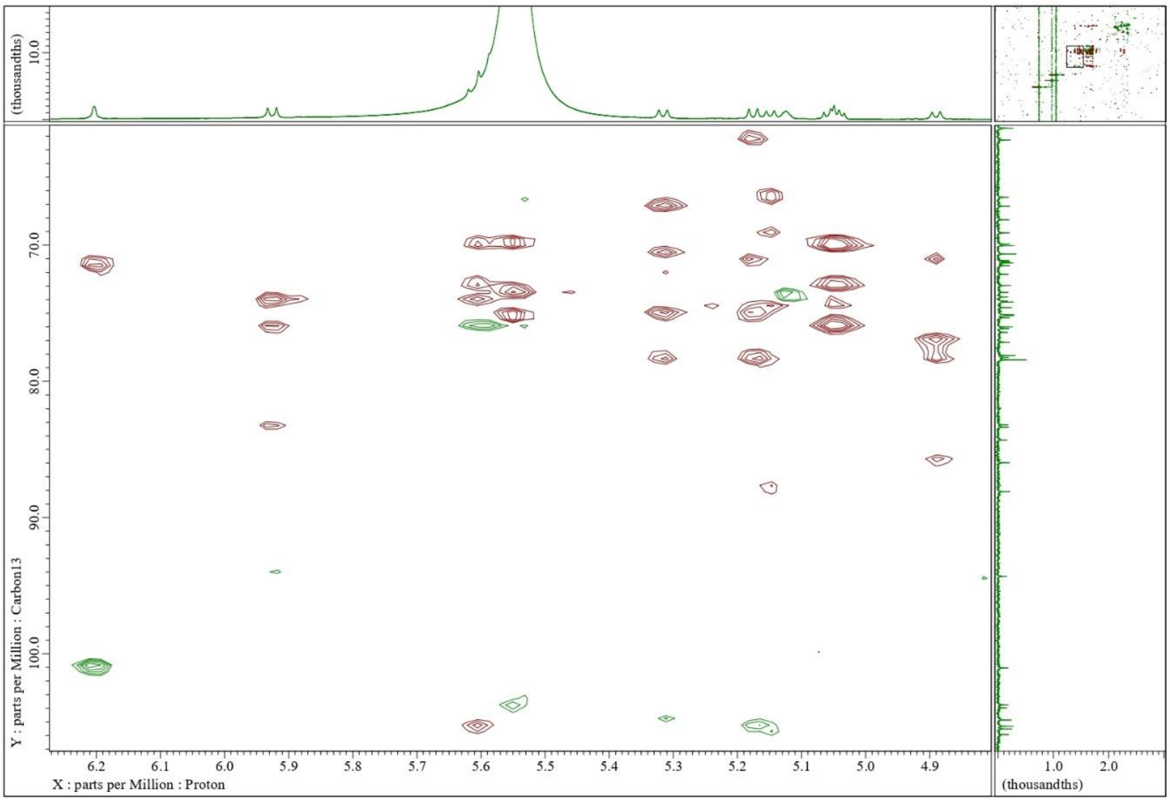

Figure S40. HSQC-TOCSY spectrum of **4** (expanded for anomeric region).

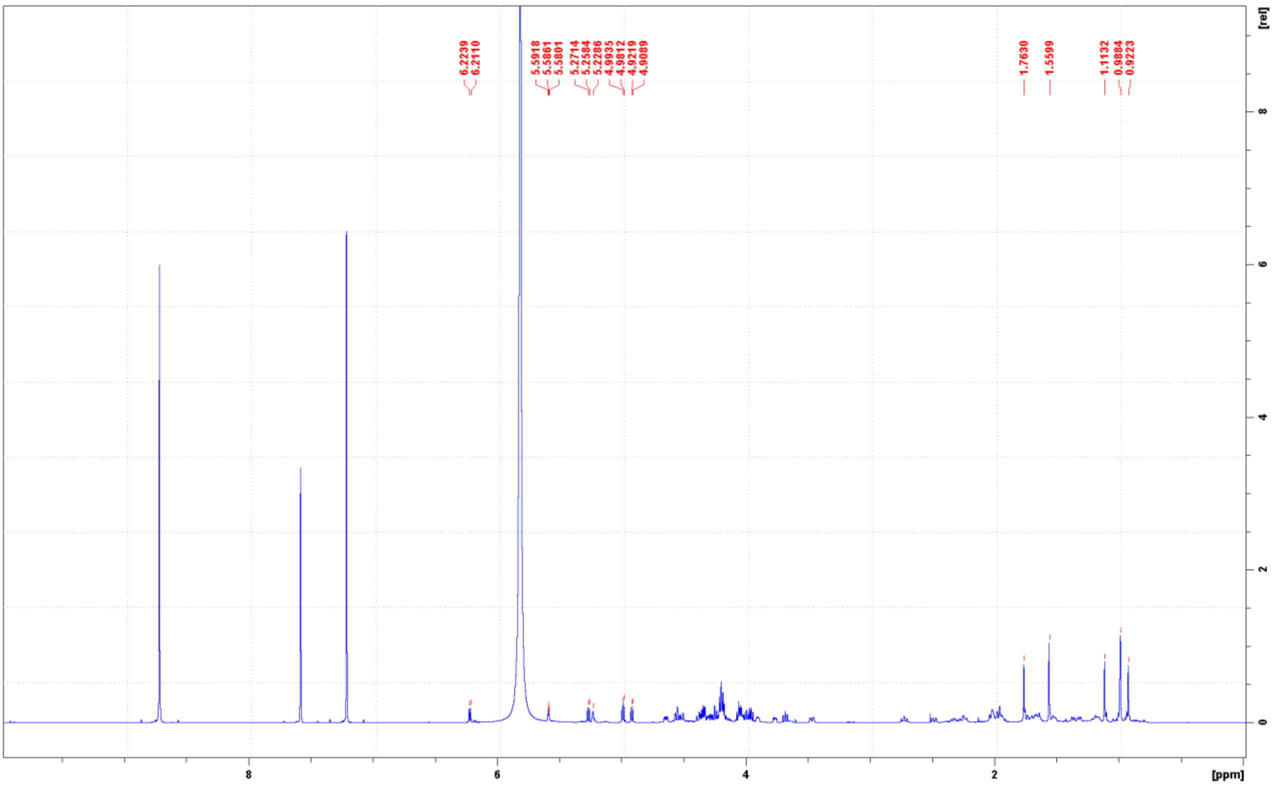

Figure S41. ^{1}H-NMR spectrum of **5**.

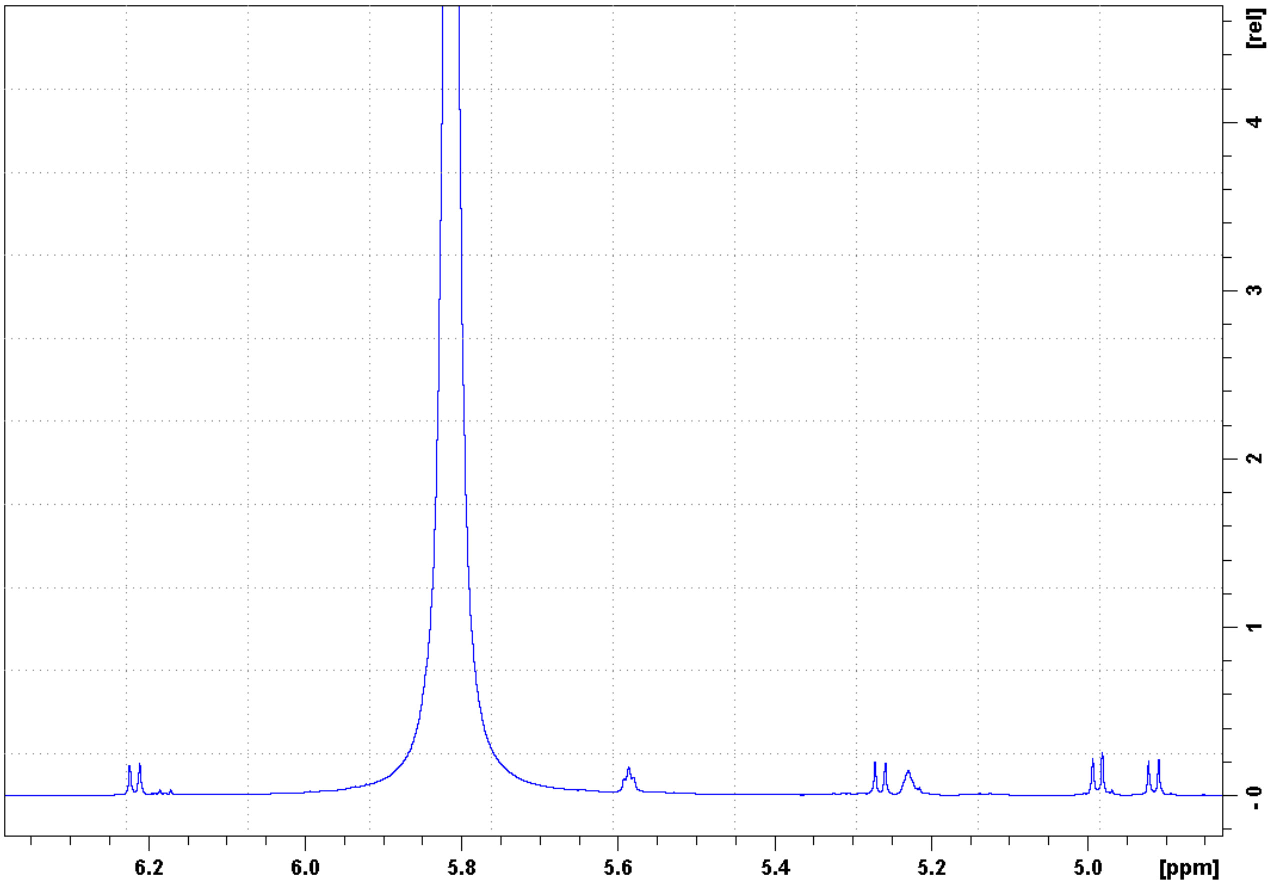

Figure S42. ^{1}H-NMR spectrum of **5** (expanded for anomeric region).

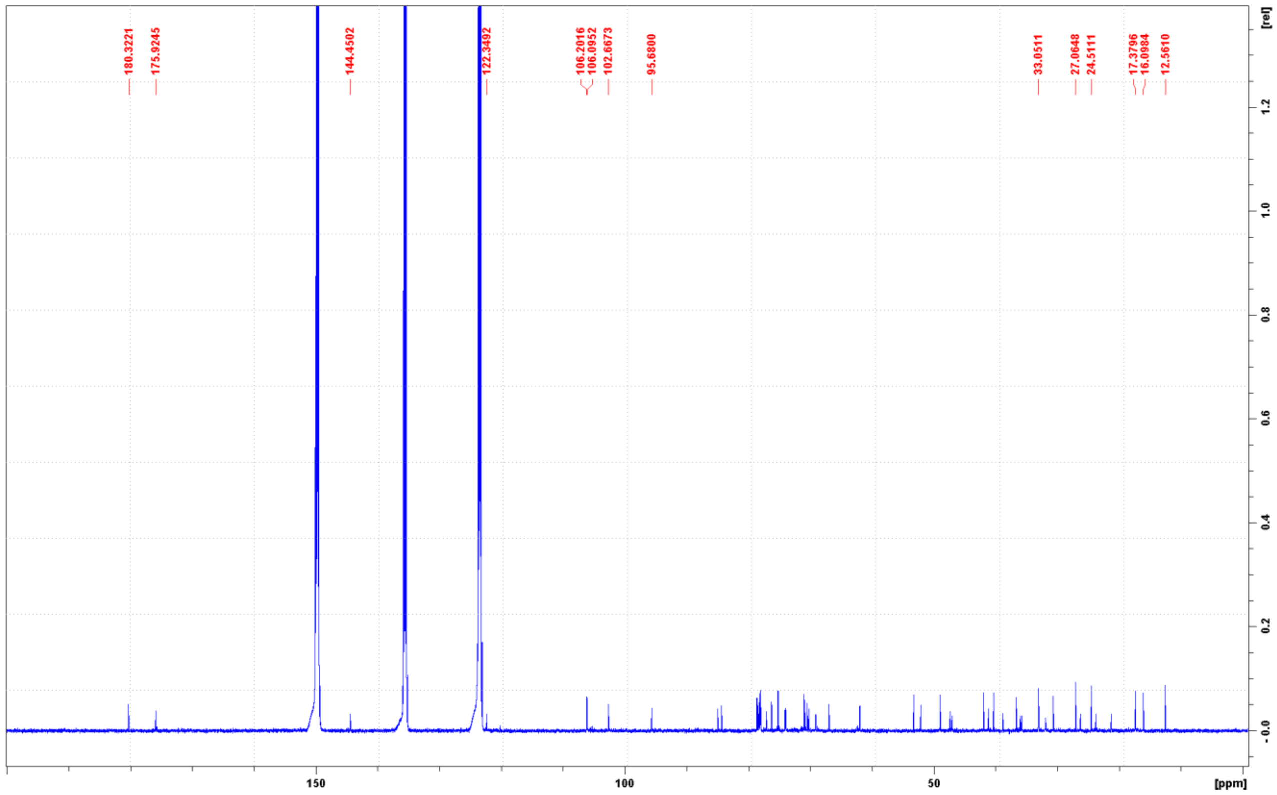

Figure S43. ^{13}C-NMR spectrum of **5**.

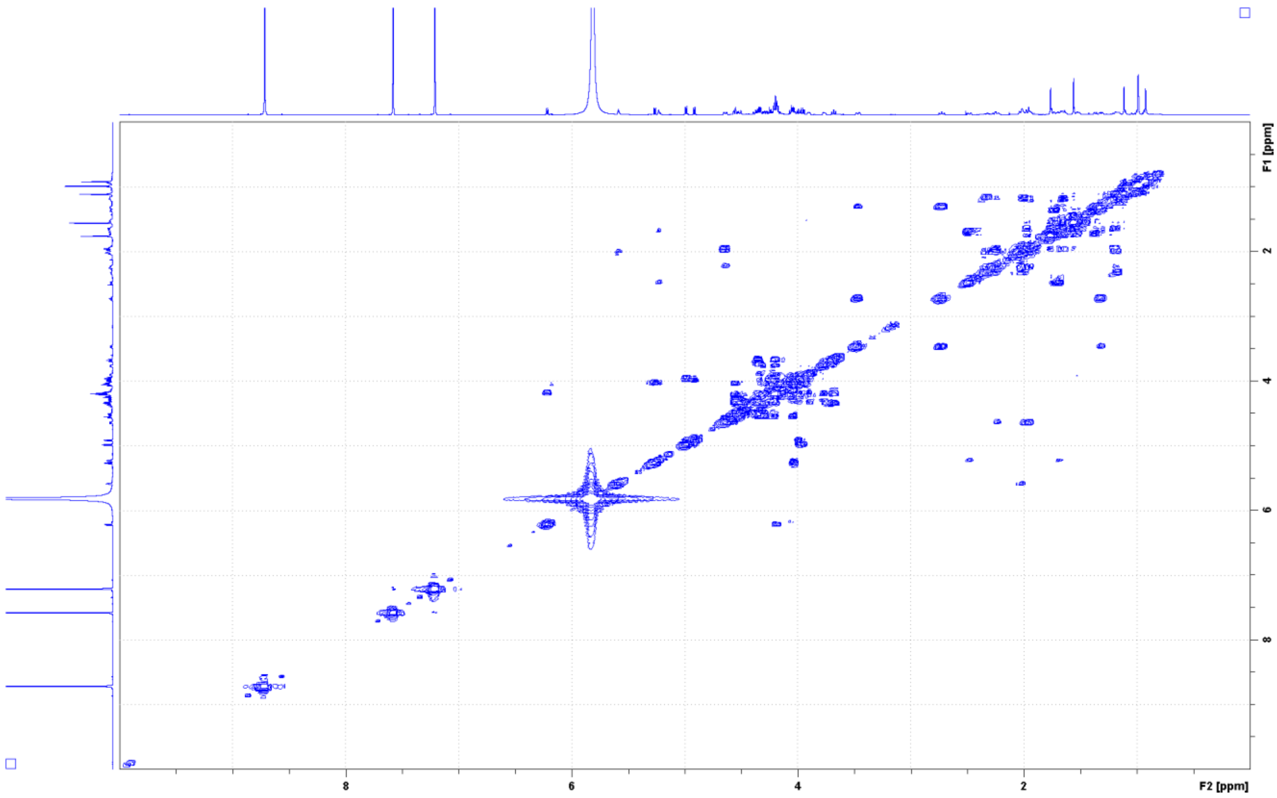

Figure S44. ^{1}H-^{1}H COSY spectrum of **5**.

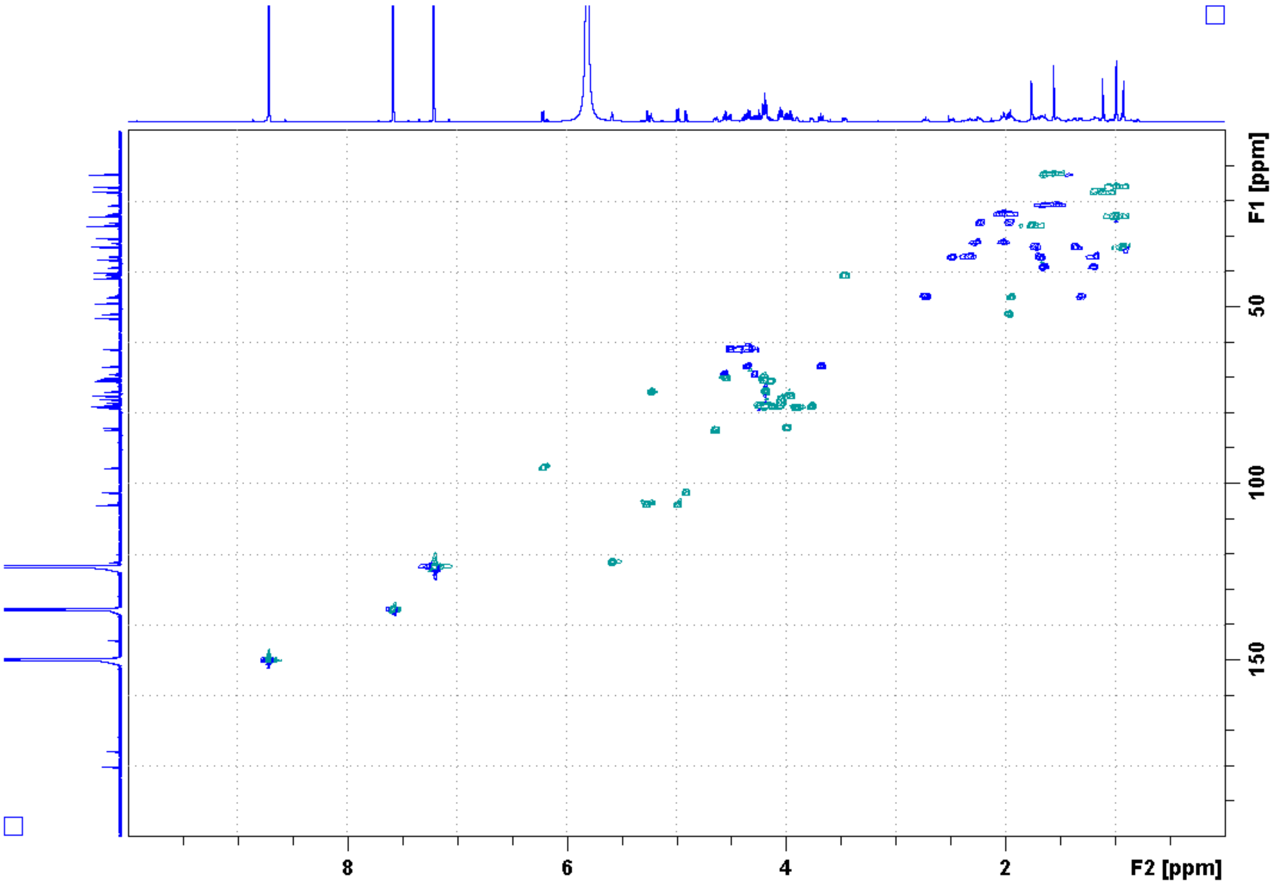

Figure S45. HSQC spectrum of **5**.

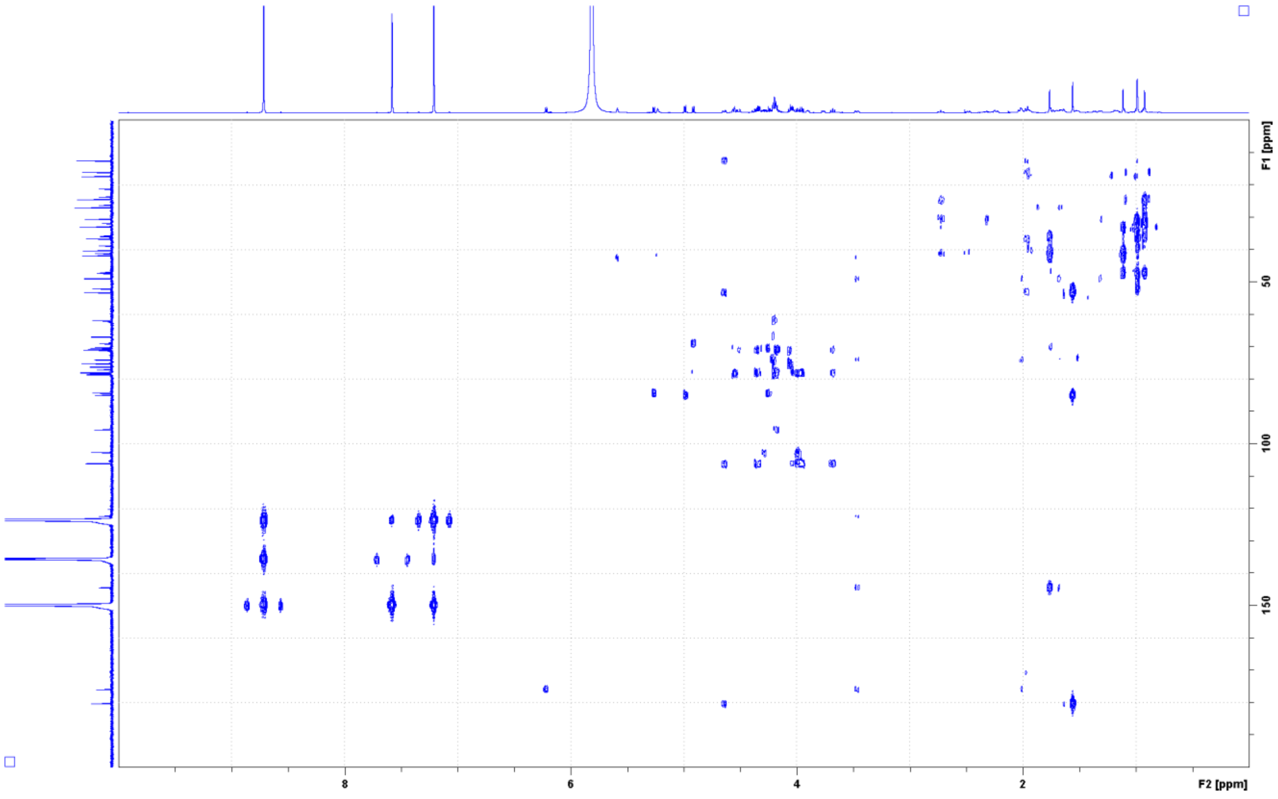

Figure S46. HMBC spectrum of **5**.

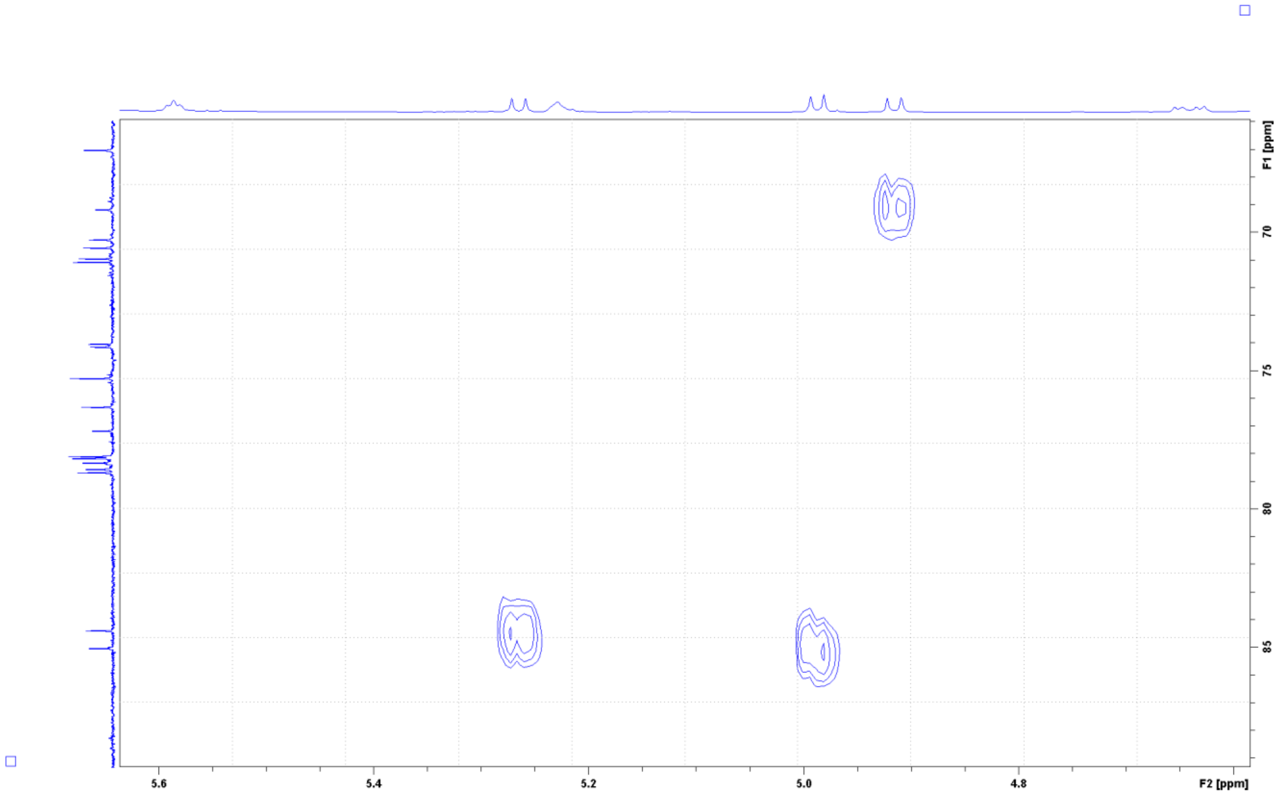

Figure S47. HMBC spectrum of **5** (expanded for anomeric region).

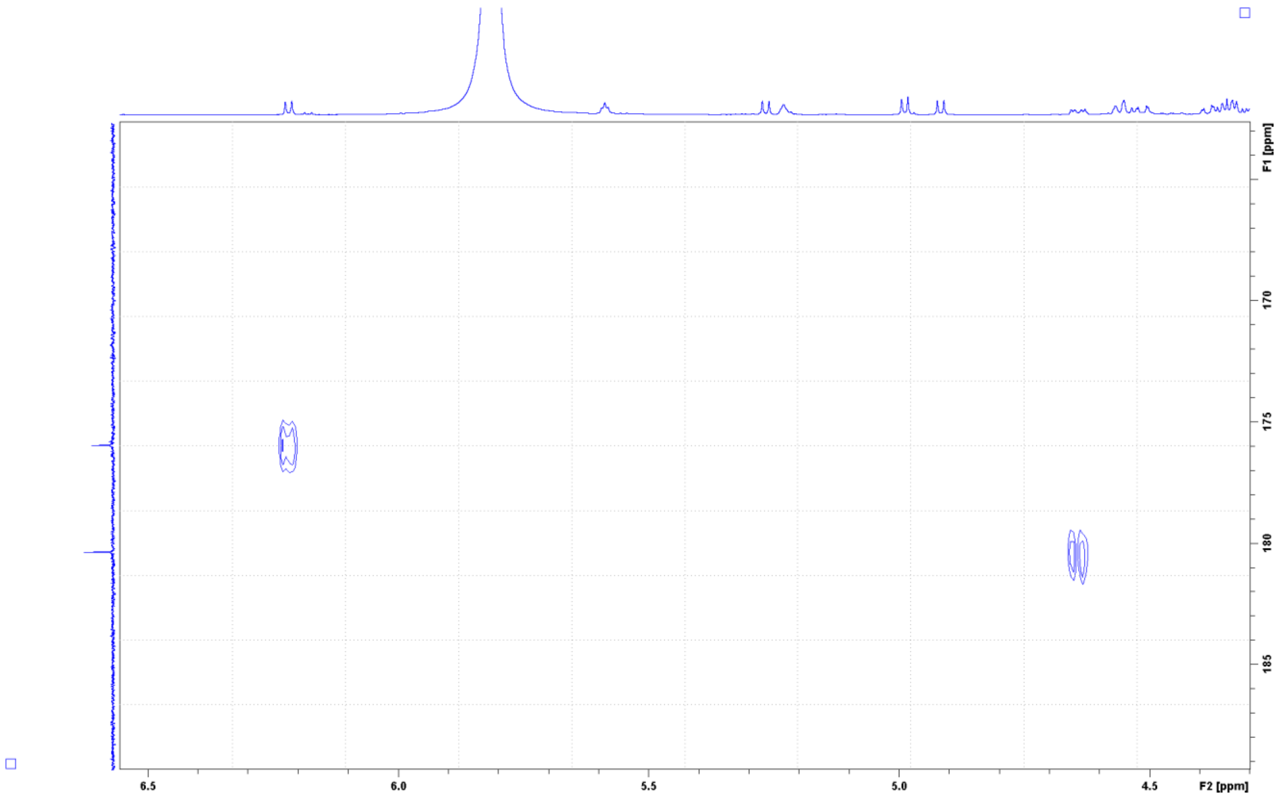

Figure S48. HMBC spectrum of **5** (expanded for between H-1′′ of Glc (I) and C-28 of the aglycone).

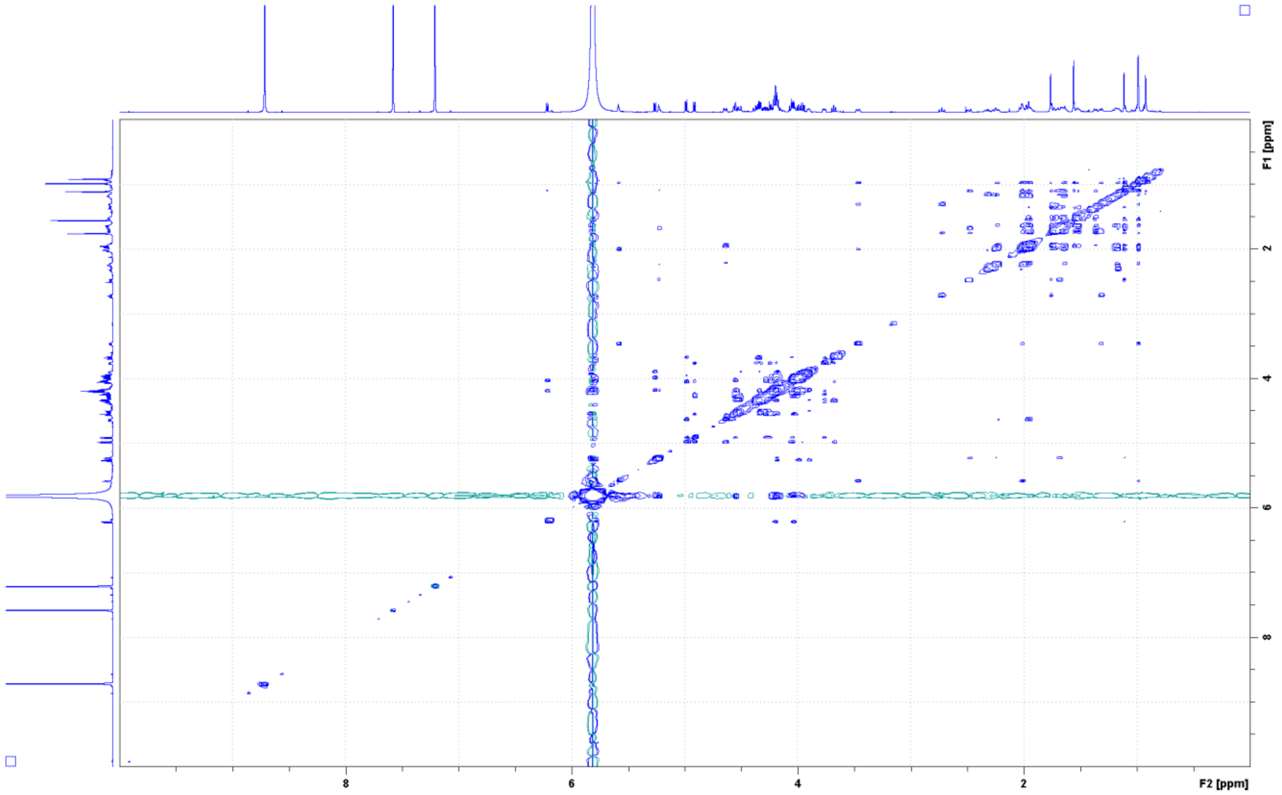

Figure S49. NOESY spectrum of **5**.

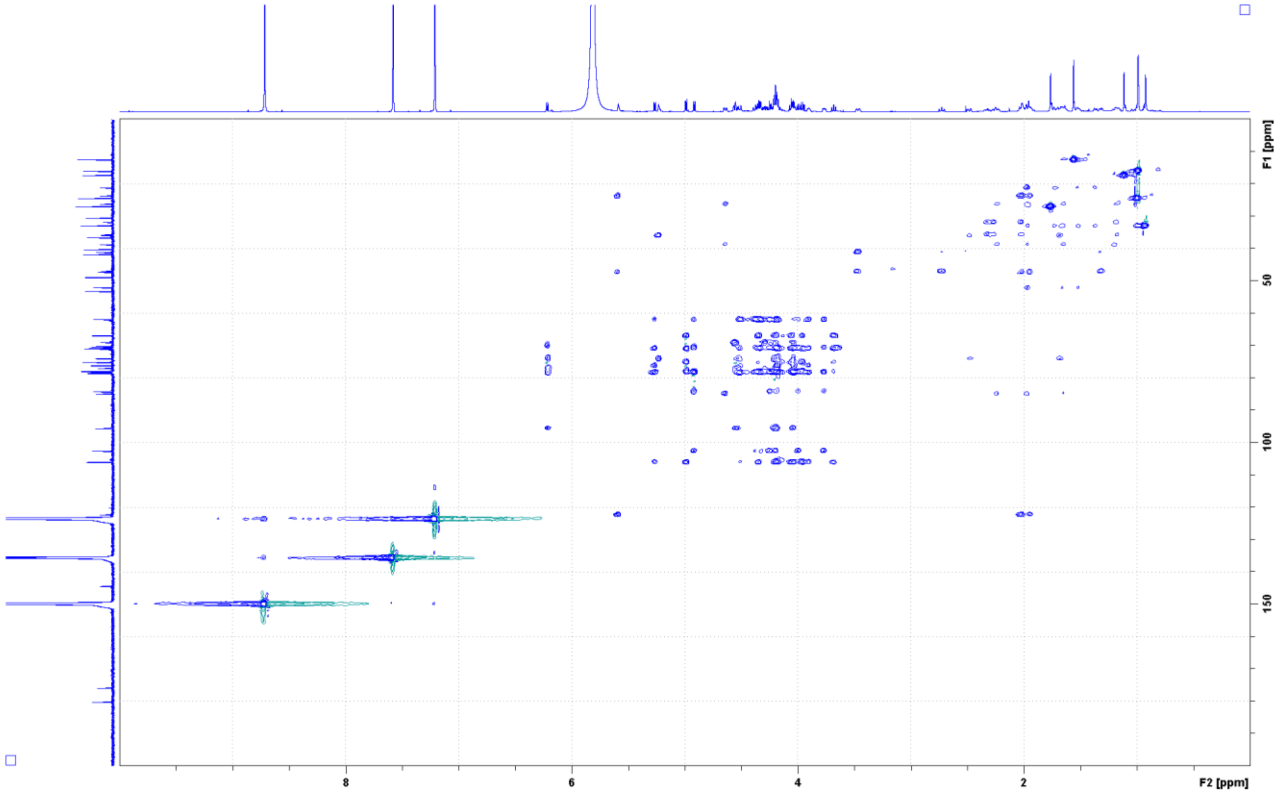

Figure S50. HSQC-TOCSY spectrum of **5**.

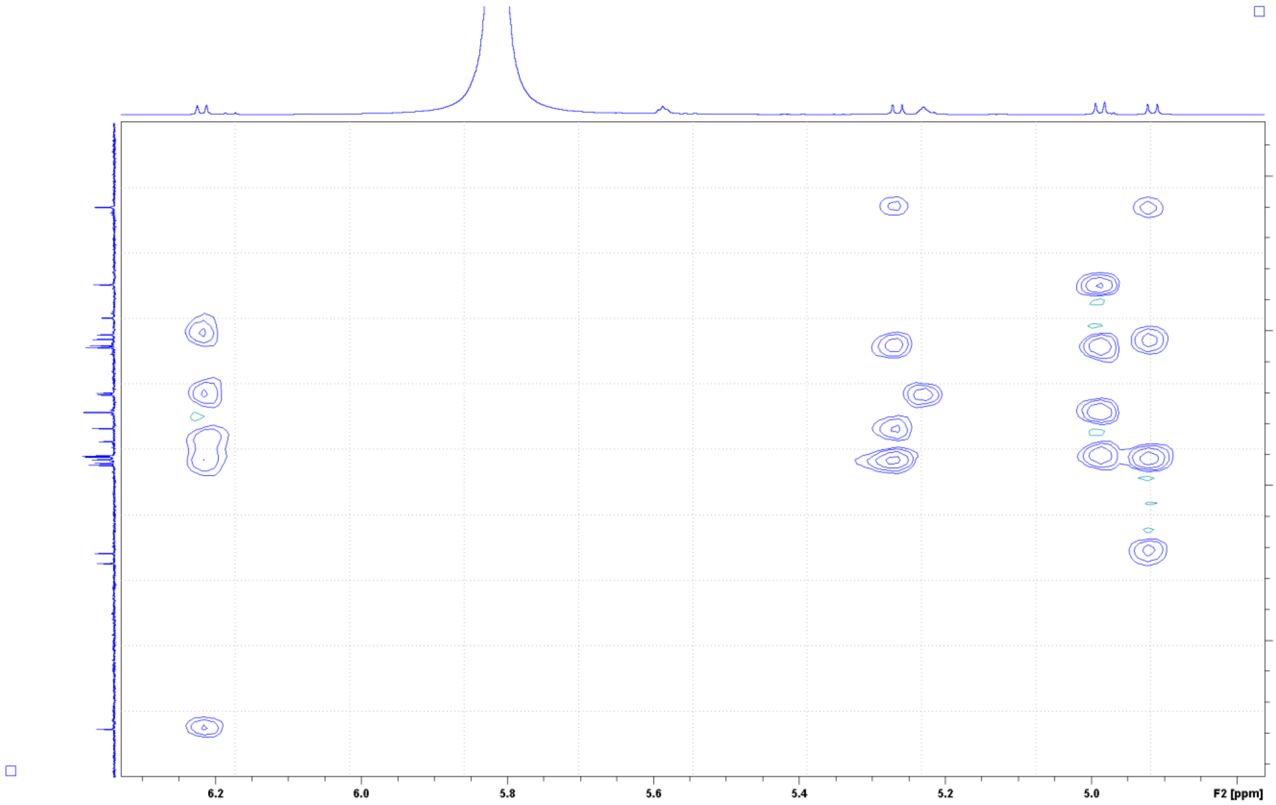

Figure S51. HSQC-TOCSY spectrum of **5** (expanded for anomeric region).

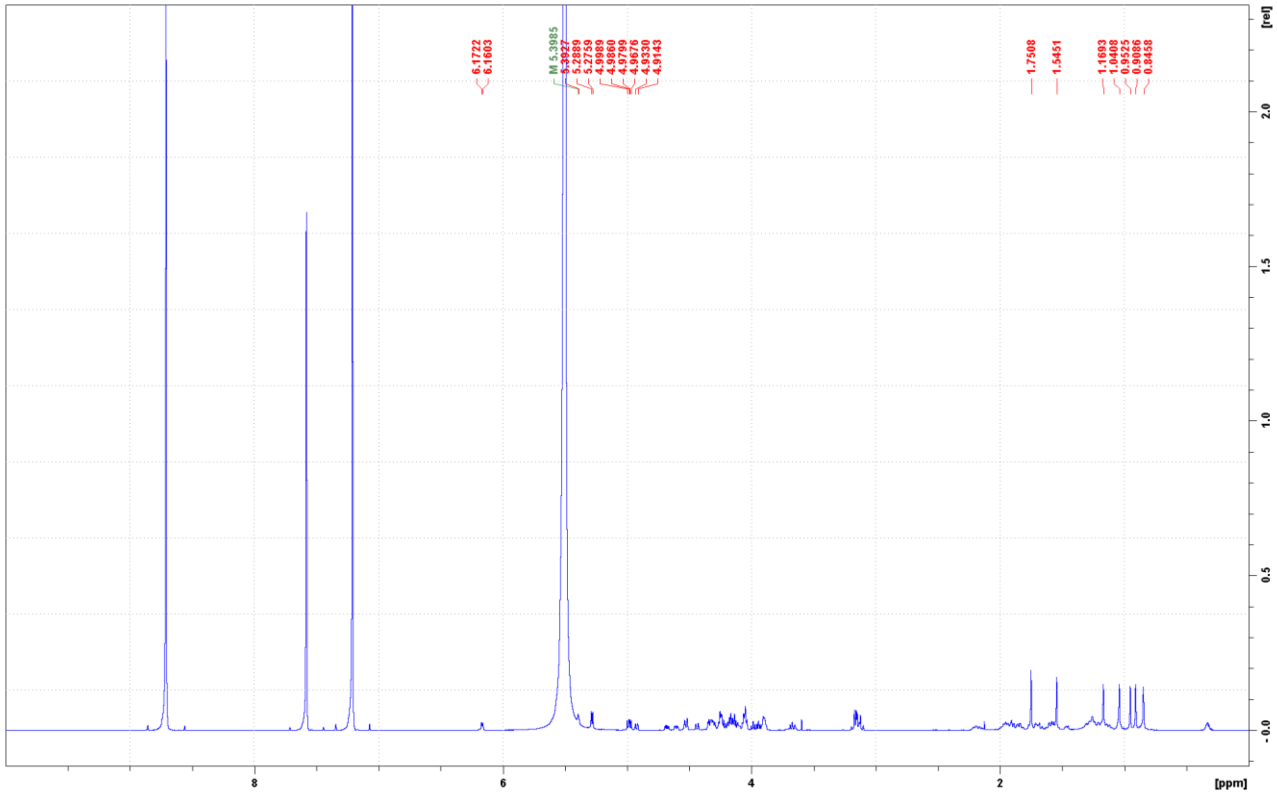

Figure S52. ^{1}H-NMR spectrum of **6**.

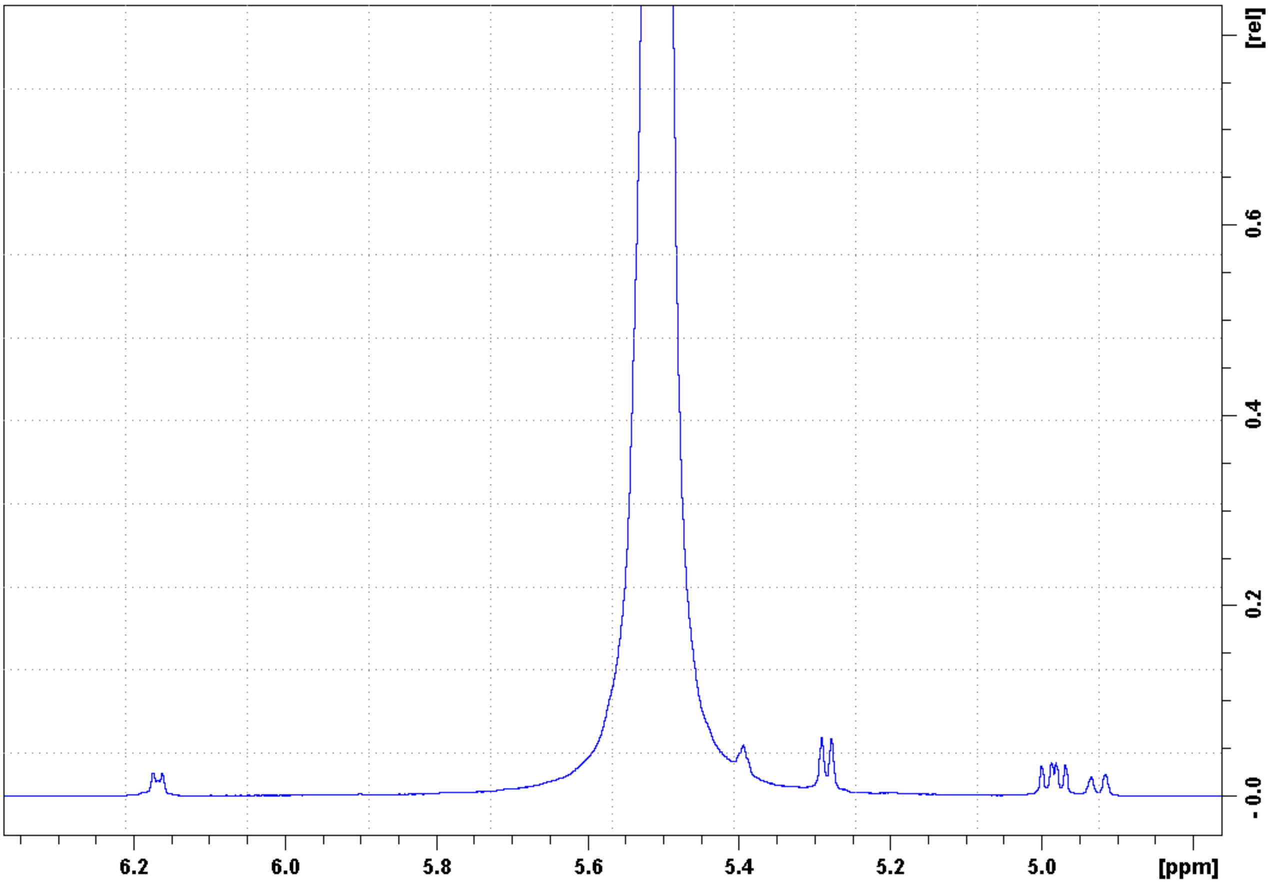

Figure S53. ^{1}H-NMR spectrum of **6** (expanded for anomeric region).

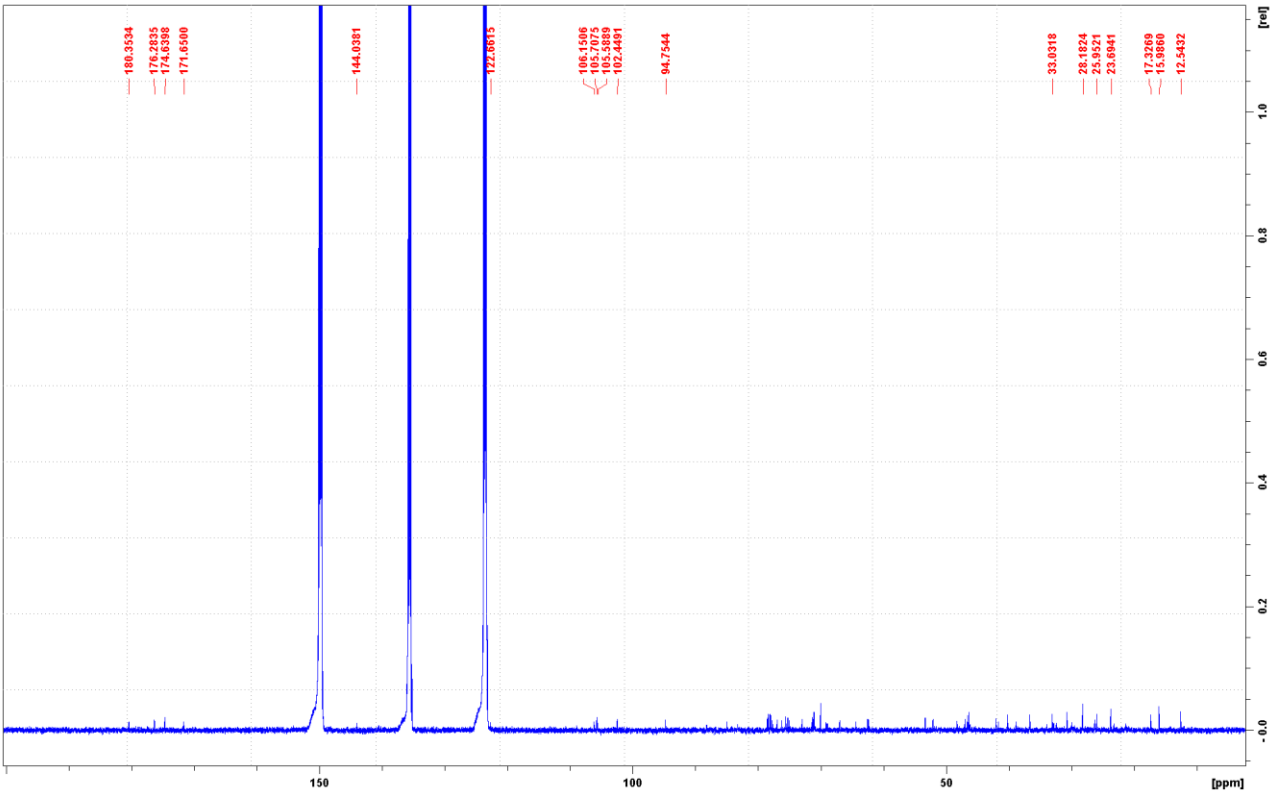

Figure S54. ^{13}C-NMR spectrum of **6**.

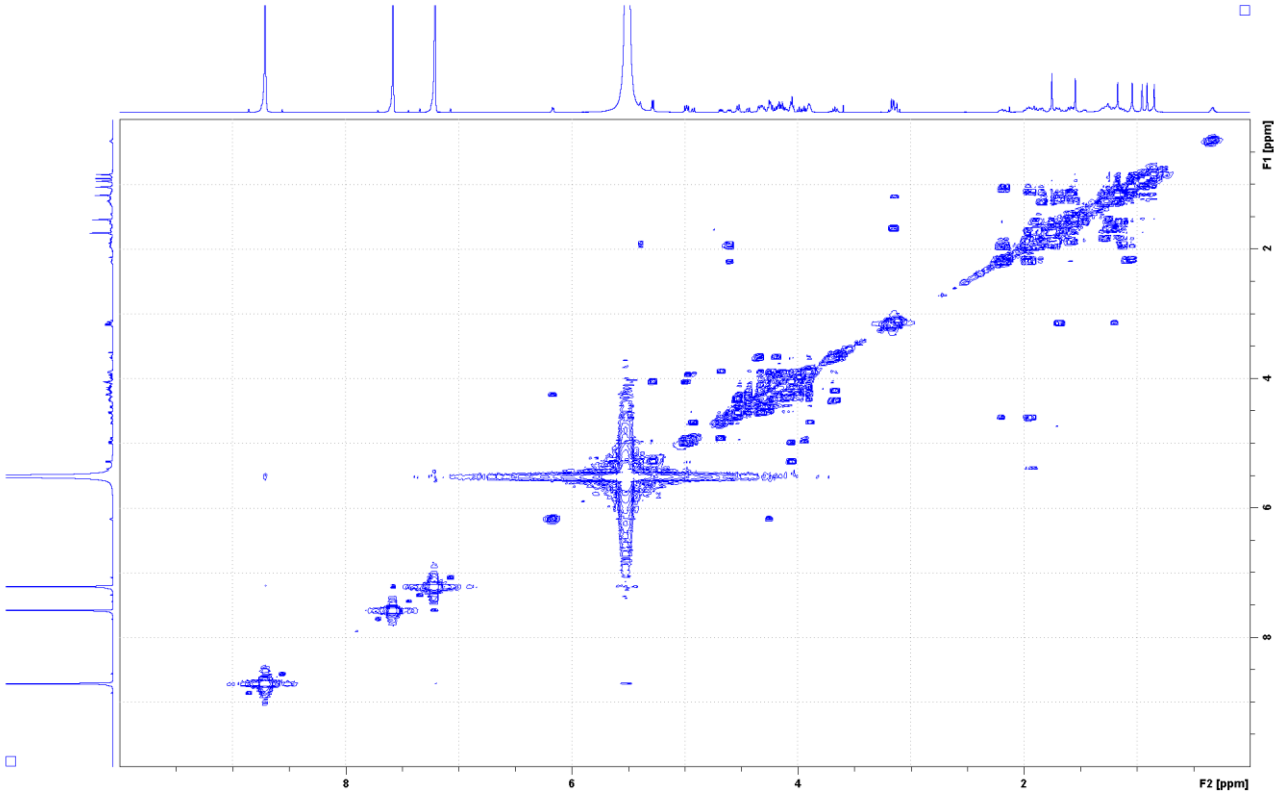

Figure S55. ^{1}H-^{1}H COSY spectrum of **6**.

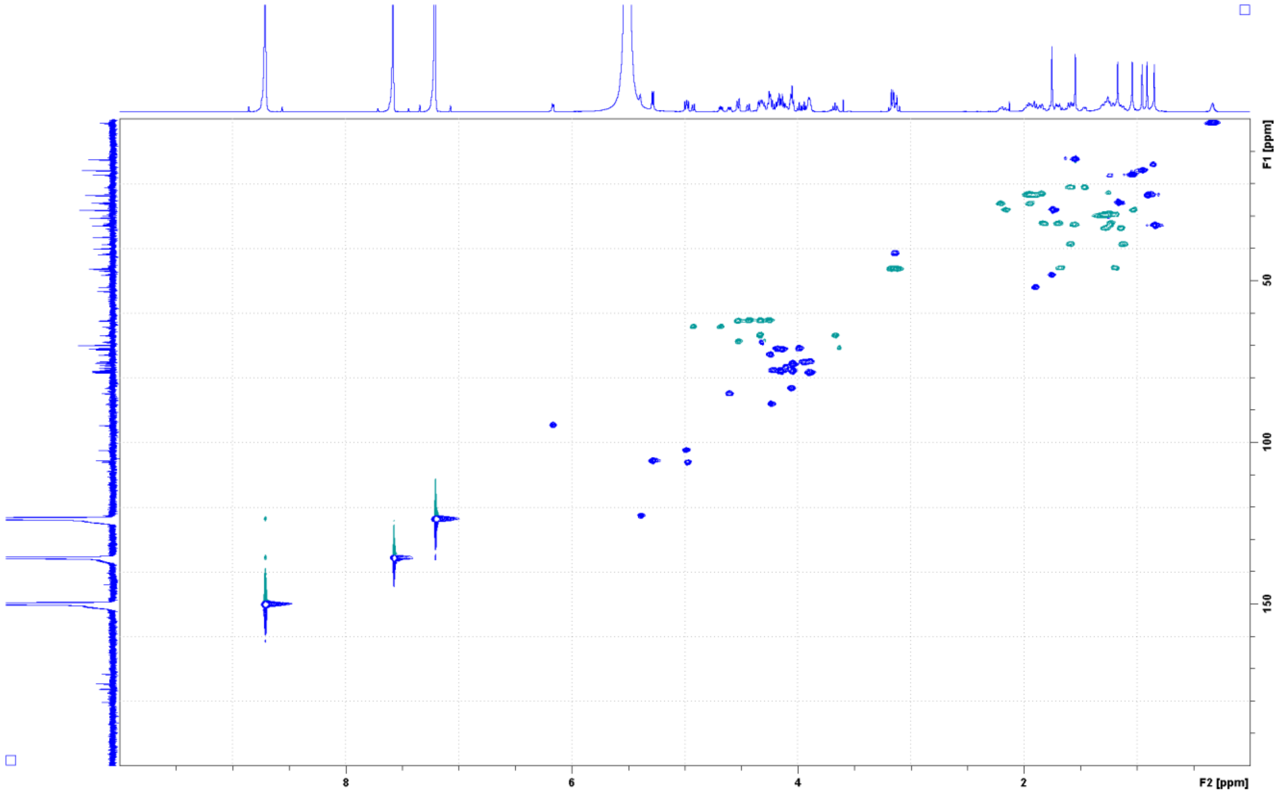

Figure S56. HSQC spectrum of **6**.

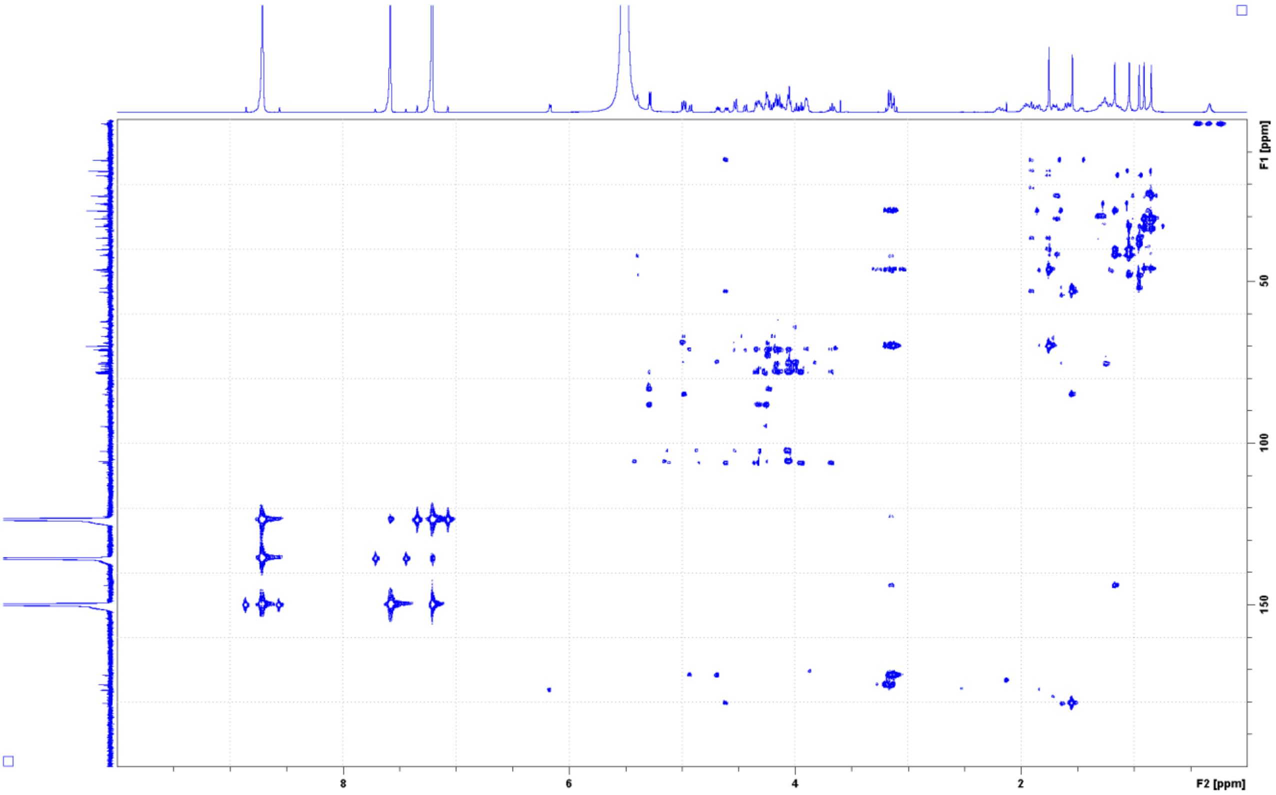

Figure S57. HMBC spectrum of **6**.

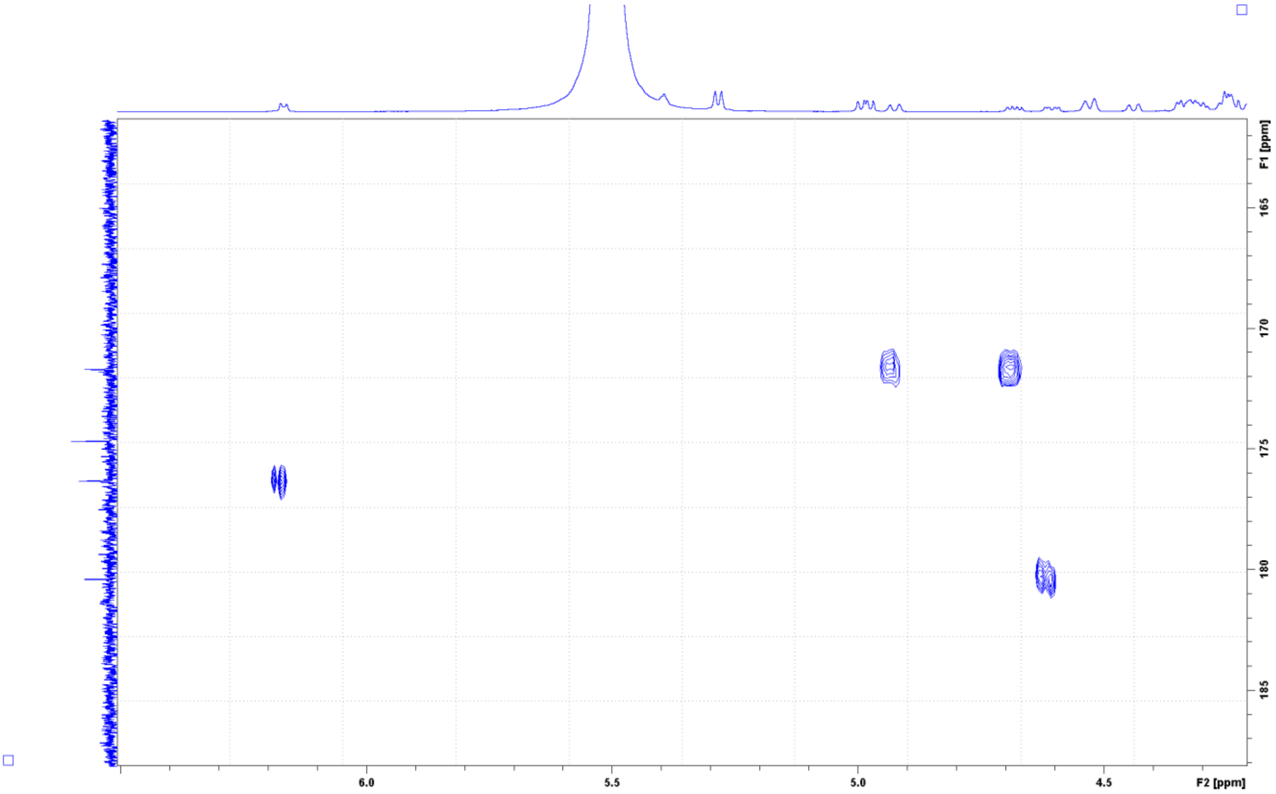

Figure S58. HMBC spectrum of **6** (expanded for between H-1′′ of Glc (I) and C-28 of the aglycone, and between H-6′′′ of Glc (III) and carbonyl carbon of the HMG moiety).

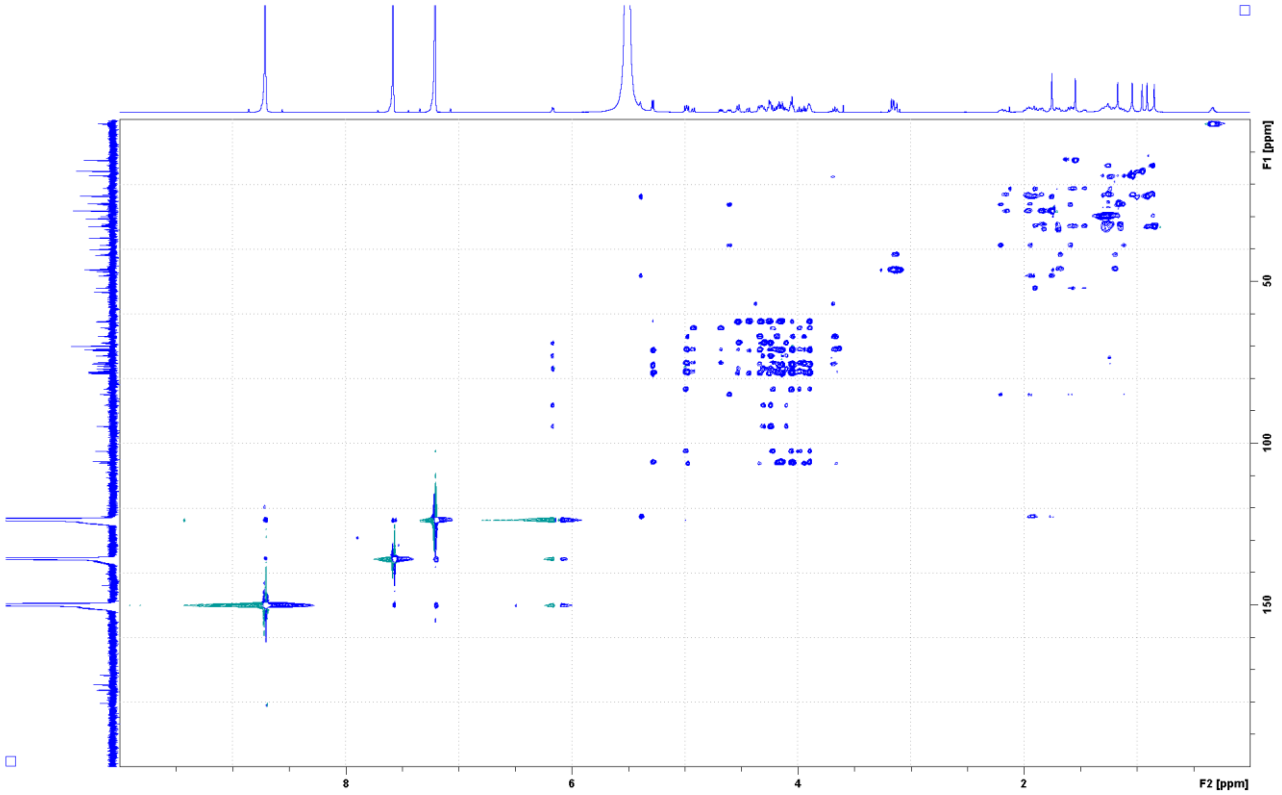

Figure S59. HSQC-TOCSY spectrum of **6**.

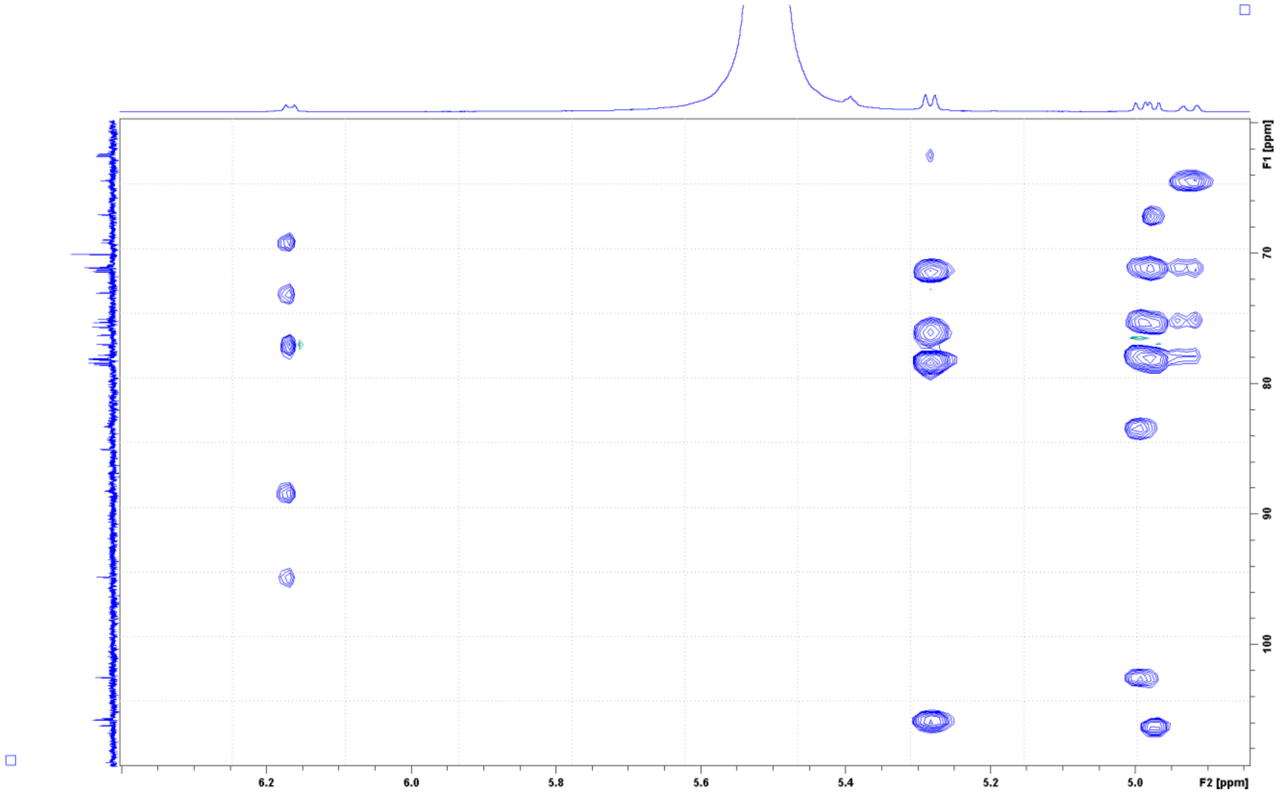

Figure S60. HSQC-TOCSY spectrum of **6** (expanded for anomeric region).

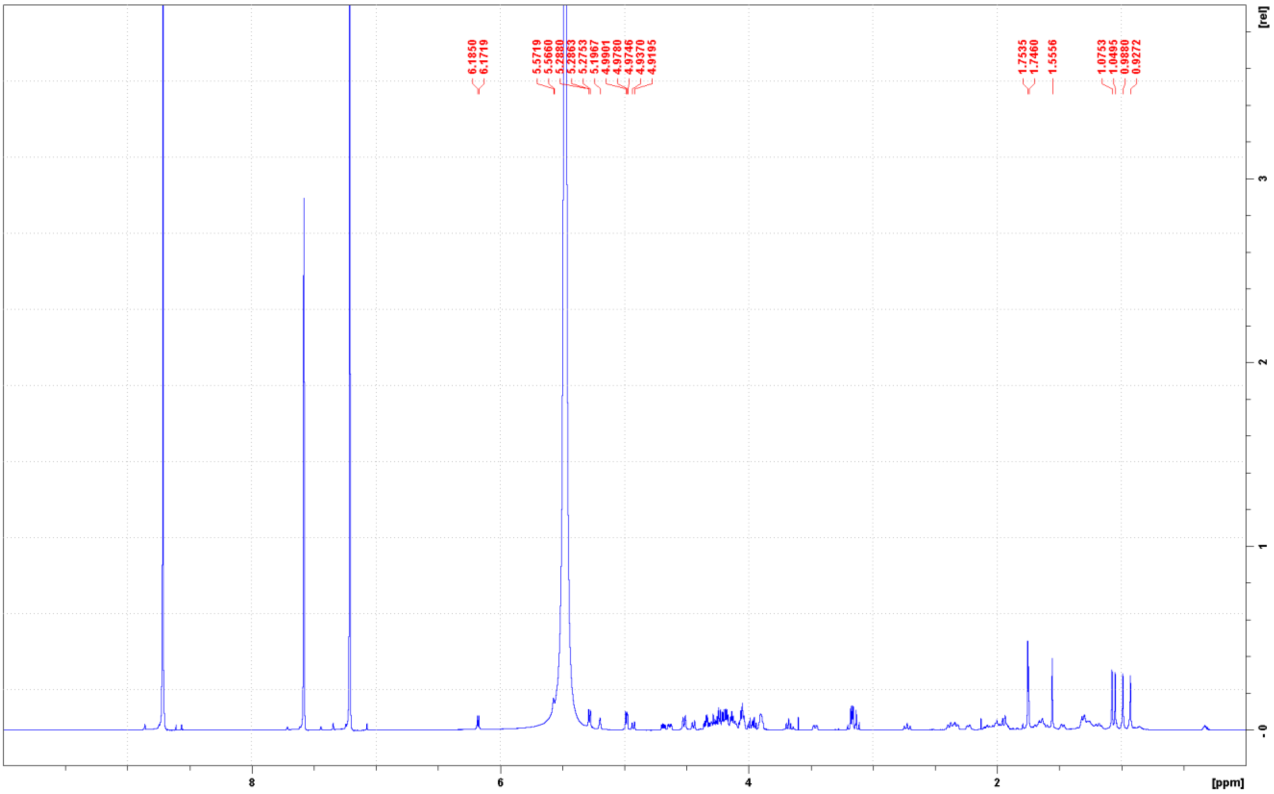

Figure S61. ^{1}H-NMR spectrum of **7**.

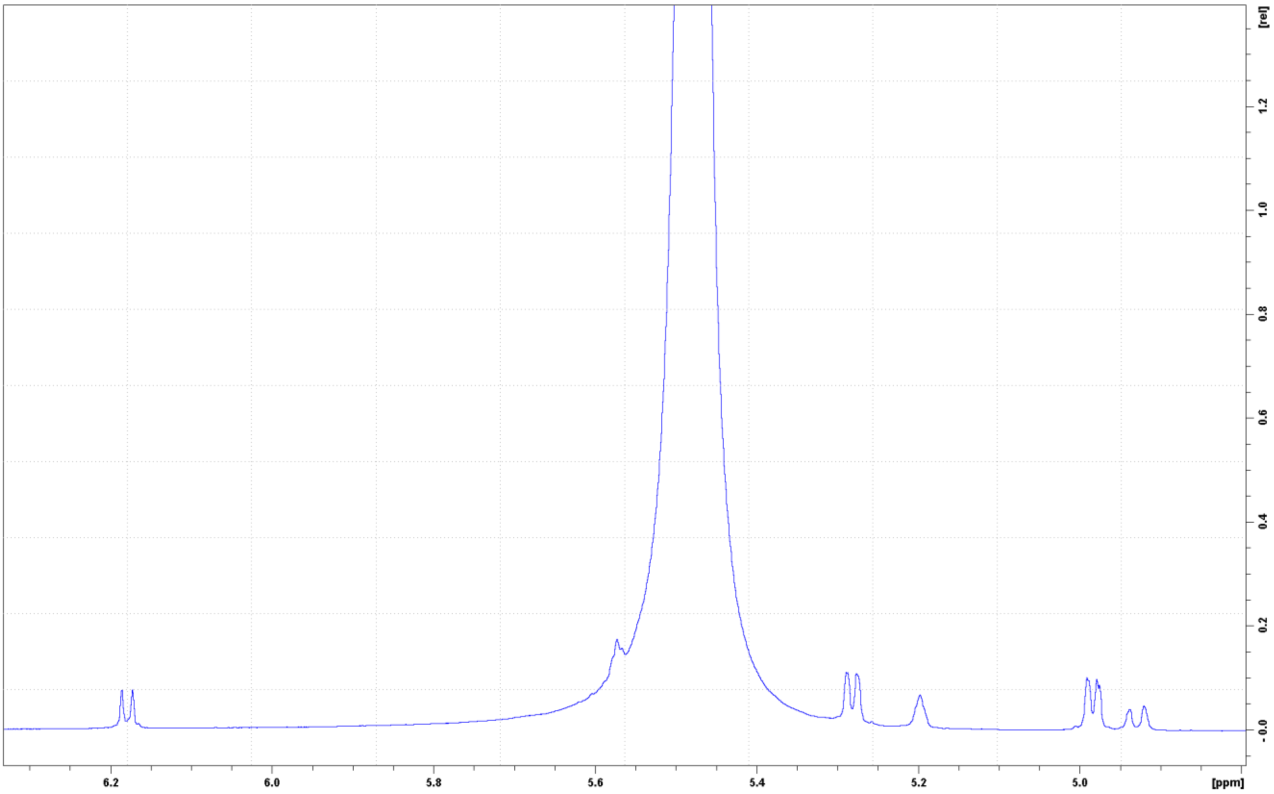

Figure S62. ^{1}H-NMR spectrum of **7** (expanded for anomeric region).

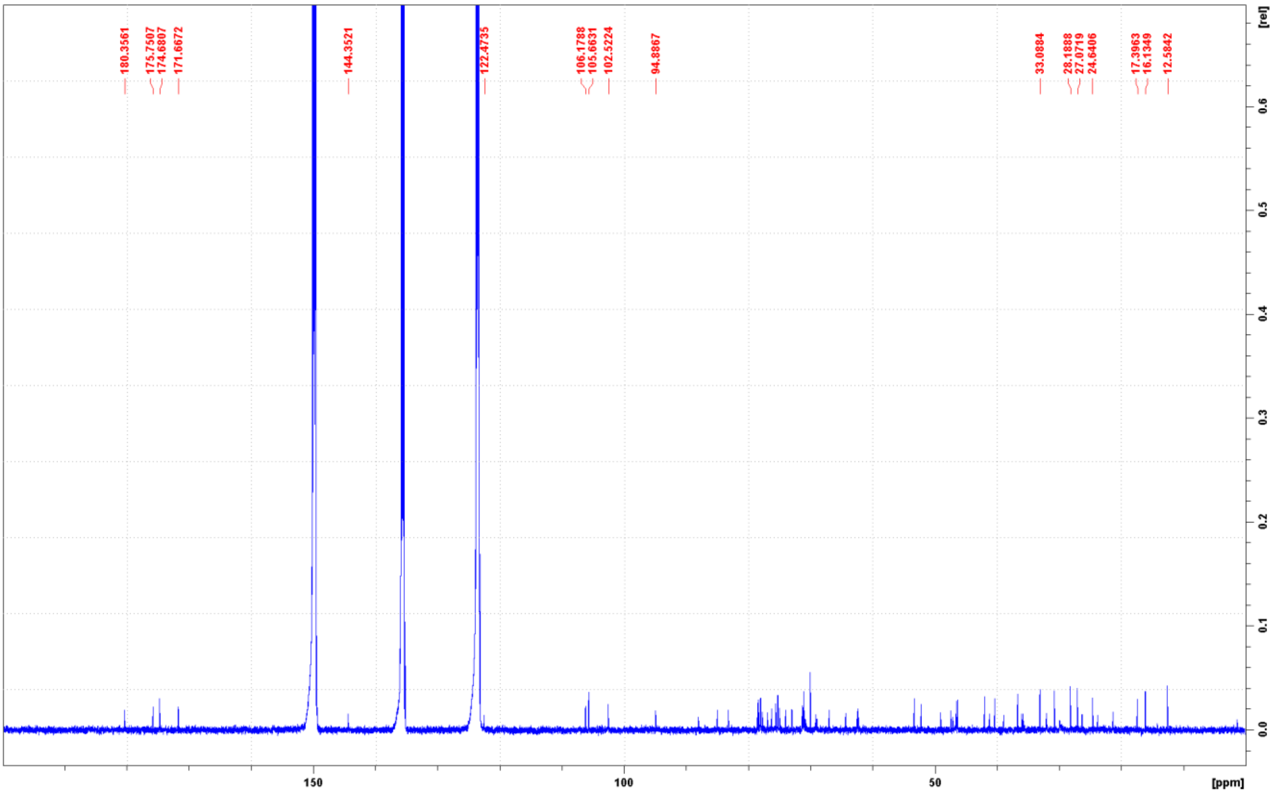

Figure S63. ^{13}C-NMR spectrum of **7**.

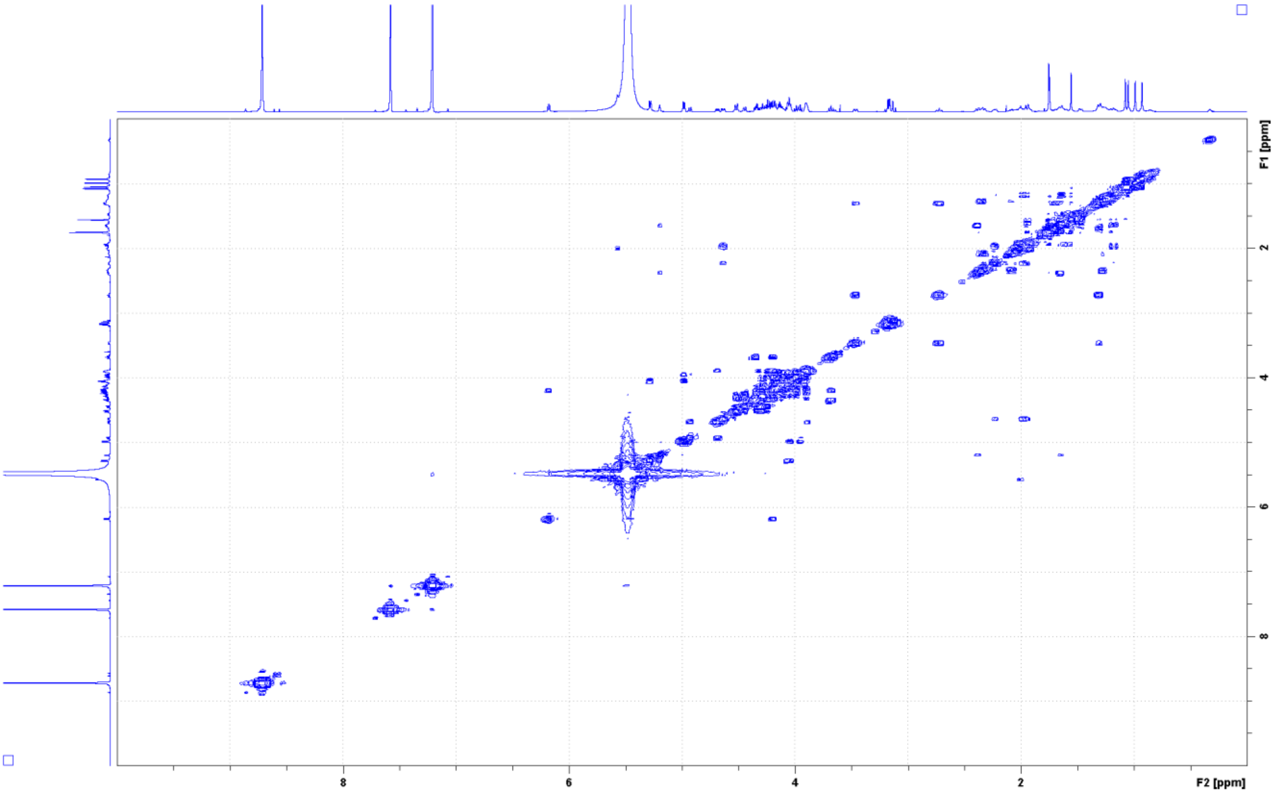

Figure S64. ^{1}H-^{1}H COSY spectrum of **7**.

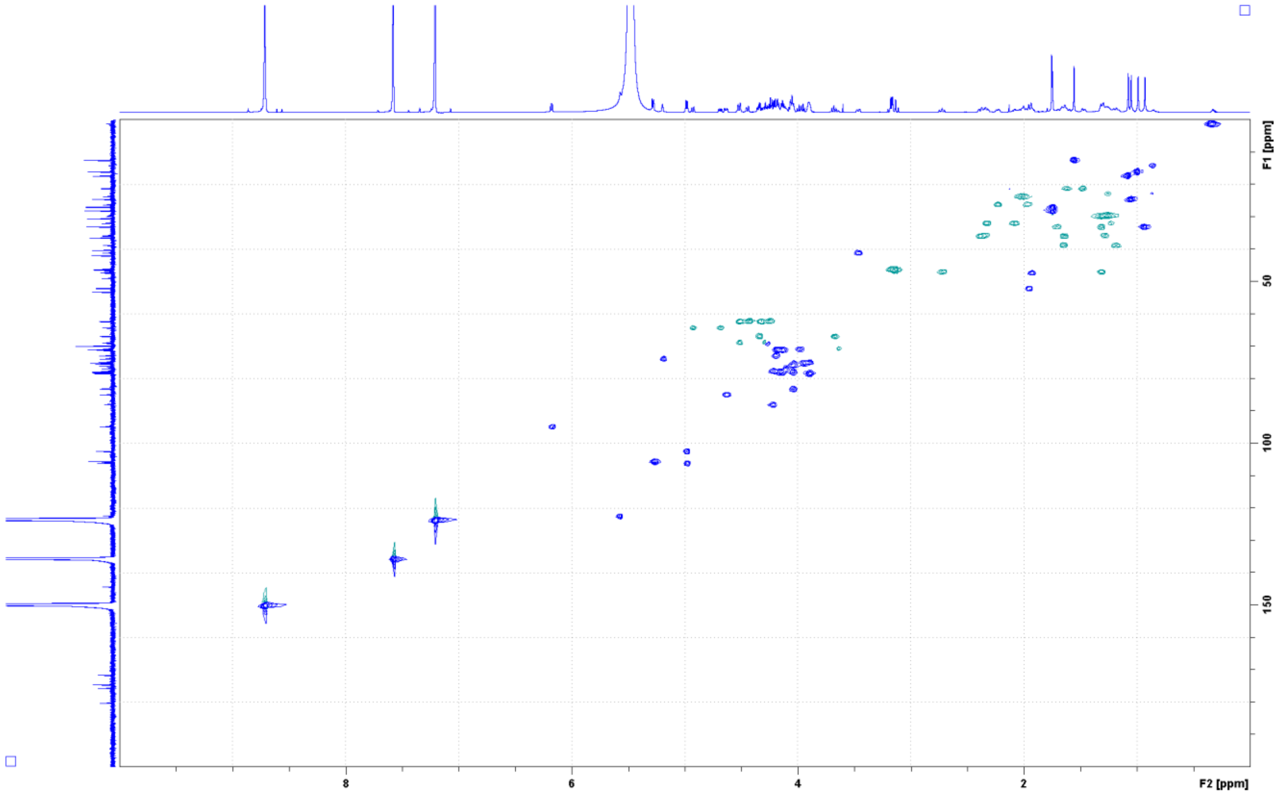

Figure S65. HSQC spectrum of **7**.

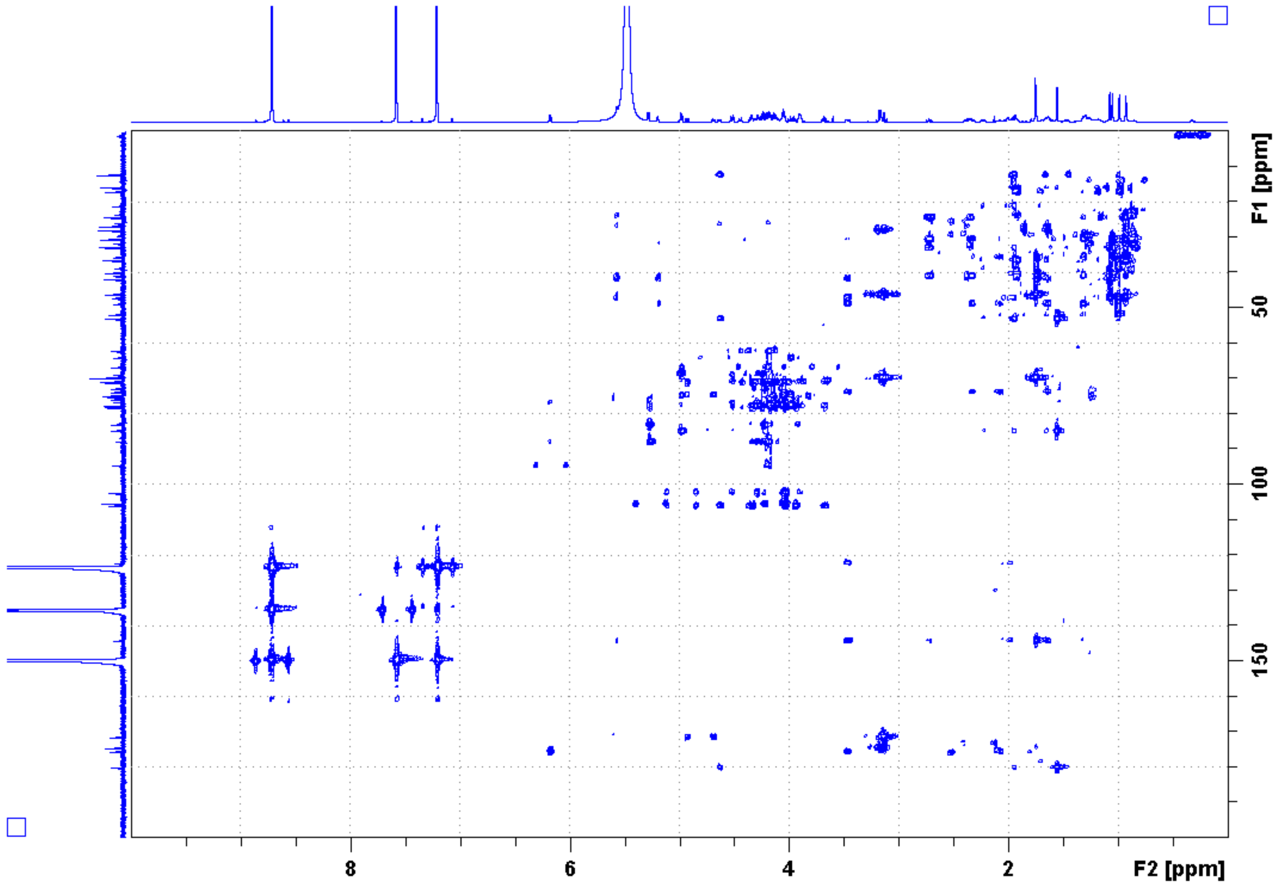

Figure S66. HMBC spectrum of **7**.

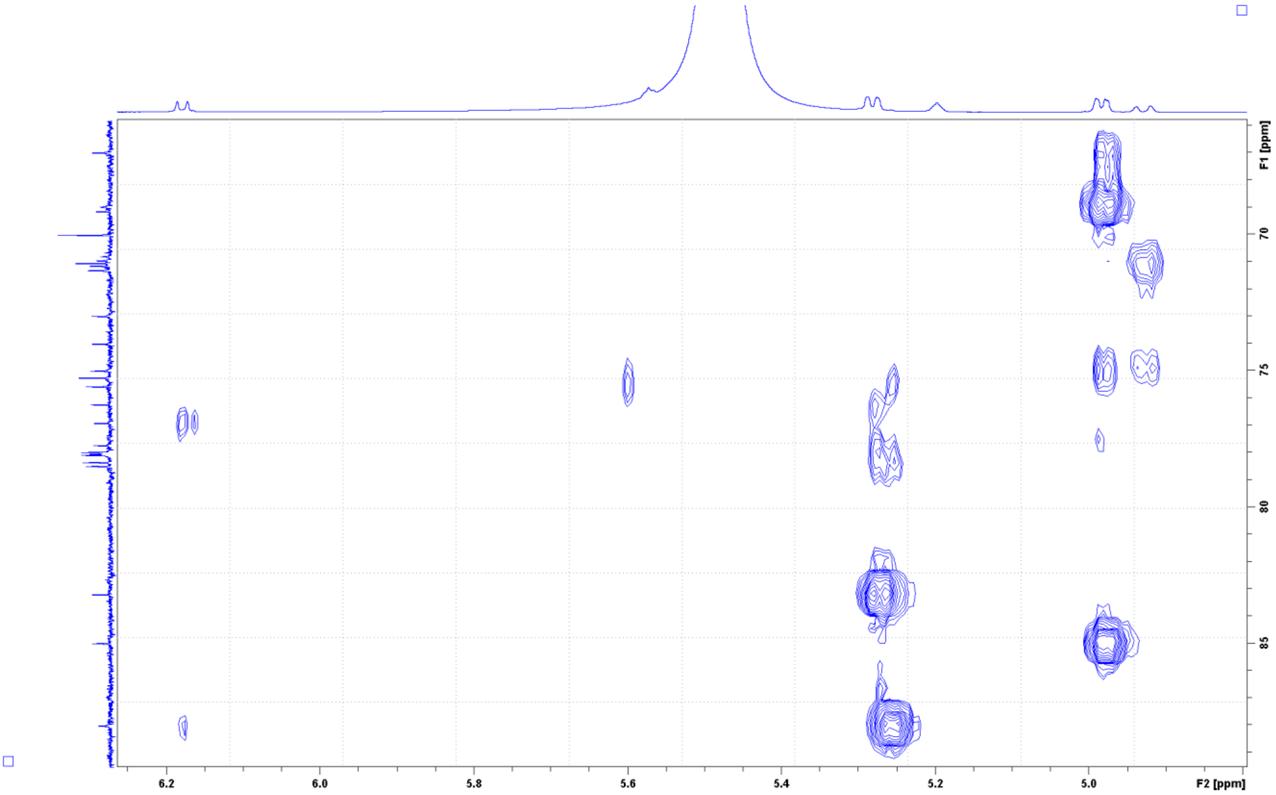

Figure S67. HMBC spectrum of **7** (expanded for anomeric region).

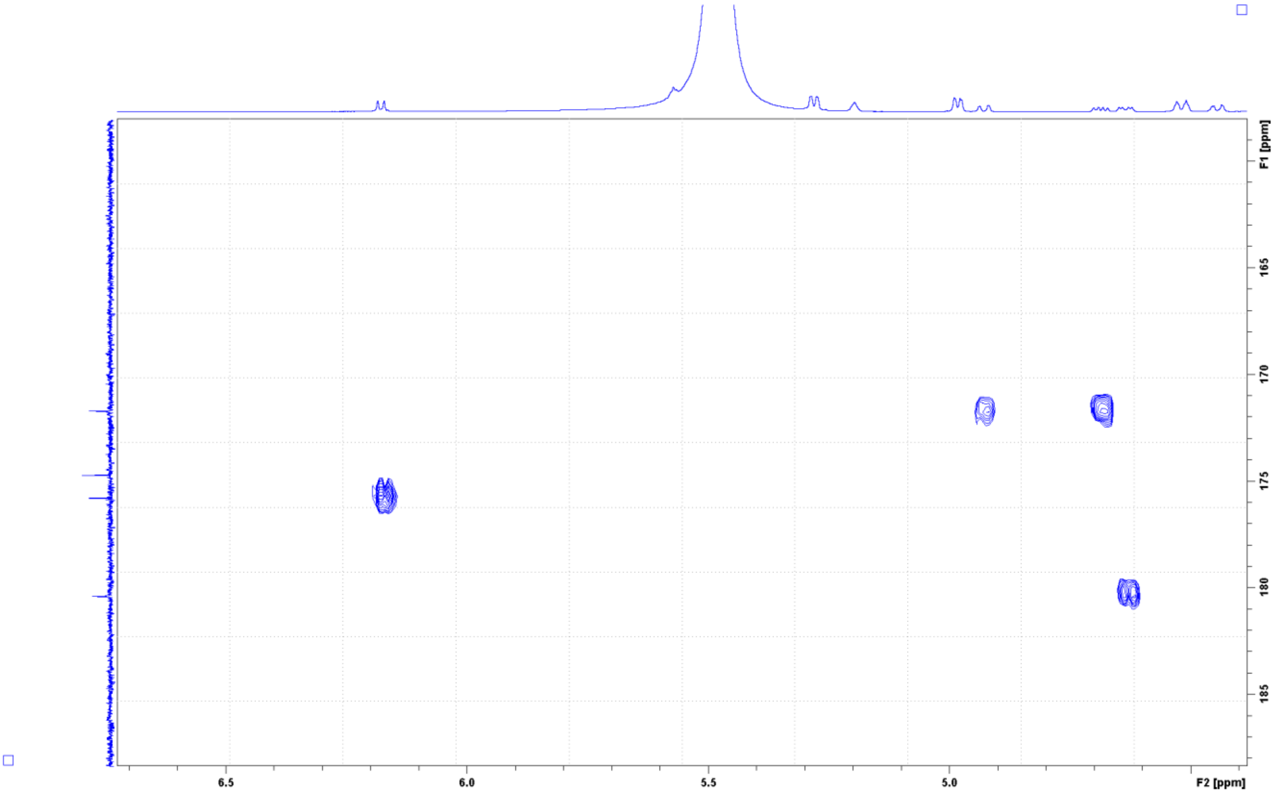

Figure S68. HMBC spectrum of **6** (expanded for between H-1'' of Glc (I) and C-28 of the aglycone, and between H-6''' of Glc (III) and carbonyl carbon of the HMG moiety).

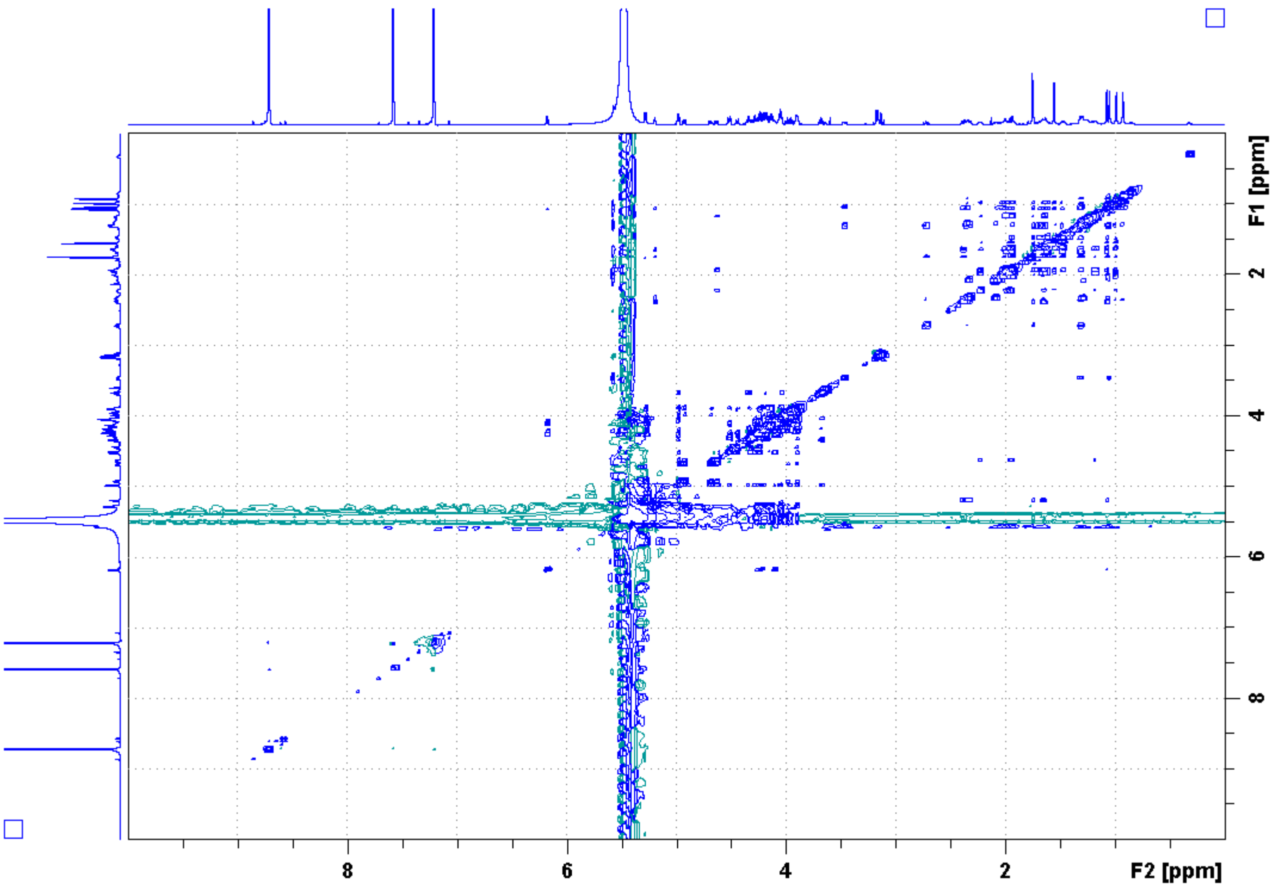

Figure S69. NOESY spectrum of **7**.

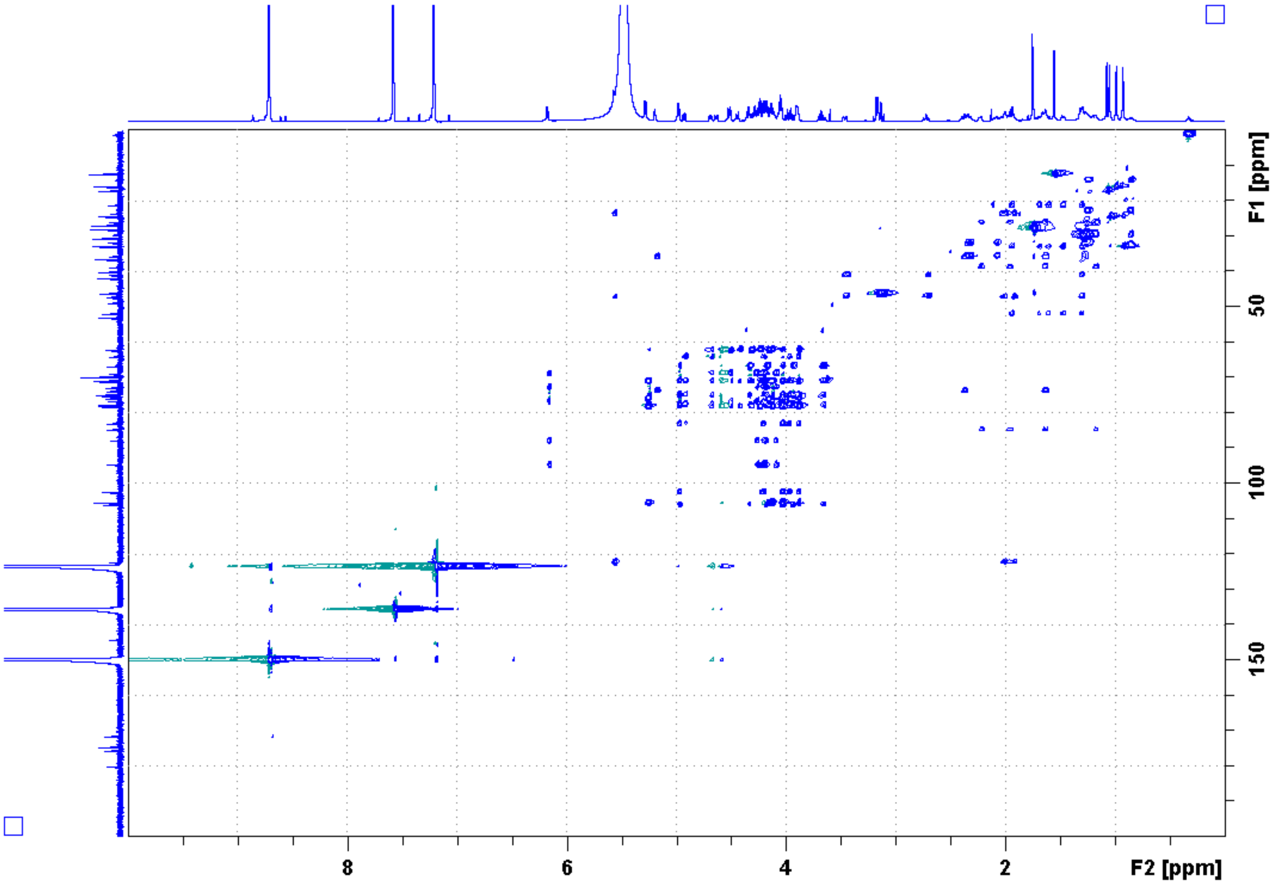

Figure S70. HSQC-TOCSY spectrum of **7**.

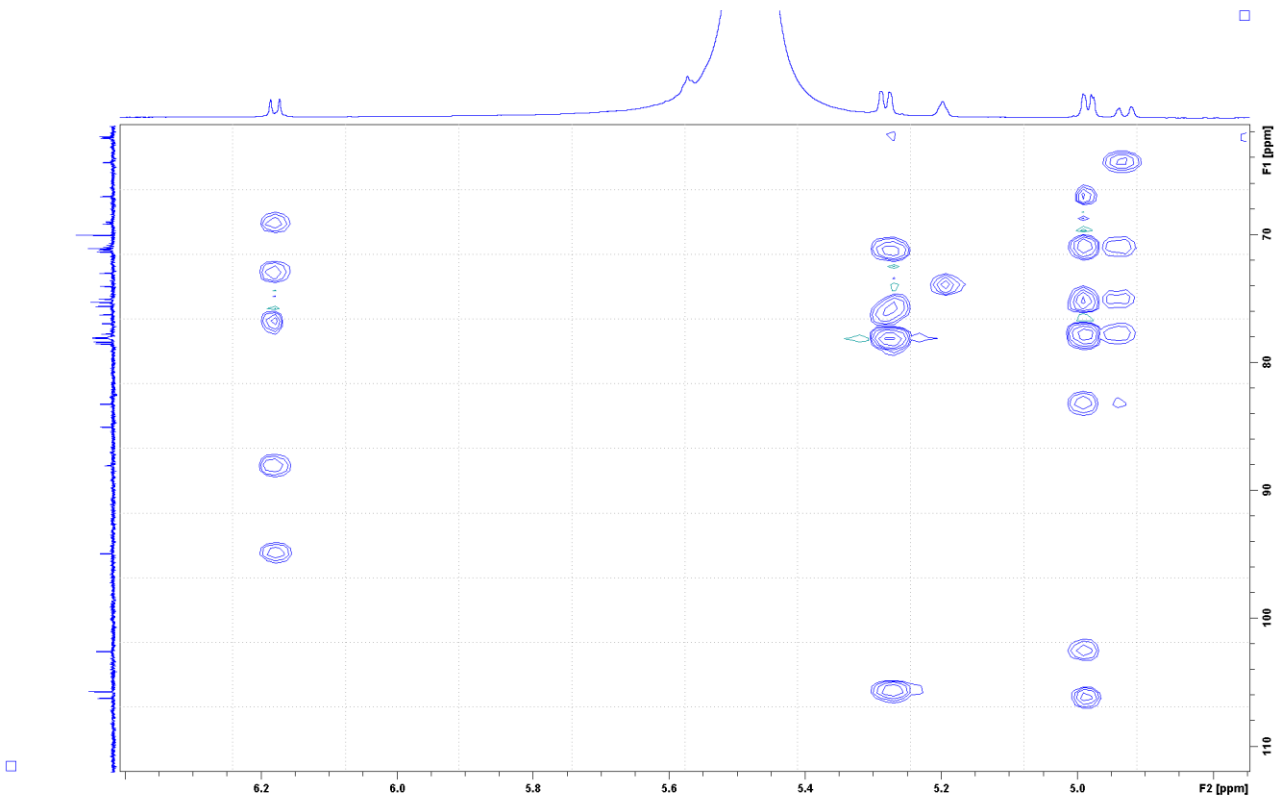

Figure S71. HSQC-TOCSY spectrum of **7** (expanded for anomeric region).
